# Efficient Synthesis and Anti-Fungal Activity of Oleanolic Acid Oxime Esters

**DOI:** 10.3390/molecules18033615

**Published:** 2013-03-21

**Authors:** Hanqing Zhao, Minjie Zhou, Lifeng Duan, Wei Wang, Jianjun Zhang, Daoquan Wang, Xiaomei Liang

**Affiliations:** Department of Applied Chemistry, China Agricultural University, Beijing 100193, China

**Keywords:** oleanolic acid, oxime ester, glucosamine-6-phosphate synthase, anti-fungal activity

## Abstract

In order to develop potential glucosamine-6-phosphate synthase inhibitors and anti-fungal agents, twenty five oleanolic acid oxime esters were synthesized in an efficient way. The structures of the new compounds were confirmed by MS, HRMS, ^1^H-NMR and ^13^C-NMR. Preliminary studies based on means of the Elson-Morgan method indicated that many compounds exhibited some inhibitory activity of glucosamine-6-phosphate synthase (GlmS), and the original fungicidal activities results showed that some of the compounds exhibited good fungicidal activities towards *Sclerotinia sclerotiorum (Lib.) de Bary*, *Rhizoctonia solani Kuhn* and *Botrytis cinerea Pers* at the concentration of 50 µg/mL. These compounds would thus merit further study and development as antifungal agents.

## 1. Introduction

Glucosamine-6-phosphate synthase (GlmS) is the first enzyme of the hexosamine biosynthetic pathway [1]. This enzyme catalyzes the reaction of D-fructose-6P (Fru6P) with glutamine to afford D-glucosamine-6P (GlcN6P) and glutamate. As a checkpoint of UDP-GlcNAc synthesis, it plays a key role in the biosynthesis of the bacterial peptidoglycan, the lipopolysaccharide of Gram-negative bacteria, chitin, and mannoproteins of the fungal cell wall [2,3].

The molecular mechanism of the reaction catalyzed by glucosamine-6-phosphate synthase is complex and involves amide bond cleavage followed by ammonia channeling and sugar isomerization [4]. It is an irreversible reaction and the sole biosynthetic route to GlcN-6-P known to date [5,6]. Although the enzyme is also present in mammalian systems, there are substantial differences in physiological consequences of GlcN-6-P synthase inhibition between fungi and mammals, thus it constitutes a firm molecular basis for the selective toxicity of specific enzyme inhibitors. Recently, this enzyme has been proposed as a good and promising target for new antifungal agents [7]. Like the most powerful GlmS inhibitors such as arabinose-5-phosphate oxime, 5-methylenephosphono-D-arabino hydroximino- lactone, *N^3^*-(4-methoxyfumaroyl)-l-2,3-diaminopropanoic acid (FMDP) and 2-amino-2-deoxy- D-glucitol-6-phosphate (ADGP), these compounds exhibit very poor, if any, antifungal activity because of the restriction due to the highly inefficient uptake of these compounds by an unidentified active transport system and apparent inability to cross the membrane by free diffusion [8].

**Figure 1:**
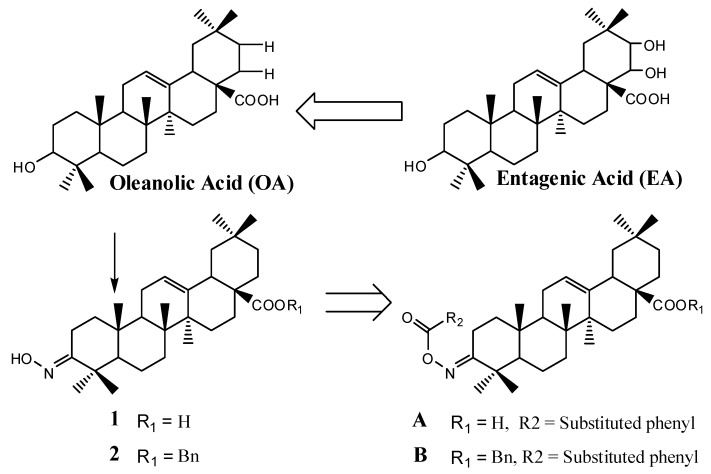
Structure of the entagenic acid (EA) and target compounds (**A**, **B**).

Triterpenes are widely distributed in Nature, and they have attracted much attention due to their broad spectrum of biological activities. Oleanolic acid (**OA**, Figure 1) is one of the most important triterpenes, which has been in active clinical use as an anti-hepatitis drug in China for over 20 years, and possesses some attractive biological activities, including protection of the liver against toxic injury [9,10,11], anti-inflammation [12], anti-HIV [13,14], anti-tumor [15,16], anti-hyperglycemia [17] and anti-cancer [18], *etc.*

In 2011, Shimoga *et al*. reported that entagenic acid (**EA**, Figure 1) showed a high antibacterial activity against *B. cereus* and *B. subtilis*, with a minimal inhibitory concentration of 200 μg mL^−1^ and possessed good glucosamine-6-phosphate synthase inhibition activity in molecular docking studies with minimum docking energy −9.22 kJ mol^−1^, binding energy −9.28 kJ mol^−1^ and inhibition constant 1.57e−007. The inhibition constant of streptomycin was 3.86e−005 [19]. As there is a good structural similarity between entagenic acid and oleanolic acid, which possess various important bioactivities [17,20], we rationalized that **OA** derivatives will have potential GlmS inhibitory activity on the basis of analog synthesis and sub-structure ligation [21]. In an ongoing project for the discovery of novel environmentally friendly antifungal agents from **OA** derivatives [22], we incorporated the structure of oxime ester, an activity group in the field of pesticides, into oleanolic acid. Twenty five new oleanolic acid oxime esters compounds (A/B, Figure 1) were efficiently synthesized, their enzyme inhibitory activities towards *Candida albicans* GlcN-6-P synthase and fungicidal activities against *Sclerotinia sclerotiorum (Lib.) de Bary*, *Rhizoctonia solani Kuhn*, *Botrytis cinerea Pers*, *Phytophthora parasitica Dast*, *Rice blas* and *Fusarium wilt* were evaluated. We report herein the preliminary results of the study. 

## 2. Results and Discussion

### 2.1. Chemistry

**Scheme 1:**
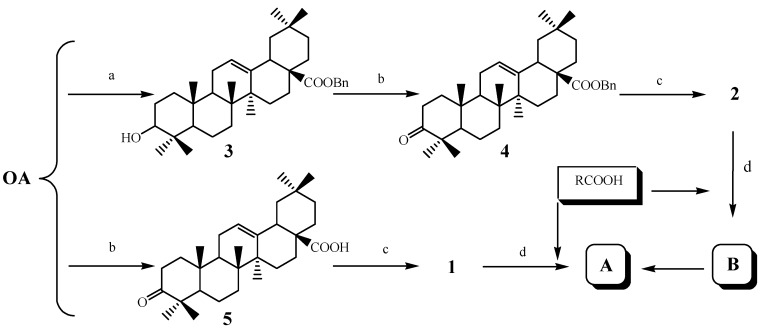
Synthetic routes to the compounds **A** and **B**.

As shown in Figure 1, we envisioned that the target compounds **A** and **B** could be synthesized from the intermediates **1** [23] or **2** [24]. As shown in Scheme 1, we envisioned that the target compounds **A** and **B** could be synthesized from the synthon **2**, and the benzyl group was chosen as the carboxylic acid protective group in order to study the importance of the COOH-group in the biological activity and avoid difficulties in the final deprotection to obtain **A**.

*Reagents and conditions*: (a) BnBr, K_2_CO_3_, DMF, r.t., 4 h, 98% for **3**; (b) Ac_2_O, PDC, CH_2_Cl_2_, reflux 3–4 h, 95% for **4**, 93% for **5**; (c) NH_2_OH·HCl, Py, 80 °C, 1 h, 96% for **1**, 94% for **2**; (d) DCC, CH_2_Cl_2_, reflux 8–14 h, 84%–93% for **A**, 70%–90% for **B**.

Firstly, benzylation of **OA** with benzyl bromide and K_2_CO_3_ in DMF provided the benzyl oleanolic acid **3** quantitatively; then oxidation of C-3-OH of **3** with pyridinium dichromate (PDC) in CH_2_Cl_2_, followed by oximation with NH_2_OH·HCl according to the reported method [23] afforded intermediate **2** in 94% yield. Condensation of **2** with substituted carboxylic acids provided the desired benzyl oleanolic acid 3-oxime esters **B**. Initially, we tried to synthesize the target compound **A** from **B** with Pd/C in MeOH/CH_2_Cl_2_ at 25 °C in the presence of hydrogen. However, instead of getting the desired compound **A**, the compound **1** was obtained as the main product, as confirmed by its ^1^H-NMR spectrum, showing the characteristic signals identical to the published data [23]. Later on, compound **1** was prepared according to the reported procedures [23], and the target compounds **A** were obtained directly from **1** in high yields. 

The structures of **A/B** were confirmed from their ^1^H-NMR, ^13^C-NMR spectra and HRMS, showing the characteristic signals such as a multiplet at *δ* 5.08 ppm for CH_2_C_6_H_5_ of **B**, a single peak at about *δ* 5.29 ppm for H-12 of **A/B**. The physical data of the target compounds are given in Table 1.

**Table 1:** Physical Data of Compounds **A** and **B**.

Compd.	R2	Formula	Status	m.p./°C	Yield (%)
A-01	4-Cl-C_6_H_4_-	C_37_H_50_ClNO_4_	White foamy solid	98–100	93
A-02	2,4-Cl_2_-C_6_H_4_-	C_37_H_49_Cl_2_NO_4_	White foamy solid	78–80	90
A-03	3-Cl-C_6_H_4_-	C_37_H_50_ClNO_4_	White foamy solid	58–60	91
A-04	4-NO_2_-C_6_H_4_-	C_37_H_50_N_2_O_6_	White foamy solid	78–80	84
A-05	1-Naphthyl-CH_2_-	C_42_H_55_NO_4_	White foamy solid	73–75	86
A-06	4-Cl-C_6_H_4_OCH_2_-	C_38_H_52_ClNO_5_	White foamy solid	70–72	87
A-07	2-F-C_6_H_4_-	C_37_H_50_FNO_4_	White foamy solid	88–90	80
A-08	4-Br-C_6_H_4_-	C_37_H_50_BrNO_4_	White foamy solid	120–122	90
A-09	3-Pyridyl-	C_36_H_50_N_2_O_4_	White foamy solid	160–162	91
A-10	2-Furan-	C_35_H_49_NO_5_	White foamy solid	96–98	86
B-01	4-Cl-C_6_H_4_-	C_44_H_56_ClNO_4_	White foamy solid	72–76	80
B-02	2,4-Cl_2_-C_6_H_4_-	C_44_H_55_C_l2_NO_4_	White foamy solid	134–136	83
B-03	3-Cl-C_6_H_4_-	C_44_H_56_ClNO_4_	White foamy solid	68–72	79
B-04	4-NO_2_-C_6_H_4_-	C_44_H_56_N_2_O_6_	White foamy solid	71–74	85
B-05	1-Naphthyl-CH_2_-	C_4__9_H_61_NO_4_	White foamy solid	136–138	75
B-06	4-Cl-C_6_H_4_OCH_2_-	C_45_H_58_ClNO_5_	White foamy solid	54–56	72
B-07	2-F-C_6_H_4_-	C_44_H_56_FNO_4_	Viscous liquid	—	75
B-08	4-Br-C_6_H_4_-	C_44_H_56_BrNO_4_	White foamy solid	74–78	78
B-09	3-Pyridyl-	C_43_H_56_N_2_O_4_	White foamy solid	70–72	81
B-10	2-Furan-	C_42_H_55_NO_5_	White foamy solid	68–70	71
B-11	3- NO_2_-C_6_H_4_-	C_44_H_56_N_2_O_6_	White foamy solid	76–80	84
B-12	3,5-( NO_2_)_ 2_C_6_H_4_-	C_44_H_55_N_3_O_8_	White foamy solid	68–70	90
B-13	2-Cl-C_6_H_4_-	C_44_H_56_ClNO_4_	White foamy solid	116–118	76
B-14	2-Pyridyl-	C_43_H_56_N_2_O_4_	White foamy solid	80–84	79
B-15	C_6_H_5_-	C_44_H_57_NO_4_	White foamy solid	78–81	70

### 2.2. Bioassay of Enzyme Inhibitory Activities [25,26,27,28]

Inhibitory activity of all the synthesized compounds towards *Candida albicans* GlcN-6-P synthase was evaluated using the further optimized Elson-Morgan method [25,26,27,29]. The absorption value of the solution was measured at 585 nm, and then the concentration was counted by the specification curve which was determined thanks to the relation between the absorption value and the concentration of glucosamine-6-phosphate. Finally the enzyme inhibition rate was calculated according to formula (1):

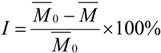
(1)
where *I* is the inhibition rate, *M*_0_ is the average concentration of glucosamine-6-phosphate in the blank test, and *M* is the average concentration of glucosamine-6-phosphate in the presence of target compounds. The inhibition rates were given in Table 2 at 0.35 mM.

Many compounds of **A** series and **B** series exhibited better enzyme inhibitory activities than **OA**, but this fact is not as obvious as possible since our work reveals that some compounds **B** exhibited less activity. Compounds **A-02**, **A-03**, **B-06**, **B-12** and **B-13** are more active against glucosamine-6-phosphate synthase than the other compounds. On the whole, the enzyme inhibitory activity of **A** series of compounds is superior to the **B** series, which may be associated with a better structural similarity between **EA** and the target compounds.

**Table 2:** Enzyme inhibition rates of compounds **A** and **B** at 0.35 mM.

Compd No.	Inhibition Rate (%)	Compd No.	Inhibition Rate (%)
**OA**	22.4	**B-03**	19.8
**A-01**	29.4	**B-04**	16.2
**A-02**	37.2	**B-05**	20.2
**A-03**	40.8	**B-06**	34.2
**A-04**	19.2	**B-07**	28.2
**A-05**	30.8	**B-08**	12.7
**A-06**	29.1	**B-09**	19.3
**A-07**	22.9	**B-10**	13.2
**A-08**	24.8	**B-11**	16.4
**A-09**	21.0	**B-12**	33.0
**A-10**	20.5	**B-13**	33.1
**B-01**	8.7	**B-14**	12.5
**B-02**	12.2	**B-15**	13.8

### 2.3. Bioassay of Fungicidal Activities [28]

Fungicidal activities of the target compounds against *Sclerotinia sclerotiorum* (Lib.) de Bary, *Rhizoctonia solani* Kuhn, *Botrytis cinerea* Pers, *Phytophthora parasitica* Dast, rice blast and fusarium wilt were evaluated using the mycelium growth rate test [28]. The diameter of the mycelia was measured and the inhibition rate was calculated according to formula (2):

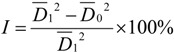
(2)
where *I* is the inhibition rate, *D*_1_ is the average diameter of mycelia in the blank test, and *D*_0_ is the average diameter of mycelia in the presence of target compounds: The inhibition rates of compounds **A** and **B** against the six fungi at 50 µg/mL are given in Table 3.

Compounds **A**–**B** exhibited more fungicidal activity against *R. solani,* rice blast and *S. sclerotiorum* than the other fungi. The fungicidal activity of the **B** series is better than that of the **A** series. Compounds **A-02**, **A-03**, **A-05**, **B-03**, **B-06**, **B-07** and **B-09** exhibited good fungicidal activity, consistent with their enzyme inhibitory activities. 

**Table 3:** Inhibition rates of compounds **A**–**B** against six fungi.

Compd. No. (CAU2012)	Inhibition rate (%)					
	*S. sclerotiorum*	*Phytophthora parasitica* Dast	*B. cinerea*	*R. solani*	*Rice blast*	*Fusarium wilt*
**A-01**	28.2	1.8	7.8	38.0	31.2	11.7
**A-02**	61.2	42.1	8.4	36.2	29.5	14.9
**A-03**	26.5	3.1	7.8	42.7	24.2	3.2
**A-04**	53.5	49.3	32.1	32.1	49.1	15.8
**A-05**	28.6	14.2	25.8	73.0	21.0	6.5
**A-06**	29.0	4.0	20.7	33.5	21.0	7.5
**A-07**	49.7	67.6	12.4	45.2	33.1	6.1
**A-08**	27.8	2.0	13.4	18.6	24.2	7.5
**A-09**	24.8	3.2	1.4	16.6	25.1	6.1
**A-10**	30.3	2.3	24.9	5.5	27.9	5.1
**B-01**	71.1	9.4	68.8	79.2	39.3	28.5
**B-02**	71.1	12.0	49.4	63.5	36.9	30.1
**B-03**	72.6	23.5	71.1	93.6	78.5	36.7
**B-04**	68.2	25.9	40.5	41.4	74.3	31.3
**B-05**	68.9	17.4	66.4	84.1	51.9	28.5
**B-06**	67.4	18.1	42.9	73.7	55.2	25.9
**B-07**	73.3	17.7	59.5	86.1	52.5	26.9
**B-08**	67.8	21.7	59.9	44.1	36.2	31.3
**B-09**	74.4	25.3	69.1	63.6	68.0	29.2
**B-10**	68.2	23.5	57.7	54.0	36.0	28.7
**B-11**	64.5	35.6	45.7	56.6	63.3	21.4
**B-12**	67.5	28.2	41.4	69.5	53.6	28.2
**B-13**	66.7	52.2	46.2	51.7	60.0	24.6
**B-14**	67.1	56.5	62.7	77.6	54.9	30.8
**B-15**	71.0	38.2	39.0	33.8	85.5	28.7
**Chlorothalonil**	92.8	94.8	98.2	98.5	89.5	96.2
**Sanmate**	99.3	68.9	64.7	100	73.7	96.1
**OA**	20.5	13.5	1.0	25.3	20.1	7.5

## 3. Experimental

### 3.1. General Methods

Solvents were purified in the usual way. All reactions were monitored by TLC analysis performed on silica gel HF with detection by charring with 30% (v/v) H_2_SO_4_ in CH_3_OH or by UV detection. Column chromatography was conducted by elution of a column (8 × 100, 16 × 240, 18 × 300, 35 × 400 mm) of silica gel (200–300 mesh) with EtOAc-PE (petroleum ether, b.p. 60–90 °C) as the eluent. NMR spectra (300/75 MHz, δ, ppm) were recorded on a Varian XL-300 spectrometer with TMS as the internal standard. Elemental analysis was performed on a Yanaco CHN Corder MF-3 automatic elemental analyzer. Mass spectra were recorded with a VG PLATFORM mass spectrometer using the electrospray ionization (ESI) mode.

### 3.2. Chemical Synthesis

*Oleanolic acid 3-oxime ester* (**A-01**). 4-Chlorobenzoic acid (0.66 g, 4.2 mmol) and *N,N′*-dicyclohexylcarbodiimide (DCC, 1 g, 5 mmol) were successively added to a soln. of oleanate 3-oxime **1** (1.68 g, 3.5 mmol) which was prepared according to the reported method [12] in CH_2_Cl_2_ (50 mL), Then the reaction mixture was refluxed for 8–14 h at the end of which time TLC (4:1 petroleum ether/EtOAc) indicated that the reaction was complete. The reaction mixture was filtered, the soln. was concentrated, and the residue was subjected to column chromatography (6:1 petroleum ether/EtOAc) to give the desired product **A-01** (1.98 g, 93%) as a white foamy solid. ^1^H-NMR (CDCl_3_): *δ* 8.00–7.42 (m, 4H, Ar-H), 5.28 (br s, 1 H, H-12), 3.05–3.01 (m, 1H), 2.85–2.80 (m, 1H), 2.48-2.41 (m, 1H), 1.34, 1.19, 1.13, 1.06, 0.93, 0.90, 0.80 (s, 7 × 3H, CH_3_); ^13^C-NMR (CDCl_3_): 184.0 (COOH), 176.4 (COONC), 163.4 (COONC), 143.8 (C-13), 139.4, 130.9, 130.9, 128.8, 128.8, 128.1 (aromatic carbons), 122.2 (C-12), 55.8, 47.1, 46.6, 45.8, 41.7, 41.0, 39.3, 38.7, 37.1, 33.8, 33.0, 32.4, 32.3, 30.6, 29.7, 27.6, 27.2, 25.8, 23.5, 23.4, 23.2, 22.9, 19.9, 18.9, 17.0, 15.1 (7 × CH_3_); Anal. Calcd for C_37_H_50_ClNO_4_: C, 73.06; H, 8.29; N, 2.30. found: C, 73.27; H, 8.05; N, 2.51; HRMS calcd for C_37_H_50_ClNO_4_ (M+H)^+^: 608.35011, found: 608.34985. 

*Oleanolic acid 3-oxime ester* (**A-02**). The reaction was run similarly to that used to synthesize **A-01**. A white foamy solid **A-02** was obtained in 90% yield. ^1^H-NMR (CDCl_3_): *δ* 8.00–7.42 (m, 4H, Ar-H), 5.28 (br s, 1 H, H-12), 3.05–3.01 (m, 1H), 2.85–2.80 (m, 1H), 2.48–2.41 (m, 1H), 1.34, 1.19, 1.13, 1.06, 0.93, 0.90, 0.80 (s, 7 × 3H, CH_3_); ^13^C-NMR (CDCl_3_): 184.3 (COOH), 176.5 (COONC), 163.1 (COONC), 143.7 (C-13), 138.2, 134.3, 132.4, 130.8, 128.4, 127.0 (aromatic carbons), 122.2 (C-12), 55.8, 47.1, 46.5, 45.8, 41.6, 40.9, 39.3, 38.7, 37.0, 33.7, 33.0, 32.4, 32.2, 30.6, 29.6, 27.6, 27.0, 25.8, 23.5, 23.4, 23.0, 22.8, 20.2, 18.9, 17.0, 15.1 (7 × CH_3_); Anal. Calcd for C_37_H_49_Cl_2_NO_4_: C, 69.15; H, 7.68; N, 2.18. found: C, 69.35; H, 7.47; N, 2.33; HRMS calcd for C_37_H_50_ClNO_4_ (M+H)^+^: 642.31114, found: 642.31079. 

*Oleanolic acid 3-oxime ester* (**A-03**). The reaction was run similarly to that used to synthesize **A-01**. A white foamy solid **A-03** was obtained in 91% yield. ^1^H-NMR (CDCl_3_): *δ* 8.01–7.37 (m, 3H, Ar-H), 5.28 (br s, 1 H, H-12), 3.06–3.01 (m, 1H), 2.86–2.80 (m, 1H), 2.47–2.45 (m, 1H), 1.34, 1.19, 1.13, 1.07, 0.93, 0.90, 0.80 (s, 7 × 3H, CH_3_); ^13^C-NMR (CDCl_3_): 184.3 (COOH), 176.4 (COONC), 163.0 (COONC), 143.7 (C-13), 134.5, 133.0, 131.4, 129.7, 129.4, 127.6 (aromatic carbons), 122.2 (C-12), 55.8, 47.1, 46.5, 45.8, 41.6, 40.9, 39.3, 38.6, 37.0, 33.7, 33.0, 32.4, 32.2, 30.6, 29.6, 27.6, 27.0, 25.8, 23.5, 23.4, 23.0, 22.8, 20.2, 18.9, 17.0, 15.1 (7 × CH_3_); Anal. Calcd for C_37_H_50_ClNO_4_: C, 73.06; H, 8.29; N, 2.30. found: C, 73.21; H, 8.43; N, 2.49; HRMS calcd for C_37_H_50_ClNO_4 _(M+H)^+^: 608.35011, found: 608.34937. 

*Oleanolic acid 3-oxime ester* (**A-04**). The reaction was run similarly to that used to synthesize **A-01**. A white foamy solid **A-04** was obtained in 84% yield. ^1^H-NMR (CDCl_3_): *δ* 8.33–8.20 (m, 4H, Ar-H), 5.33 (br s, 1 H, H-12), 3.07–3.01 (m, 1H), 2.88–2.82 (m, 1H), 2.49–2.43 (m, 1H), 1.36, 1.19, 1.15, 1.07, 0.94, 0.92, 0.83 (s, 7 × 3H, CH_3_); ^13^C-NMR (CDCl_3_): 177.0 (COOH), 172.8 (COONC), 162.3 (COONC), 150.5, 143.3 (C-13), 135.1, 130.5, 130.5, 123.6, 123.6 (aromatic carbons), 122.6 (C-12), 55.8, 48.3, 47.1, 45.6, 41.8, 41.7, 39.4, 38.7, 37.0, 33.6, 32.9, 32.3, 31.9, 30.6, 29.6, 27.4, 27.1, 25.7, 23.5, 23.2, 22.9, 22.6, 20.0, 18.9, 17.1, 15.1 (7 × CH_3_); Anal. Calcd for C_37_H_50_N_2_O_6_: C, 71.82; H, 8.14; N, 4.53. found: C, 71.97; H, 8.01; N, 4.69; HRMS calcd for C_37_H_50_ClNO_4 _(M+H)^+^: 619.37416, found: 619.37708. 

*Oleanolic acid 3-oxime ester* (**A-05**). The reaction was run similarly to that used to synthesize **A-01**. A white foamy solid **A-05** was obtained in 86% yield. ^1^H-NMR (CDCl_3_): *δ* 8.05–7.41(m, 7H, Ar-H), 5.27 (br s, 1 H, H-12), 4.20–4.18 (m, 2H, CH_2_), 2.84–2.78 (m, 1H), 2.58–2.53 (m, 1H), 1.25, 1.19, 1.09, 1.04, 0.93, 0.90, 0.74 (s, 7 × 3H, CH_3_); ^13^C-NMR (CDCl_3_): 184.2 (COOH), 175.5 (COONC), 169.3 (COONC), 143.6 (C-13), 133.7, 132.0, 130.2, 128.6, 127.9, 126.2, 125.7, 125.6, 125.3, 123.8 (aromatic carbons), 122.2 (C-12), 65.4, 55.7, 47.0, 46.5, 45.7, 41.6, 41.3, 40.9, 39.2, 38.2, 36.8, 33.7, 33.0, 32.3, 32.2, 30.6, 29.6, 27.5, 26.9, 25.8, 23.5, 23.3, 22.8, 19.2, 18.7, 16.9, 14.9 (7 × CH_3_); Anal. Calcd for C_42_H_55_NO_4_: C, 79.08; H, 8.69; N, 2.20. found: C, 79.24; H, 8.37; N, 2.42; HRMS calcd for C_37_H_50_ClNO_4 _(M+H)^+^: 638.42039, found: 638.42004. 

*Oleanolic acid 3-oxime ester* (**A-06**). The reaction was run similarly to that used to synthesize **A-01**. A white foamy solid **A-06** was obtained in 87% yield. ^1^H-NMR (CDCl_3_): *δ* 7.26–6.81 (m, 4H, Ar-H), 5.28 (br s, 1 H, H-12), 4.82 (s, 1H), 4.72(s, 1H), 2.87–2.82 (m, 1H), 2.30–2.21 (m, 1H), 1.26, 1.24, 1.11, 1.01, 0.93, 0.91, 0.78 (s, 7 × 3H, CH_3_); ^13^C-NMR (CDCl_3_): 183.9 (COOH), 175.8 (COONC), 167.8 (COONC), 156.4, 153.1, 143.6 (C-13), 129.3, 126.6, 122.1 (C-12), 116.0, 115.8 (aromatic carbons), 65.4, 55.7, 47.0, 46.4, 45.7, 41.6, 40.9, 39.2, 38.5, 36.9, 33.7, 32.9, 32.5, 32.3, 30.5, 29.6, 27.5, 27.0, 25.7, 23.4, 23.3, 22.9, 22.8, 19.4, 18.8, 16.9, 15.0 (7 × CH_3_); Anal. Calcd for C_38_H_52_ClNO_5_: C, 71.51; H, 8.21; N, 2.19. found: C, 71.35; H, 8.39; N, 2.35; HRMS calcd for C_37_H_50_ClNO_4 _(M+H)^+^: 638.36068, found: 638.35919. 

*Oleanolic acid 3-oxime ester* (**A-07**). The reaction was run similarly to that used to synthesize **A-01**. A white foamy solid **A-07** was obtained in 80% yield. ^1^H-NMR (CDCl_3_): *δ* 8.05–7.11(m, 4H, Ar-H), 5.29 (br s, 1 H, H-12), 3.12–3.07 (m, 1H), 2.85–2.82 (m, 1H), 2.49–2.38 (m, 1H), 1.34, 1.19, 1.13, 1.06, 0.93, 0.90, 0.80 (s, 7 × 3H, CH_3_); ^13^C-NMR (CDCl_3_): 184.5 (COOH), 176.1 (COONC), 163.1 (COONC), 143.6 (C-13), 134.3, 132.2, 124.0, 122.2 (C-12), 118.0, 116.9, 116.6 (aromatic carbons), 55.7, 47.0, 46.5, 45.7, 41.4, 40.9, 39.2, 38.6, 36.9, 33.7, 32.9, 32.3, 32.2, 30.5, 29.6, 27.5, 27.1, 25.7, 23.4, 23.3, 23.0, 22.7, 19.9, 18.8, 16.9, 15.0 (7 × CH_3_); Anal. Calcd for C_37_H_50_FNO_4_: C, 75.09; H, 8.52; N, 2.37. found: C, 75.29; H, 8.38; N, 2.16; HRMS calcd for C_37_H_50_ClNO_4 _(M+H)^+^: 592.37966, found: 592.37909. 

*Oleanolic acid 3-oxime ester* (**A-08**). The reaction was run similarly to that used to synthesize **A-01**. A white foamy solid **A-08** was obtained in 90% yield. ^1^H-NMR (CDCl_3_): *δ* 7.95–7.58 (m, 4H, Ar-H), 5.29 (br s, 1 H, H-12), 3.05–3.00 (m, 1H), 2.86–2.81 (m, 1H), 2.44–2.42 (m, 1H), 1.34, 1.19, 1.13, 1.06, 0.90, 0.88, 0.80 (s, 7 × 3H, CH_3_); ^13^C-NMR (CDCl_3_): 184.3 (COOH), 176.4 (COONC), 163.5 (COONC), 143.8 (C-13), 131.9, 131.8, 131.0, 131.0, 128.6, 128.1 (aromatic carbons), 122.3 (C-12), 55.8, 47.2, 45.8, 41.7, 41.6, 41.0, 39.4, 38.7, 37.1, 33.8, 33.0, 32.4, 31.9, 30.7, 29.7, 27.6, 27.2, 25.9, 23.6, 23.5, 23.2, 22.7, 19.9, 19.0, 17.0, 15.1 (7 × CH_3_); Anal. Calcd for C_37_H_50_BrNO_4_: C, 68.09; H, 7.72; N, 2.15. found: C, 68.39; H, 7.46; N, 2.35; HRMS calcd for C_37_H_50_ClNO_4 _(M+H)^+^: 652.29960, found: 652.30011. 

*Oleanolic acid 3-oxime ester* (**A-09**). The reaction was run similarly to that used to synthesize **A-01**. A white foamy solid **A-09** was obtained in 91% yield. ^1^H-NMR (CDCl_3_): *δ* 9.25–7.44 (m, 4H, Ar-H), 5.30 (br s, 1 H, H-12), 3.08–3.03 (m, 1H), 2.90–2.84 (m, 1H), 2.48–2.41 (m, 1H), 1.35, 1.21, 1.14, 1.07, 0.94, 0.91, 0.82 (s, 7 × 3H, CH_3_); ^13^C-NMR (CDCl_3_): 183.1 (COOH), 176.7 (COONC), 162.7 (COONC), 153.0, 150.1, 143.9 (C-13), 137.3, 125.9, 123.6 (aromatic carbons), 122.0 (C-12), 55.7, 47.1, 46.4, 45.8, 41.6, 41.0, 39.2, 38.6, 37.0, 33.6, 33.0, 32.4, 32.2, 30.6, 29.6, 27.6, 27.1, 25.8, 23.5, 23.4, 23.1, 22.9, 19.9, 18.9, 16.9, 15.1 (7 × CH_3_); Anal. Calcd for C_36_H_50_N_2_O_4_: C, 75.22; H, 8.77; N, 4.87. found: C, 75.07; H, 8.64; N, 4.63; HRMS calcd for C_37_H_50_ClNO_4 _(M+H)^+^: 575.38433, found: 575.38373. 

*Oleanolic acid 3-oxime ester* (**A-10**). The reaction was run similarly to that used to synthesize **A-01**. A white foamy solid **A-10** was obtained in 86% yield. ^1^H-NMR (CDCl_3_): *δ* 7.63–6.47 (m, 3H, Ar-H), 5.28 (br s, 1 H, H-12), 3.07–3.02 (m, 1H), 2.87–2.81 (m, 1H), 2.48–2.42 (m, 1H), 1.33, 1.17, 1.13, 1.05, 0.93, 0.90, 0.80 (s, 7 × 3H, CH_3_); ^13^C-NMR (CDCl_3_): 183.8 (COOH), 176.2 (COONC), 156.6 (COONC), 146.4, 143.8, 143.7 (C-13), 122.1(C-12), 118.0, 111.7 (aromatic carbons), 55.7, 47.1, 46.5, 45.8, 41.5, 40.9, 39.3, 38.6, 37.0, 33.7, 33.0, 32.5, 32.3, 30.6, 30.6, 27.6, 27.1, 25.5, 23.5, 23.4, 23.1, 22.8, 19.7, 18.9, 17.0, 15.0 (7 × CH_3_); Anal. Calcd for C_35_H_49_NO_5_: C, 74.57; H, 8.76; N, 2.48. found: C, 74.42; H, 8.91; N, 2.25; HRMS calcd for C_37_H_50_ClNO_4 _(M+H)^+^: 564.36835, found: 564.36804. 

*Benzyl oleanolic acid 3-oxime ester* (**B-01**). 4-Chlorobenzoic acid (0.66 g, 4.2 mmol) and DCC (1 g, 5 mmol) were successively added to a soln. of benzyl oleanate 3-oxime **2 **(2.00 g, 3.5 mmol) which was prepared according to the reported method [13] in CH_2_Cl_2_ (50 mL), Then the reaction mixture was refluxed for 8–14 h at the end of which time TLC (6:1 petroleum ether-EtOAc) indicated that the reaction was complete. The reaction mixture was filtered, the soln was concentrated, and the residue was subjected to column chromatography (8:1 petroleum ether-EtOAc) to give the desired product **B-01** (1.98 g, 80%) as a white foamy solid. ^1^H-NMR (CDCl_3_): *δ* 8.01–7.31 (m, 9H, Ar-H), 5.29 (br s, 1 H, H-12), 5.08 (dd, 2H, *J* = 12.5, 17.4 Hz, Ar-CH_2_), 3.04–2.89 (m, 2H), 2.47–2.33 (m, 1H), 1.34, 1.19, 1.12, 1.03, 0.92, 0.89, 0.64 (s, 7 × 3H, CH_3_); ^13^C-NMR (CDCl_3_): 177.3 (COOBn), 176.4 (COONC), 163.3 (COONC), 143.8 (C-13), 139.4, 134.4, 130.8, 130.8, 128.8, 128.8, 128.4, 128.4, 128.4, 128.1, 127.9, 127.9 (aromatic carbons), 122.1 (C-12), 65.9, 55.7, 47.1, 46.7, 45.8, 41.7, 41.5, 41.4, 39.3, 38.7, 36.9, 33.8, 33.0, 32.3, 32.3, 30.6, 27.5, 27.2, 25.7, 23.6, 23.4, 23.2, 23.0, 19.8, 18.9, 16.8, 15.1 (7 × CH_3_); Anal. Calcd for C_44_H_56_ClNO_4_: C, 75.67; H, 8.08; N, 2.01. found: C, 75.52; H, 8.33; N, 2.17; HRMS calcd for C_37_H_50_ClNO_4 _(M+H)^+^: 698.39706, found: 698.39526. 

*Benzyl oleanolic acid 3-oxime ester* (**B-02**). The reaction was run similarly to that used to synthesize **B-01**. A white foamy solid **B-02** was obtained in 83% yield. ^1^H-NMR (CDCl_3_): *δ* 7.80–7.29 (m, 8H, Ar-H), 5.28 (br s, 1 H, H-12), 5.08 (dd, 2H, *J* = 12.5, 17.4 Hz, Ar-CH_2_), 3.04–2.89 (m, 2H), 2.48–2.33 (m, 1H), 1.32, 1.18, 1.12, 1.01, 0.92, 0.89, 0.64 (s, 7 × 3H, CH_3_); ^13^C-NMR (CDCl_3_): 177.4 (COOBn), 176.6 (COONC), 163.1 (COONC), 143.9 (C-13), 138.2, 136.4, 134.3, 132.4, 130.8, 128.5, 128.4, 128.4, 128.0, 128.0, 127.9, 127.1 (aromatic carbons), 122.1 (C-12), 65.9, 55.9, 47.1, 46.7, 45.8, 41.8, 41.7, 41.4, 39.3, 38.8, 37.0, 33.9, 33.1, 32.4, 32.3, 30.7, 27.6, 27.1, 25.8, 23.6, 23.5, 23.1, 23.0, 20.2, 19.0, 16.8, 15.1 (7 × CH_3_); Anal. Calcd for C_44_H_55_Cl_2_NO_4_: C, 72.11; H, 7.56; N, 1.91. found: C, 72.31; H, 7.49; N, 1.77; HRMS calcd for C_37_H_50_ClNO_4 _(M+H)^+^: 732.35809, found: 732.35529. 

*Benzyl oleanolic acid 3-oxime ester* (**B-03**). The reaction was run similarly to that used to synthesize **B-01**. A white foamy solid **B-03** was obtained in 79% yield. ^1^H-NMR (CDCl_3_): *δ* 8.01–7.32 (m, 9H, Ar-H), 5.30 (br s, 1 H, H-12), 5.08 (dd, 2H, *J* = 12.5, 17.4 Hz, Ar-CH_2_), 3.04–2.88 (m, 2H), 2.48–2.33 (m, 1H), 1.34, 1.19, 1.12, 1.02, 0.92, 0.89, 0.65 (s, 7 × 3H, CH_3_); ^13^C-NMR (CDCl_3_): 177.3 (COOBn), 176.6 (COONC), 163.0 (COONC), 143.8 (C-13), 136.3, 134.5, 133.0, 131.4, 129.8, 129.4, 128.4, 128.4, 127.9, 127.9, 127.9, 127.6 (aromatic carbons), 122.1 (C-12), 65.9, 55.7, 47.1, 46.7, 45.8, 41.7, 41.6, 41.4, 39.3, 38.7, 36.9, 33.8, 33.0, 32.3, 32.3, 30.6, 27.5, 27.1, 25.7, 23.6, 23.4, 23.2, 23.0, 19.9, 18.9, 16.8, 15.1 (7 × CH_3_); Anal. Calcd for C_44_H_56_ClNO_4_: C, 75.67; H, 8.08; N, 2.01. found: C, 75.52; H, 8.27; N, 2.27; HRMS calcd for C_37_H_50_ClNO_4 _(M+H)^+^: 698.39706, found: 698.39697. 

*Benzyl oleanolic acid 3-oxime ester* (**B-04**). The reaction was run similarly to that used to synthesize **B-01**. A white foamy solid **B-04** was obtained in 85% yield. ^1^H-NMR (CDCl_3_): *δ* 8.87–7.29 (m, 9H, Ar-H), 5.31 (br s, 1 H, H-12), 5.08 (dd, 2H, *J* = 12.5, 17.4 Hz, Ar-CH_2_), 3.06–2.90 (m, 2H), 2.50–2.48 (m, 1H), 1.35, 1.21, 1.12, 1.04, 0.92, 0.90, 0.65 (s, 7 × 3H, CH_3_); ^13^C-NMR (CDCl_3_): 177.2 (COOBn), 177.1 (COONC), 162.1 (COONC), 148.2, 143.8 (C-13), 136.3, 135.1, 131.5, 129.7, 128.3, 128.3, 127.9, 127.9, 127.8, 127.3, 124.2 (aromatic carbons), 122.1 (C-12), 65.8, 55.7, 47.1, 46.7, 45.8, 41.7, 41.6, 41.4, 39.3, 38.6, 36.9, 33.8, 33.0, 32.3, 32.3, 30.6, 27.5, 27.2, 25.7, 23.5, 23.4, 23.2, 23.0, 20.0, 18.9, 16.8, 15.0 (7 × CH_3_); Anal. Calcd for C_44_H_56_N_2_O_6_: C, 74.55; H, 7.96; N, 3.95. found: C, 74.40; H, 7.79; N, 3.65; HRMS calcd for C_37_H_50_ClNO_4 _(M+H)^+^: 709.42111, found: 709.42096. 

*Benzyl oleanolic acid 3-oxime ester* (**B-05**). The reaction was run similarly to that used to synthesize **B-01**. A white foamy solid **B-05** was obtained in 75% yield. ^1^H-NMR (CDCl_3_): *δ* 8.04–7.23 (m, 12H, Ar-H), 5.28 (br s, 1 H, H-12), 5.06 (dd, 2H, *J* = 12.5, 17.4 Hz, Ar-CH_2_), 4.19 (s, 2 H, CH_2_C=O), 2.93–2.87 (m, 1H), 2.57–2.50 (m, 1H), 1.19, 1.08, 1.04, 0.92, 0.89, 0.89, 0.60 (s, 7 × 3H, CH_3_); ^13^C-NMR (CDCl_3_): 177.3 (COOBn), 175.6 (COONC), 169.3 (COONC), 143.7 (C-13), 136.3, 133.7, 132.0, 130.2, 128.6, 128.5, 128.3, 127.9, 127.9, 127.8, 127.8, 126.2, 125.7, 125.6, 125.3, 123.8 (aromatic carbons), 122.1 (C-12), 65.8, 55.7, 47.0, 46.6, 45.7, 41.7, 41.3, 41.3, 39.2, 38.5, 38.2, 36.8, 36.8, 33.8, 33.0, 32.2, 30.6, 27.5, 26.9, 25.7, 23.6, 23.3, 22.9, 22.9, 19.3, 18.8, 16.7, 15.0 (7 × CH_3_); Anal. Calcd for C_49_H_61_NO_4_: C, 80.84; H, 8.45; N, 1.92. found: C, 80.69; H, 8.68; N, 1.69; HRMS calcd for C_37_H_50_ClNO_4 _(M+H)^+^: 728.46734, found: 728.46869. 

*Benzyl oleanolic acid 3-oxime ester* (**B-06**). The reaction was run similarly to that used to synthesize **B-01**. A white foamy solid **B-06** was obtained in 72% yield. ^1^H-NMR (CDCl_3_): *δ* 7.36–6.85 (m, 9H, Ar-H), 5.29 (br s, 1 H, H-12), 5.08 (dd, 2H, *J* = 12.5, 17.4 Hz, Ar-CH_2_), 4.82 (s, 2 H, CH_2_C=O), 2.94–2.82 (m, 2H), 2.32–2.26 (m, 1H), 1.23, 1.12, 1.11, 0.98, 0.92, 0.90, 0.63 (s, 7 × 3H, CH_3_); ^13^C-NMR (CDCl_3_): 177.3 (COOBn), 176.0 (COONC), 167.9 (COONC), 156.5, 143.7 (C-13), 136.3, 129.4, 129.4, 128.4, 128.4, 127.9, 127.9, 127.9, 126.6, 122.1 (C-12), 116.1, 116.1 (aromatic carbons), 65.9, 65.4, 55.8, 47.1, 46.7, 45.7, 41.7, 41.5, 41.3, 39.2, 38.6, 36.9, 33.8, 33.0, 32.3, 30.6, 30.6, 27.5, 27.0, 25.7, 23.6, 23.4, 23.0, 23.0, 19.4, 18.9, 16.8, 15.0 (7 × CH_3_); Anal. Calcd for C_45_H_58_ClNO_5_: C, 74.20; H, 8.03; N, 1.92. found: C, 74.06; H, 8.29; N, 1.72; HRMS calcd for C_37_H_50_ClNO_4 _(M+H)^+^: 728.40763, found: 728.40668. 

*Benzyl oleanolic acid 3-oxime ester* (**B-07**). The reaction was run similarly to that used to synthesize **B-01**. A white foamy solid **B-07** was obtained in 75% yield. ^1^H-NMR (CDCl_3_): *δ* 8.80–7.27 (m, 9H, Ar-H), 5.30 (br s, 1 H, H-12), 5.08 (dd, 2H, *J* = 12.5, 17.4 Hz, Ar-CH_2_), 3.04–2.89 (m, 2H), 2.47–2.33 (m, 1H), 1.33, 1.18, 1.11, 1.01, 0.92, 0.89, 0.64 (s, 7 × 3H, CH_3_); ^13^C-NMR (CDCl_3_): 177.0 (COOBn), 176.0 (COONC), 162.2 (COONC), 143.5 (C-13), 136.2, 132.6, 132.1, 128.2, 128.2, 128.2, 127.7, 127.7, 127.7, 123.9, 121.9 (C-12), 116.8, 116.5 (aromatic carbons), 65.6, 55.6, 46.9, 46.4, 45.5, 41.5, 41.2, 41.2, 39.0, 38.5, 36.7, 33.6, 32.9, 32.1, 32.1, 30.4, 27.3, 26.9, 25.5, 23.4, 23.2, 22.9, 22.8, 19.8, 18.7, 16.6, 14.8 (7 × CH_3_); Anal. Calcd for C_44_H_56_FNO_4_: C, 77.50; H, 8.28; N, 2.05. found: C, 77.41; H, 8.41; N, 2.25; HRMS calcd for C_37_H_50_ClNO_4 _(M+H)^+^: 682.42661, found: 682.42645. 

*Benzyl oleanolic acid 3-oxime ester* (**B-08**). The reaction was run similarly to that used to synthesize **B-01**. A white foamy solid **B-08** was obtained in 78% yield. ^1^H-NMR (CDCl_3_): *δ* 7.93–7.26 (m, 9H, Ar-H), 5.29 (br s, 1 H, H-12), 5.08 (dd, 2H, *J* = 12.5, 17.4 Hz, Ar-CH_2_), 3.04–2.89 (m, 2H), 2.44–2.33 (m, 1H), 1.33, 1.19, 1.11, 1.02, 0.91, 0.89, 0.64 (s, 7 × 3H, CH_3_); ^13^C-NMR (CDCl_3_): 177.2 (COOBn), 176.3 (COONC), 163.4 (COONC), 143.8 (C-13), 136.3, 131.7, 131.7, 130.9, 130.9, 128.6, 128.3, 128.3, 128.0, 127.9, 127.9, 127.8 (aromatic carbons), 122.1 (C-12), 65.8, 55.7, 47.0, 46.6, 45.7, 41.7, 41.5, 41.4, 39.3, 38.6, 36.9, 33.7, 33.0, 32.3, 32.3, 30.6, 27.5, 27.2, 25.7, 23.5, 23.4, 23.2, 23.0, 19.8, 18.9, 16.8, 15.0 (7 × CH_3_); Anal. Calcd for C_44_H_56_BrNO_4_: C, 71.14; H, 7.60; N, 1.89. found: C, 71.35; H, 7.53; N, 1.60; HRMS calcd for C_37_H_50_ClNO_4 _(M+H)^+^: 742.34655, found: 742.34674. 

*Benzyl oleanolic acid 3-oxime ester* (**B-09**). The reaction was run similarly to that used to synthesize **B-01**. A white foamy solid **B-09** was obtained in 81% yield. ^1^H-NMR (CDCl_3_): *δ* 9.25–7.28 (m, 8H, Ar-H), 5.30 (br s, 1 H, H-12), 5.08 (dd, 2H, *J* = 12.5, 17.4 Hz, Ar-CH_2_), 3.06–2.89(m, 2H), 2.48–2.33 (m, 1H), 1.34, 1.20, 1.12, 1.03, 0.92, 0.90, 0.65 (s, 7 × 3H, CH_3_); ^13^C-NMR (CDCl_3_): 177.2 (COOBn), 176.7 (COONC), 162.8 (COONC), 153.3, 150.5, 143.7 (C-13), 136.9, 136.3, 128.3, 128.3, 127.9, 127.9, 127.8, 125.7, 123.4 (aromatic carbons), 122.0 (C-12), 65.8, 55.7, 47.0, 46.6, 45.7, 41.7, 41.6, 41.4, 39.2, 38.6, 36.9, 33.8, 33.0, 32.3, 32.2, 30.6, 27.5, 27.1, 25.7, 23.5, 23.4, 23.1, 23.0, 19.9, 18.9, 16.8, 15.0 (7 × CH_3_); Anal. Calcd for C_43_H_56_N_2_O_4_: C, 77.67; H, 8.49; N, 4.21. found: C, 77.73; H, 8.62; N, 4.03; HRMS calcd for C_37_H_50_ClNO_4 _(M+H)^+^: 665.43128, found: 665.43182. 

*Benzyl oleanolic acid 3-oxime ester* (**B-10**). The reaction was run similarly to that used to synthesize **B-01**. A white foamy solid **B-10** was obtained in 71% yield. ^1^H-NMR (CDCl_3_): *δ* 7.60–6.51(m, 8H, Ar-H), 5.29 (br s, 1 H, H-12), 5.08 (dd, 2H, *J* = 12.5, 17.4 Hz, Ar-CH_2_), 3.06–2.88 (m, 2H), 2.43–2.33 (m, 1H), 1.32, 1.25, 1.11, 1.02, 0.92, 0.89, 0.64 (s, 7 × 3H, CH_3_); ^13^C-NMR (CDCl_3_): 177.3 (COOBn), 176.3 (COONC), 156.6 (COONC), 146.4, 143.8, 143.7 (C-13), 136.4, 128.4, 128.4, 128.0, 128.0, 127.9, 122.1 (C-12), 118.0, 111.8 (aromatic carbons), 65.9, 55.7, 47.1, 46.7, 45.8, 41.7, 41.5, 41.4, 39.3, 38.7, 36.9, 33.8, 33.0, 32.3, 32.3, 30.6, 27.5, 27.2, 25.7, 23.6, 23.4, 23.2, 23.0, 19.8, 18.9, 16.8, 15.1 (7 × CH_3_); Anal. Calcd for C_42_H_55_NO_5_: C, 77.15; H, 8.48; N, 2.14. found: C, 77.01; H, 8.30; N, 2.27; HRMS calcd for C_37_H_50_ClNO_4 _(M+H)^+^: 654.41530, found: 654.41504. 

*Benzyl oleanolic acid 3-oxime ester*(**B-11**). The reaction was run similarly to that used to synthesize **B-01**. A white foamy solid **B-11** was obtained in 84% yield. ^1^H-NMR (CDCl_3_): *δ* 8.86–7.28 (m, 9H, Ar-H), 5.30 (br s, 1 H, H-12), 5.08 (dd, 2H, *J* = 12.5, 17.4 Hz, Ar-CH_2_), 3.06–2.90 (m, 2H), 2.50–2.48 (m, 1H), 1.35, 1.21, 1.12, 1.04, 0.92, 0.90, 0.65 (s, 7 × 3H, CH_3_); ^13^C-NMR (CDCl_3_): 177.2 (COOBn), 177.1 (COONC), 162.1 (COONC), 148.2, 143.7 (C-13), 136.3, 135.1, 131.4, 129.7, 128.3, 128.3, 127.9, 127.9, 127.8, 127.3, 124.2 (aromatic carbons), 122.0 (C-12), 65.8, 55.7, 47.0, 46.6, 45.7, 41.7, 41.6, 41.4, 39.2, 38.6, 36.9, 33.8, 33.0, 32.3, 32.2, 30.6, 27.5, 27.2, 25.7, 23.4, 23.4, 23.2, 22.9, 20.0, 19.0, 16.7, 15.0 (7 × CH_3_); Anal. Calcd for C_44_H_56_N_2_O_6_: C, 74.55; H, 7.96; N, 3.95. found: C, 74.30; H, 7.88; N, 3.68; HRMS calcd for C_37_H_50_ClNO_4 _(M+H)^+^: 709.42111, found: 709.42267. 

*Benzyl oleanolic acid 3-oxime ester* (**B-12**). The reaction was run similarly to that used to synthesize **B-01**. A white foamy solid **B-12** was obtained in 90% yield. ^1^H-NMR (CDCl_3_): *δ* 9.24–7.28 (m, 8H, Ar-H), 5.30 (br s, 1 H, H-12), 5.08 (dd, 2H, *J* = 12.5, 17.4 Hz, Ar-CH_2_), 3.04–2.88 (m, 2H), 2.54–2.51 (m, 1H), 1.35, 1.22, 1.13, 1.05, 0.92, 0.90, 0.65 (s, 7 × 3H, CH_3_); ^13^C-NMR (CDCl_3_): 178.0 (COOBn), 177.3 (COONC), 160.3 (COONC), 148.7, 143.8 (C-13), 136.3, 133.4, 129.2, 129.2, 128.4, 128.4, 128.0, 128.0, 127.9, 127.9, 122.3 (aromatic carbons), 122.0 (C-12), 65.9, 55.7, 47.1, 46.7, 45.8, 41.9, 41.7, 41.4, 39.3, 38.6, 37.0, 33.8, 33.0, 32.3, 32.3, 30.6, 27.5, 27.1, 25.7, 23.6, 23.4, 23.2, 23.0, 20.2, 18.9, 16.8, 15.1 (7 × CH_3_); Anal. Calcd for C_44_H_55_N_3_O_8_: C, 70.10; H, 7.35; N, 5.57. found: C, 70.43; H, 7.31; N, 5.85; HRMS calcd for C_37_H_50_ClNO_4 _(M+H)^+^: 754.40619, found: 754.40375. 

*Benzyl oleanolic acid 3-oxime ester* (**B-13**). The reaction was run similarly to that used to synthesize **B-01**. A white foamy solid **B-13** was obtained in 76% yield. ^1^H-NMR (CDCl_3_): *δ* 7.82–7.30 (m, 9H, Ar-H), 5.29 (br s, 1 H, H-12), 5.08 (dd, 2H, *J* = 12.5, 17.4 Hz, Ar-CH_2_), 3.07–2.88 (m, 2H), 2.42–2.32 (m, 1H), 1.32, 1.18, 1.12, 1.01, 0.92, 0.89, 0.64 (s, 7 × 3H, CH_3_); ^13^C-NMR (CDCl_3_): 177.3 (COOBn), 176.4 (COONC), 163.9 (COONC), 143.8 (C-13), 136.3, 133.1, 132.3, 131.3, 130.8, 130.2, 128.4, 128.4, 127.9, 127.9, 127.9, 126.6 (aromatic carbons), 122.1 (C-12), 65.9, 55.8, 47.1, 46.7, 45.7, 41.7, 41.6, 41.4, 39.2, 38.8, 36.9, 33.8, 33.0, 32.3, 32.3, 30.6, 27.5, 27.0, 25.7, 23.6, 23.4, 23.1, 23.0, 20.1, 18.9, 16.8, 15.1 (7 × CH_3_); Anal. Calcd for C_44_H_56_ClNO_4_: C, 75.67; H, 8.08; N, 2.01. found: C, 75.55; H, 8.12; N, 2.32; HRMS calcd for C_37_H_50_ClNO_4 _(M+H)^+^: 698.39706, found: 698.39709. 

*Benzyl oleanolic acid 3-oxime ester* (**B-14**). The reaction was run similarly to that used to synthesize **B-01**. A white foamy solid **B-14** was obtained in 79% yield. ^1^H-NMR (CDCl_3_): *δ* 8.80–7.27 (m, 9H, Ar-H), 5.30 (br s, 1 H, H-12), 5.08 (dd, 2H, *J* = 12.5, 17.4 Hz, Ar-CH_2_), 3.04–2.89 (m, 2H), 2.47–2.33 (m, 1H), 1.34, 1.20, 1.12, 1.03, 0.92, 0.90, 0.64 (s, 7 × 3H, CH_3_); ^13^C-NMR (CDCl_3_): 177.1 (COOBn), 176.9 (COONC), 162.5 (COONC), 150.4, 150.4, 143.7 (C-13), 136.8, 136.2, 128.2, 128.2, 128.2, 127.8, 127.8, 127.7, 122.6 (aromatic carbons), 121.9 (C-12), 65.7, 55.6, 46.9, 46.5, 45.6, 41.6, 41.5, 41.2, 39.1, 38.5, 36.8, 33.6, 32.9, 32.1, 32.1, 30.5, 27.4, 27.0, 25.6, 23.4, 23.2, 23.0, 22.8, 19.8, 18.8, 16.6, 14.9 (7 × CH_3_); Anal. Calcd for C_43_H_56_N_2_O_4_: C, 77.67; H, 8.49; N, 4.21. found: C, 77.35; H, 8.53; N, 4.02; HRMS calcd for C_37_H_50_ClNO_4 _(M+H)^+^: 665.43128, found: 665.43146. 

*Benzyl oleanolic acid 3-oxime ester* (**B-15**). The reaction was run similarly to that used to synthesize **B-01**. A white foamy solid **B-15** was obtained in 70% yield. ^1^H-NMR (CDCl_3_): *δ* 8.07–7.32 (m, 10H, Ar-H), 5.30 (br s, 1 H, H-12), 5.08 (dd, 2H, *J* = 12.5, 17.4 Hz, Ar-CH_2_), 3.08–2.88 (m, 2H), 2.47–2.31 (m, 1H), 1.35, 1.19, 1.12, 1.03, 0.92, 0.89, 0.65 (s, 7 × 3H, CH_3_); ^13^C-NMR (CDCl_3_): 177.3 (COOBn), 176.2 (COONC), 164.2 (COONC), 143.8 (C-13), 136.3, 132.9, 129.7, 129.4, 129.4, 128.4, 128.4, 128.4, 128.4, 127.9, 127.9, 127.9 (aromatic carbons), 122.1 (C-12), 65.9, 55.7, 47.1, 46.7, 45.7, 41.7, 41.5, 41.4, 39.3, 38.7, 36.9, 33.8, 33.0, 32.3, 32.3, 30.6, 27.5, 27.1, 25.7, 23.6, 23.4, 23.2, 23.0, 19.8, 18.9, 16.8, 15.1 (7 × CH_3_); Anal. Calcd for C_44_H_57_NO_4_: C, 79.60; H, 8.65; N, 2.11. found: C, 79.55; H, 8.83; N, 2.38; HRMS calcd for C_37_H_50_ClNO_4 _(M+H)^+^: 664.43606, found: 664.43500. 

### 3.3. Enzyme Inhibitory Activities Bioassay

Inhibitory activity of all the synthesized compounds towards *Candida albicans* GlcN-6-P synthase was determined. The *Candida albicans* GFA1 gene encoding the enzyme was PCR amplified and cloned to a yeast expression vector pYES2.0, then induced expression using glactose in *Saccharomyces cerevisiae*. We used the further optimized Elson-Morgan method [14,15,16,17] to determine the activity of the enzyme from pyrophosphate extract in the presence of the synthesized compounds.

Assays were performed in potassium phosphate buffer (0.1M, pH7.0). Incubation mixture (0.4 mL volume) consisted of 15 mM D-Fru-6-P, 10 mM L-glutamine, 1 mM EDTA, 0.35 mM compounds. Following preincubation at 37 °C for 10 min, the enzymatic reaction was initiated by the addition of 0.02 unit of GlmS. The mixture was incubated at 37 °C for 30 min. Enzymatic reaction was terminated by boiling for 1 min. Aliquots of 0.2 mL of saturated NaHCO_3_ solution and 0.1 mL acetic anhydride/acetone mixture (10%v/v, prepared freshly before use) were added and the mixture was incubated at room temperature for 3 min. The acetylation was stopped by boiling for 3 min, followed by cooling on ice. An aliquot of 0.2 mL of 0.8 M K_2_B_4_O_7_ solution, pH 9.2, was added, the mixture was incubated at 100 °C for 3 min and cooled on ice. A 5 mL portion of the Elson-Morgan reagent (1 g of 4-dimethylaminobenzaldehyde dissolved in 100 mL of glacial acetic acid, containing 1.25 mL of concentrated HCl) was added and the resulting mixture was incubated for 20 min at 37 °C. Three replicates were performed. Absorbance at λ = 585 nm was measured and GlcN-6-P concentration in the sample was read from the standard curve [solutions of glucosamine-HCl (0.1–1 mM) were assayed simultaneously, to obtain a standard line from the plot of extinction against concentration of glucosamine]. In each experiment, two control samples, one without enzyme and one without substrates, were assayed in the same way. 

### 3.4. Fungicidal Activity Bioassay

The mycelium growth rate test was used [18]. The culture media, with known concentration of the test compounds, were obtained by mixing the soln of compounds **A**–**B** in methanol with potato dextrose agar (PDA), on which fungus cakes were placed. The blank test was made using methanol. The culture was carried out at 24 ± 0.5 °C. Three replicates were performed.

## 4. Conclusions

Twenty five oleanolic acid 3-oxime esters were designed and efficiently synthesized. The bioassays showed that they had inhibitory activities against glucosamine-6-phosphate synthase, and at the same time, they also exhibited some fungicidal activity against six tested fungi. Although the enzyme inhibitory activities of the target compound are not very obvious compared with the parent compound (**OA**), they exhibited much more fungicidal activity than the latter. All the compounds exhibited better fungicidal activity against *R. solani* and *S. sclerotiorum*. Further studies are in progress.

## Supplementary Materials

Supplementary materials can be accessed at: http://www.mdpi.com/1420-3049/18/3/3615/s1.
