# Supplementary material for: Efficient Synthesis and Anti-Fungal Activity of Oleanolic Acid Oxime Esters
Source: Molecules. 2013 Mar 21;18(3):3615–29. doi: 10.3390/molecules18033615 (PMC6270060; doi:10.3390/molecules18033615)

Supporting Materials

Figure S1. ^{1}H-NMR spectrum of compound A-01.

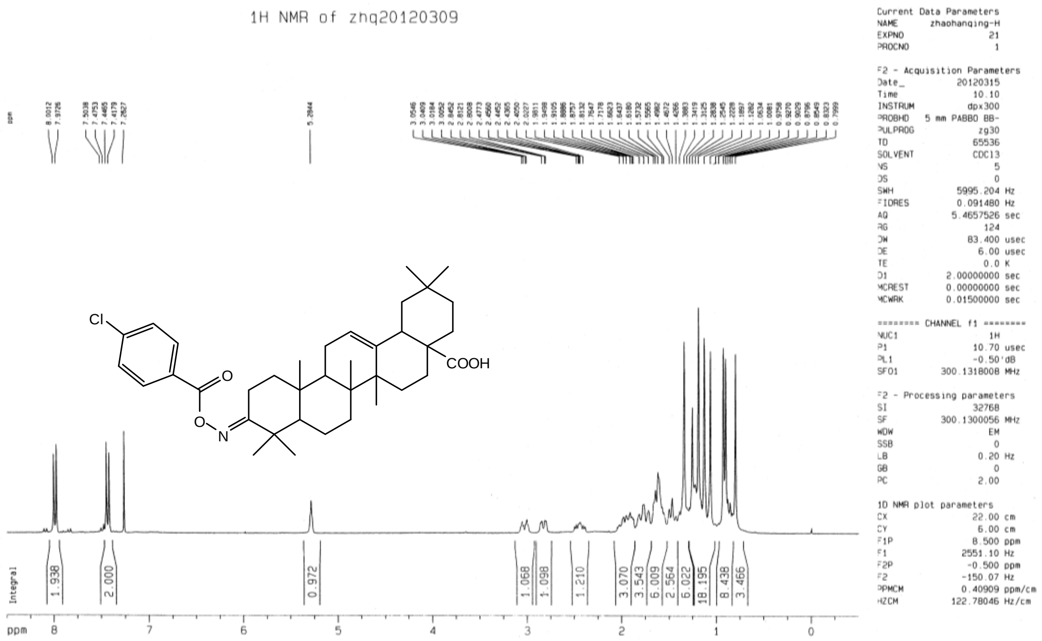

Figure S2. ^{13}C-NMR spectrum of compound A-01.

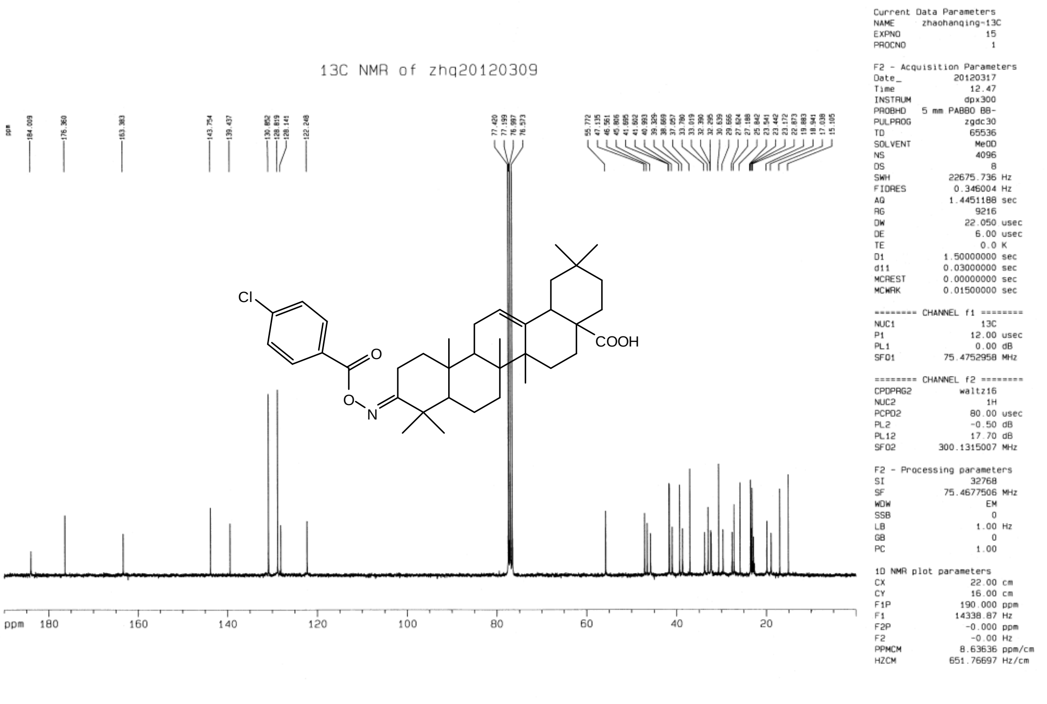

Figure S3. HRMS of compound A-01.

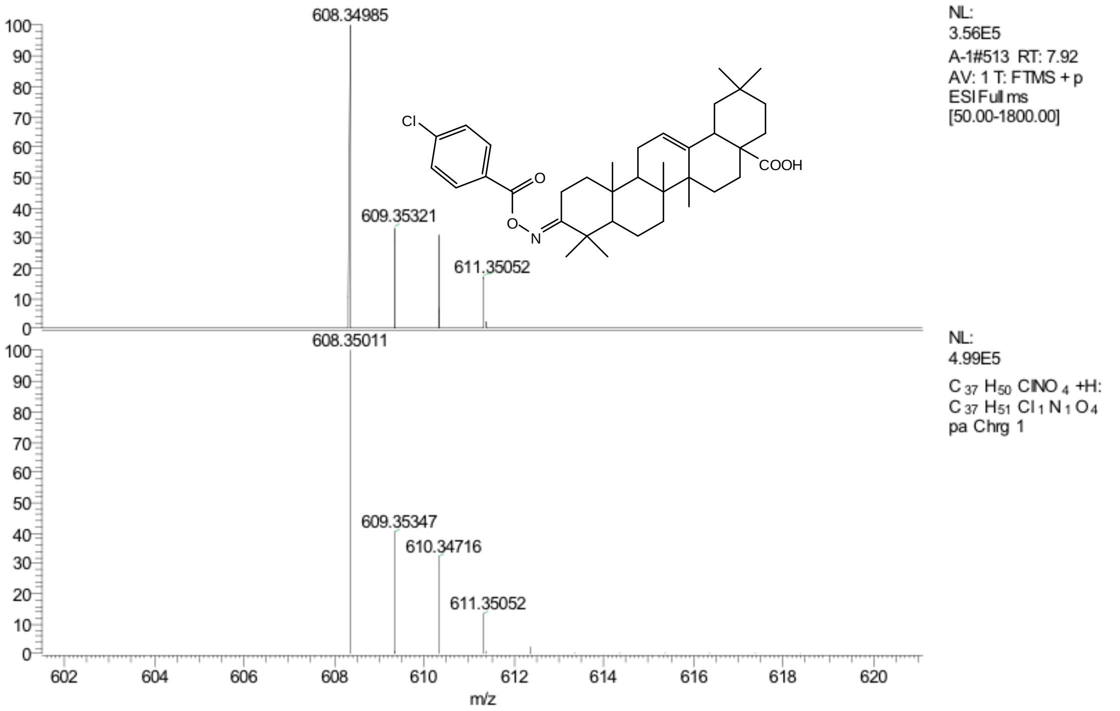

Figure S4. ^{1}H-NMR spectrum of compound A-02.

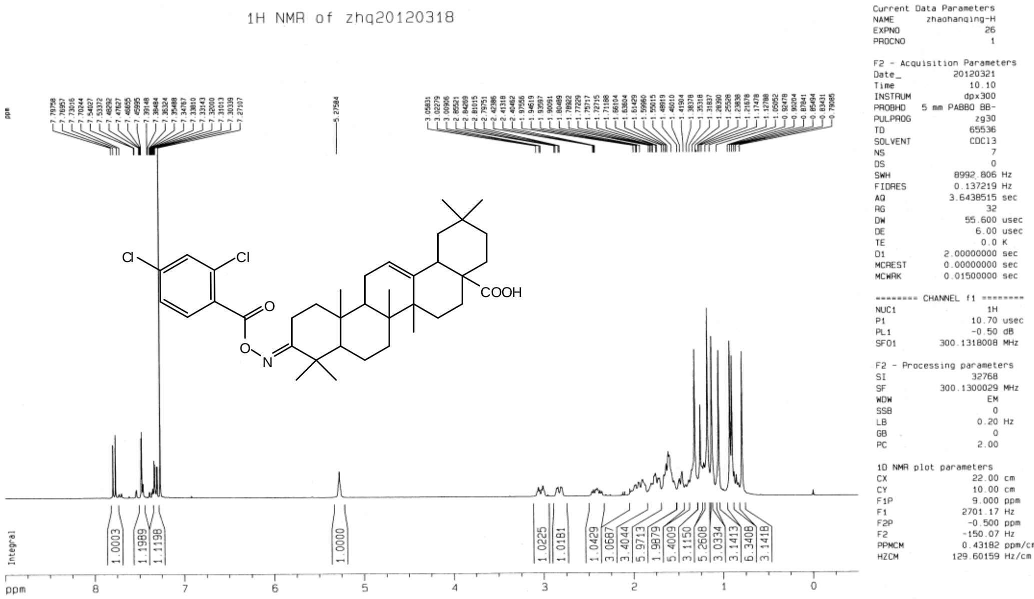

Figure S5. ^{13}C-NMR spectrum of compound A-02.

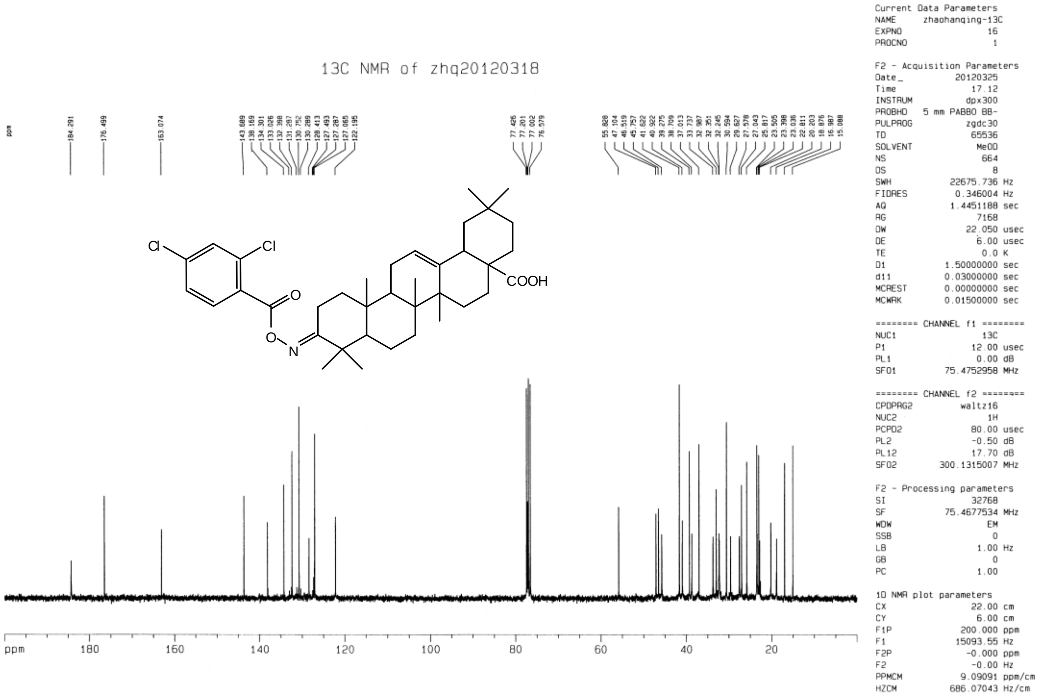

Figure S6. HRMS of compound A-02.

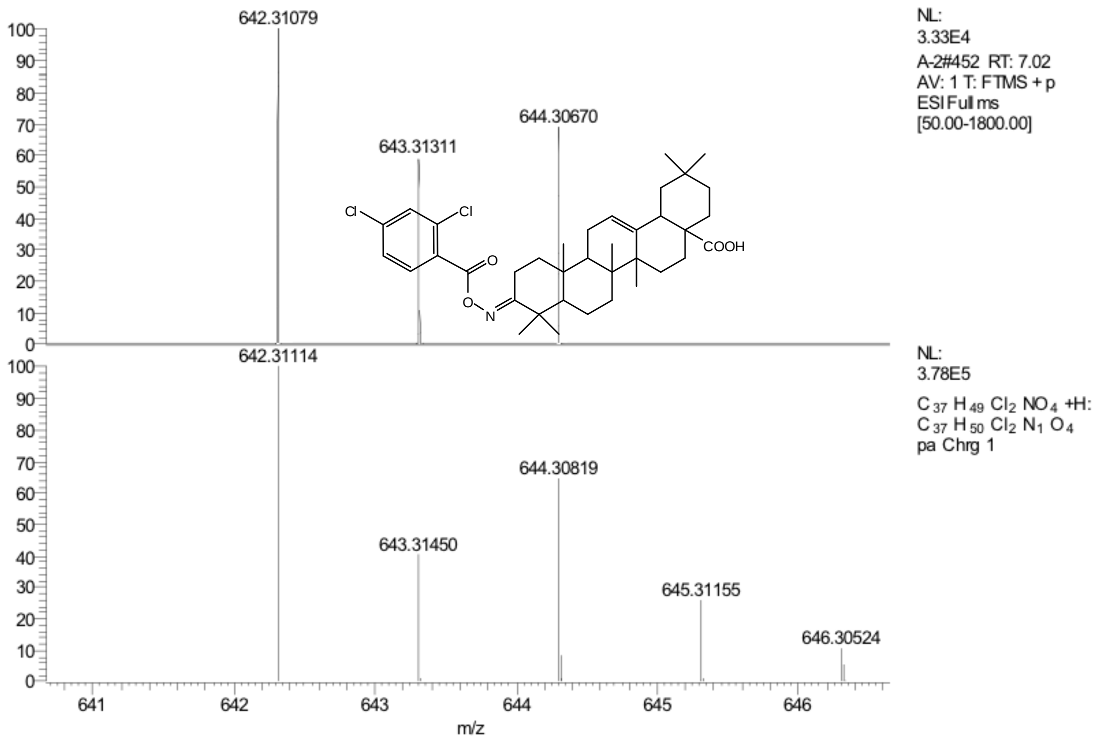

Figure S7. ^{1}H-NMR spectrum of compound A-03.

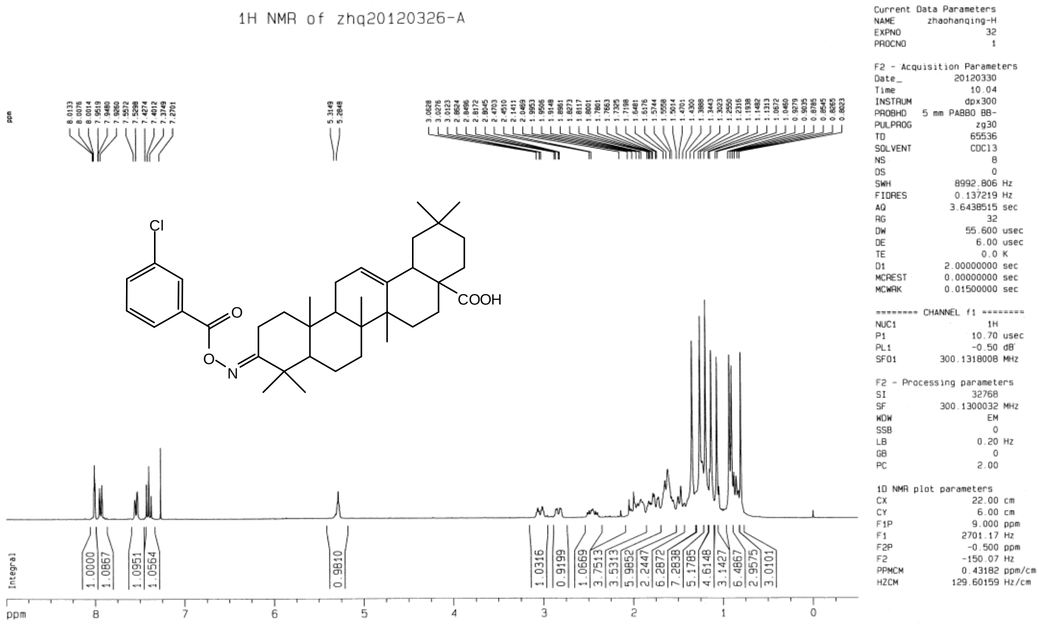

Figure S8. ^{13}C-NMR spectrum of compound A-03.

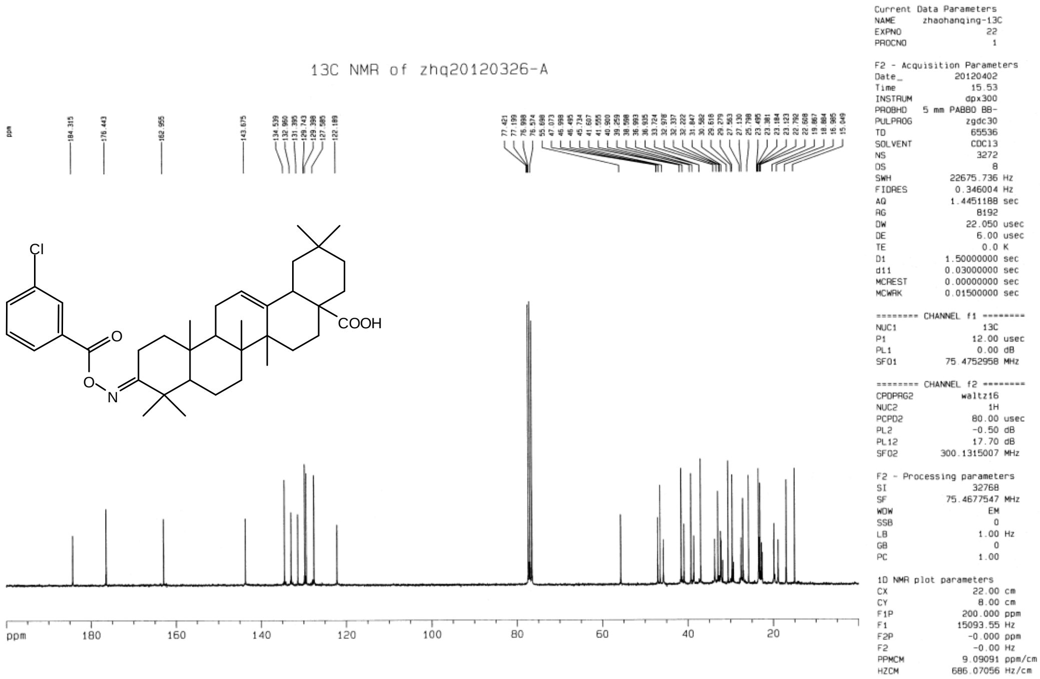

Figure S9. HRMS of compound A-03.

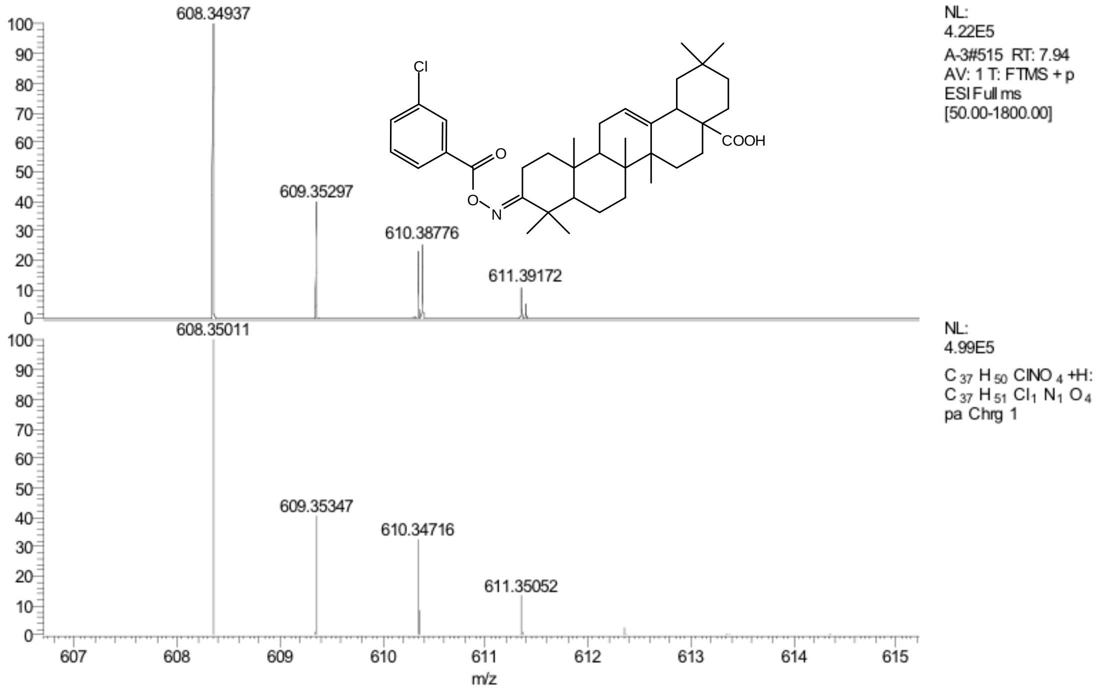

Figure S10. ^{1}H-NMR spectrum of compound A-04.

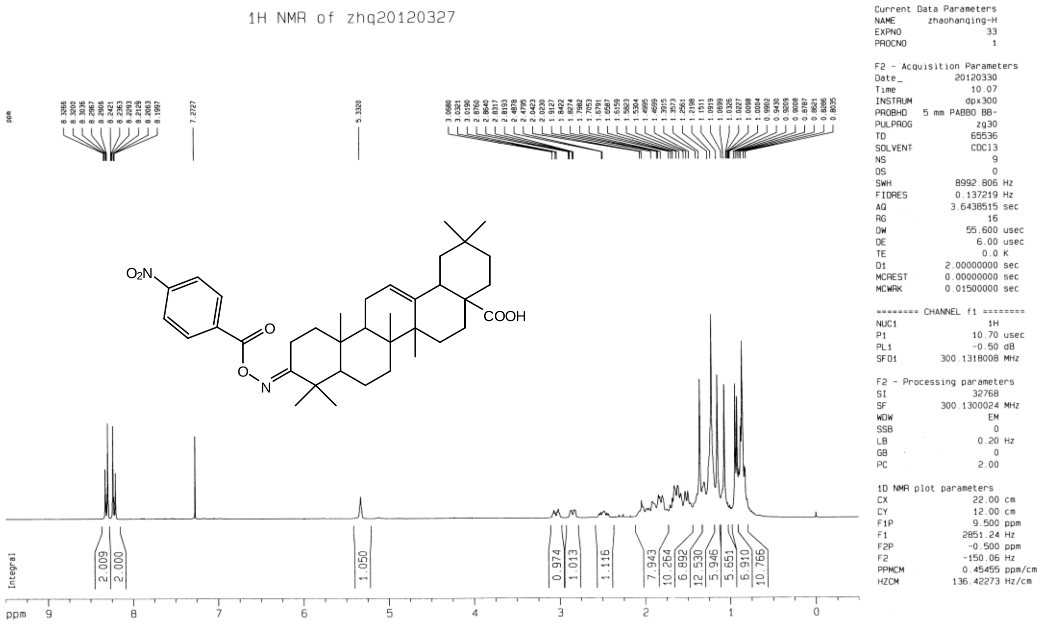

Figure S11. ^{13}C-NMR spectrum of compound A-04.

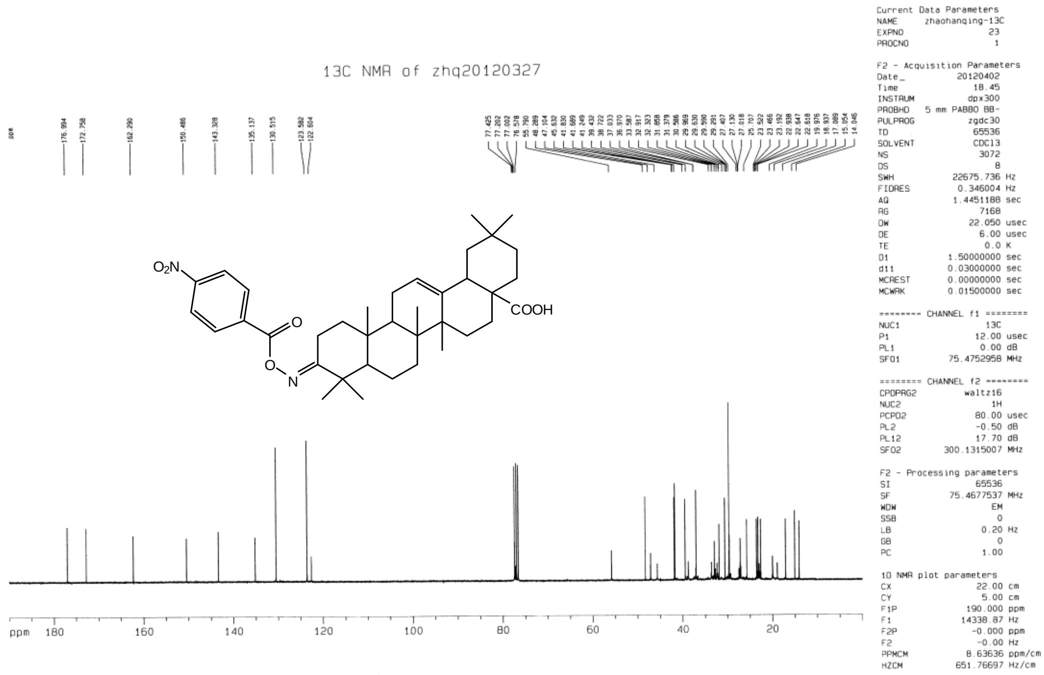

Figure S12. HRMS of compound A-04.

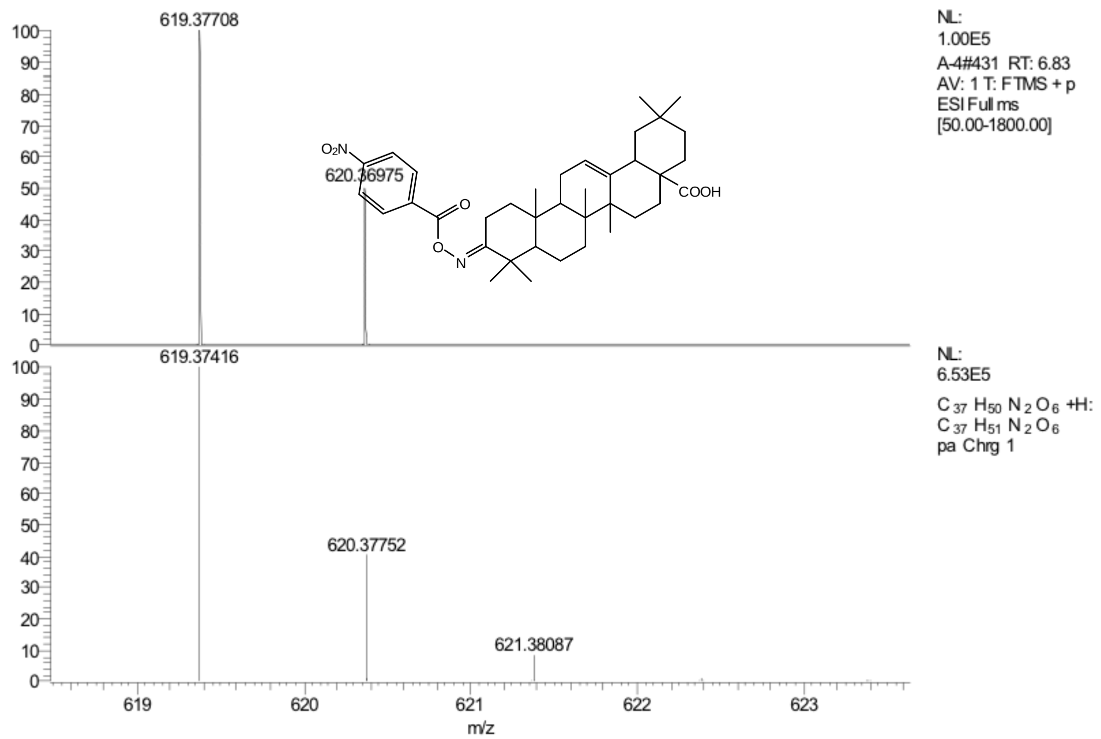

Figure S13. ^{1}H-NMR spectrum of compound A-05.

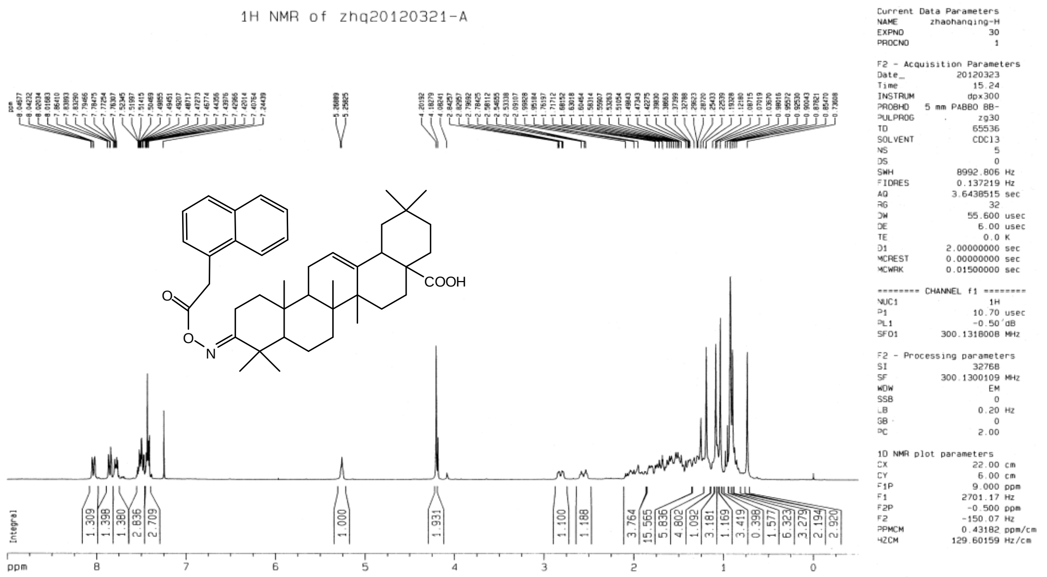

Figure S14. ^{13}C-NMR spectrum of compound A-05.

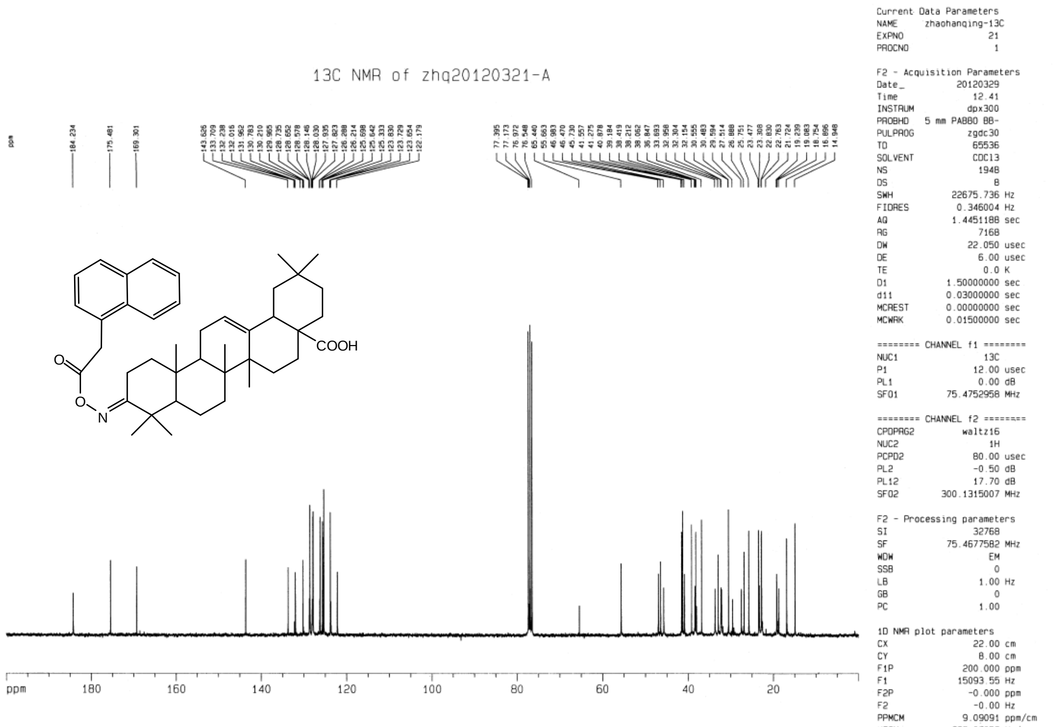

Figure S15. HRMS of compound A-05.

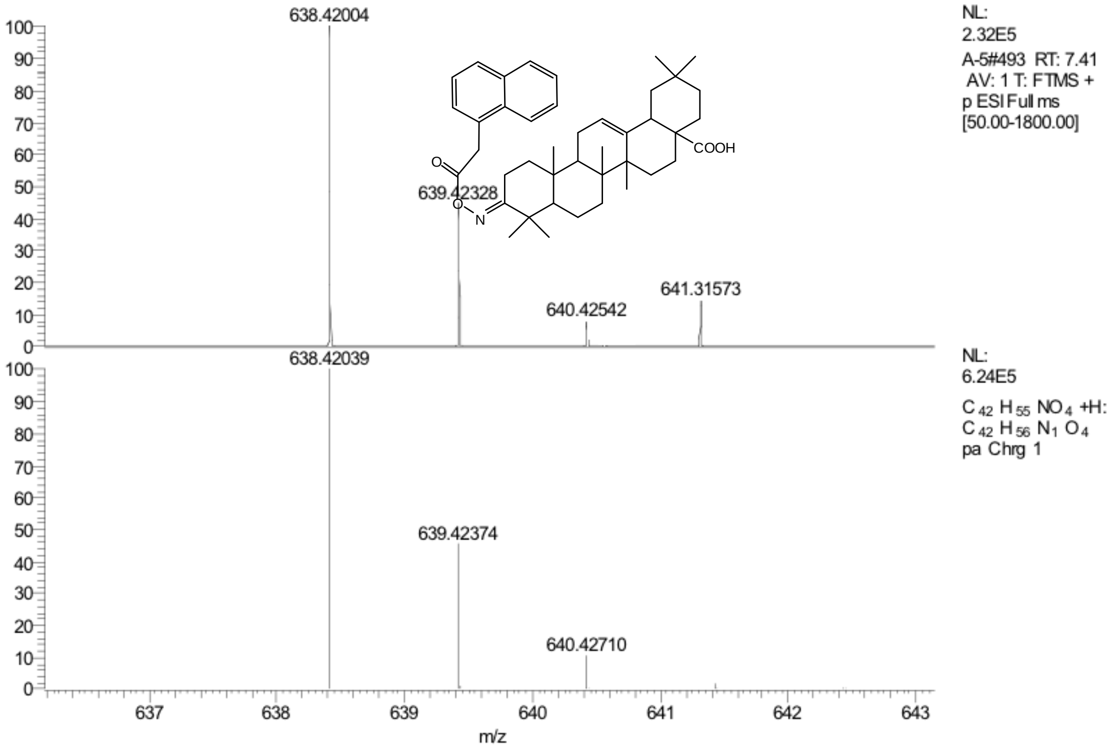

Figure S16. ^{1}H-NMR spectrum of compound A-06.

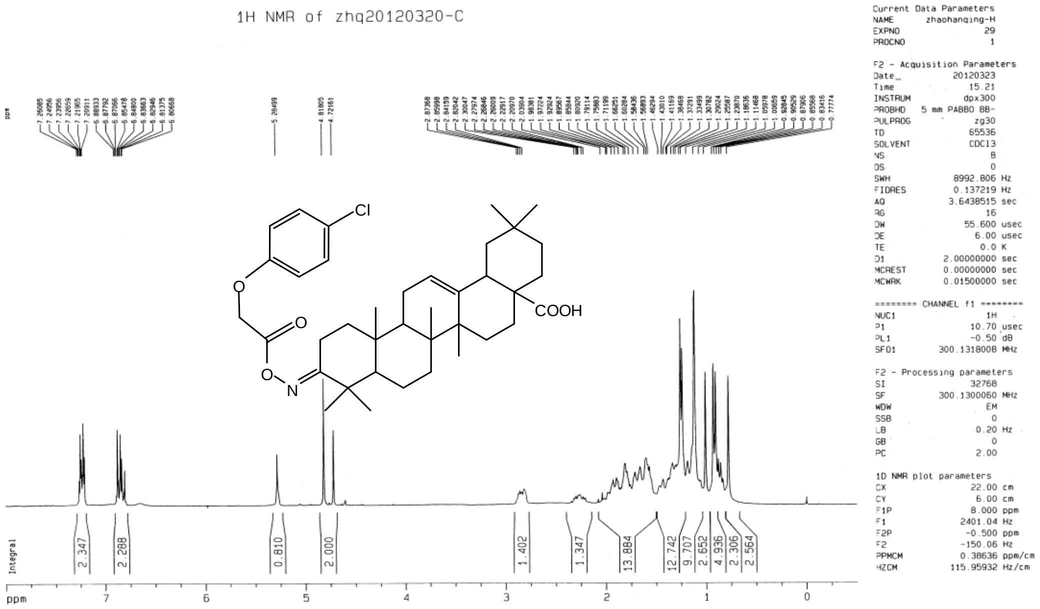

Figure S17. ^{13}C-NMR spectrum of compound A-06.

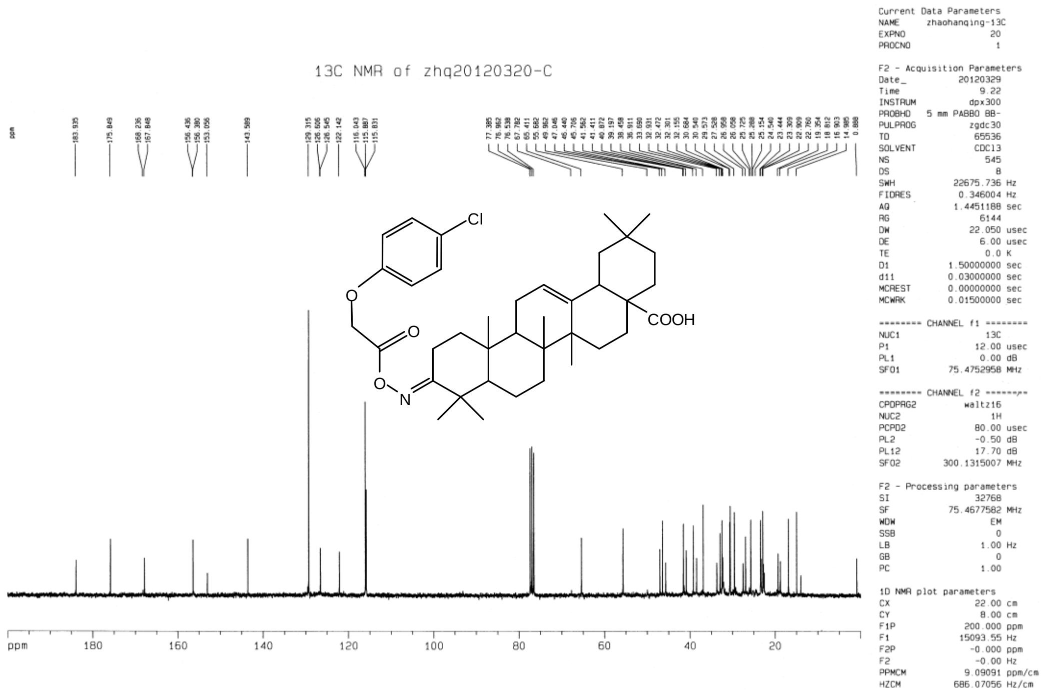

Figure S18. HRMS of compound A-06.

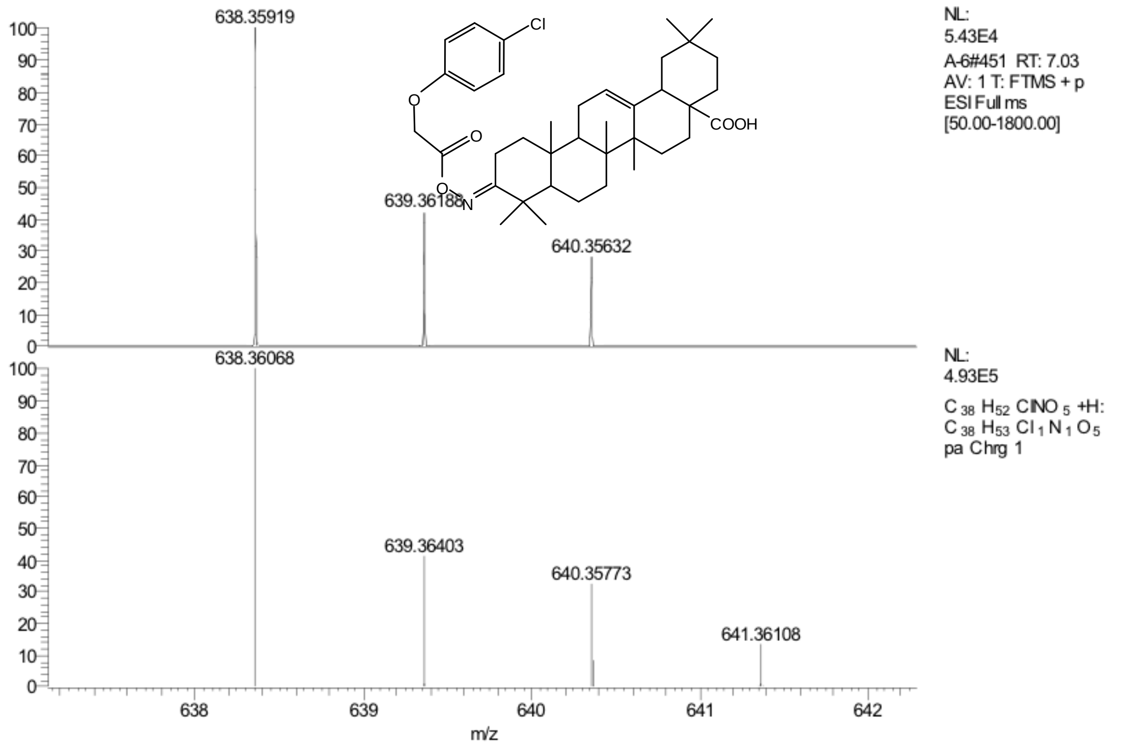

Figure S19. ^{1}H-NMR spectrum of compound A-07.

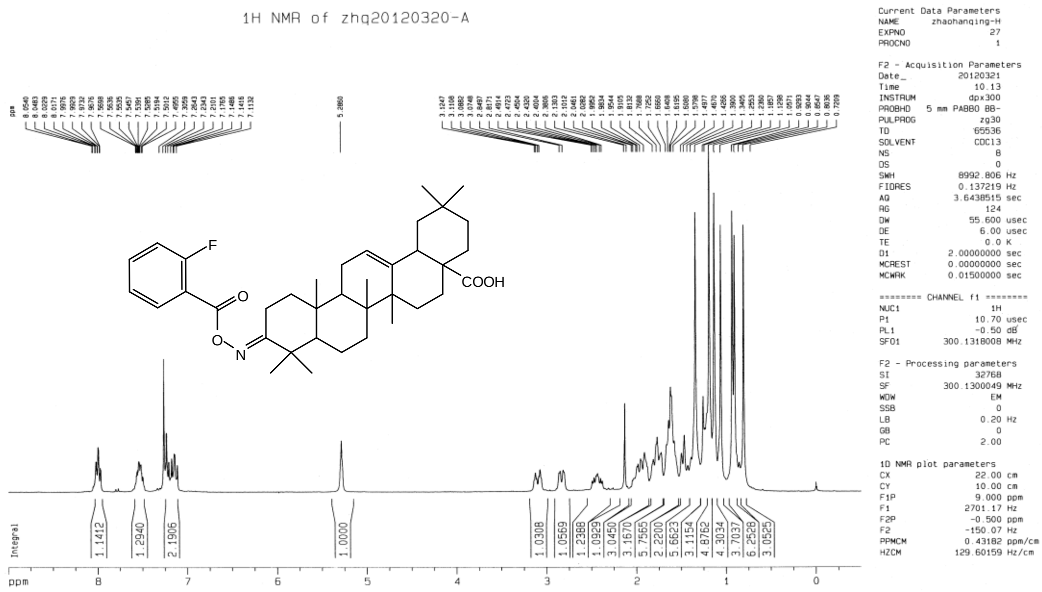

Figure S20. ^{13}C-NMR spectrum of compound A-07.

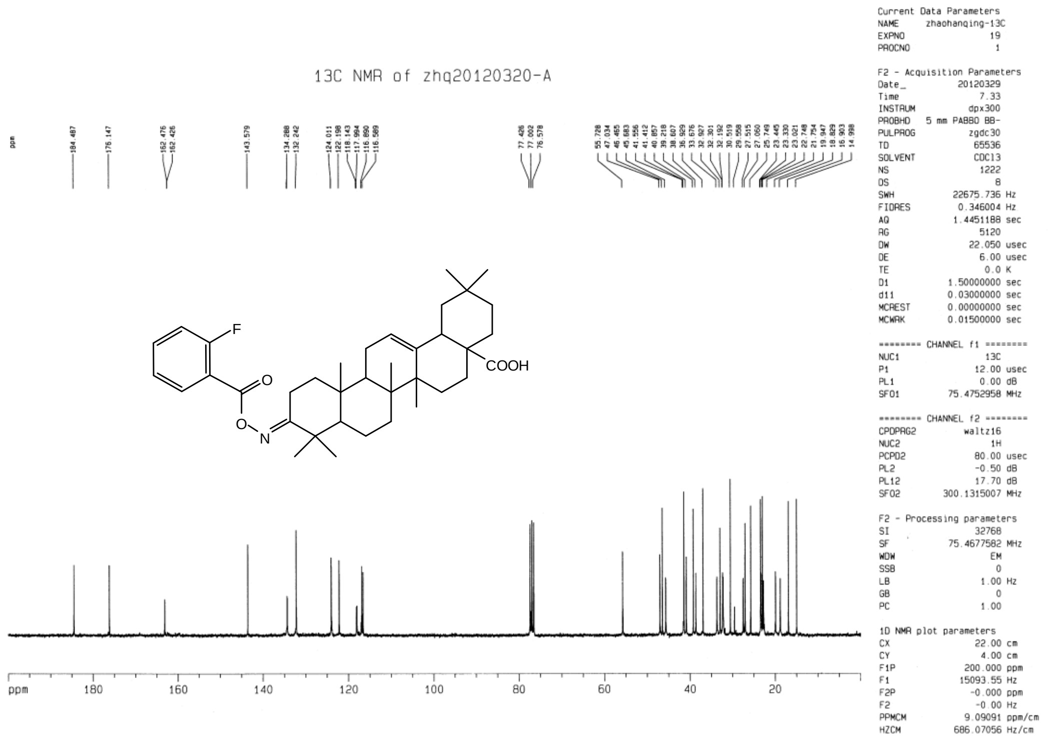

Figure S21. HRMS of compound A-07.

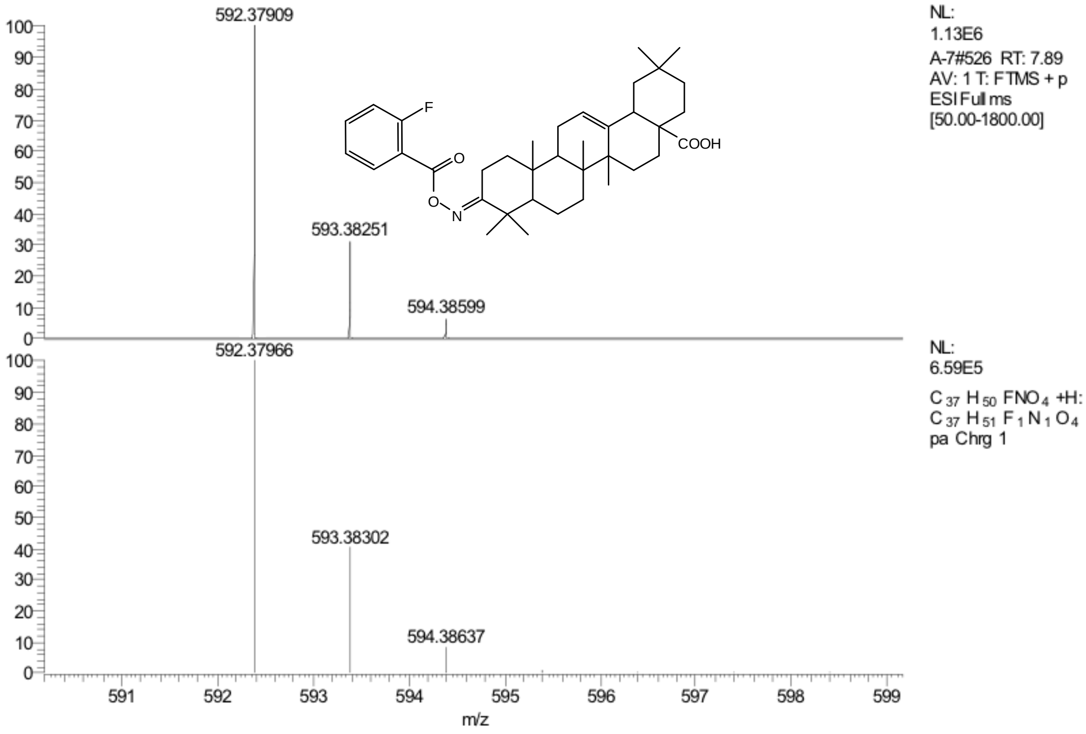

Figure S22. ^{1}H-NMR spectrum of compound A-08.

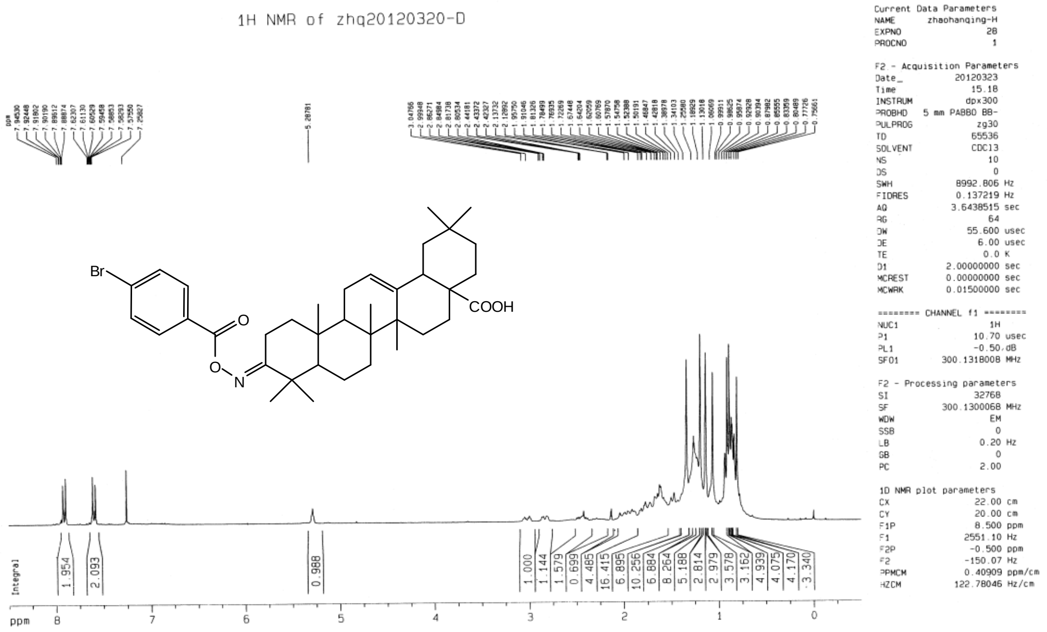

Figure S23. ^{13}C-NMR spectrum of compound A-08.

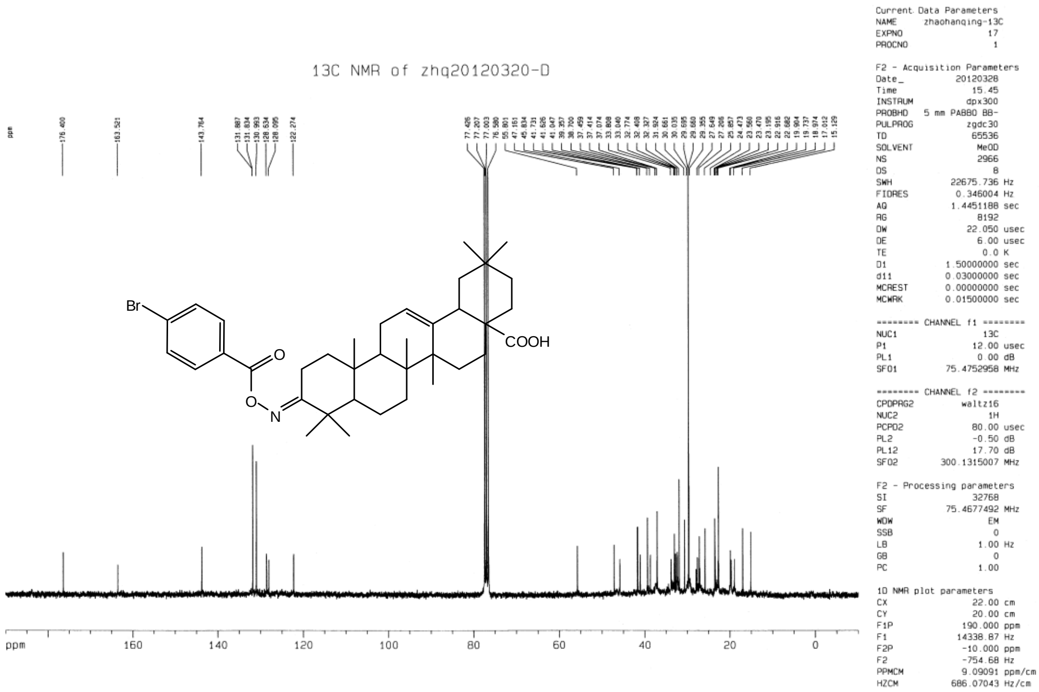

Figure S24. HRMS of compound A-08.

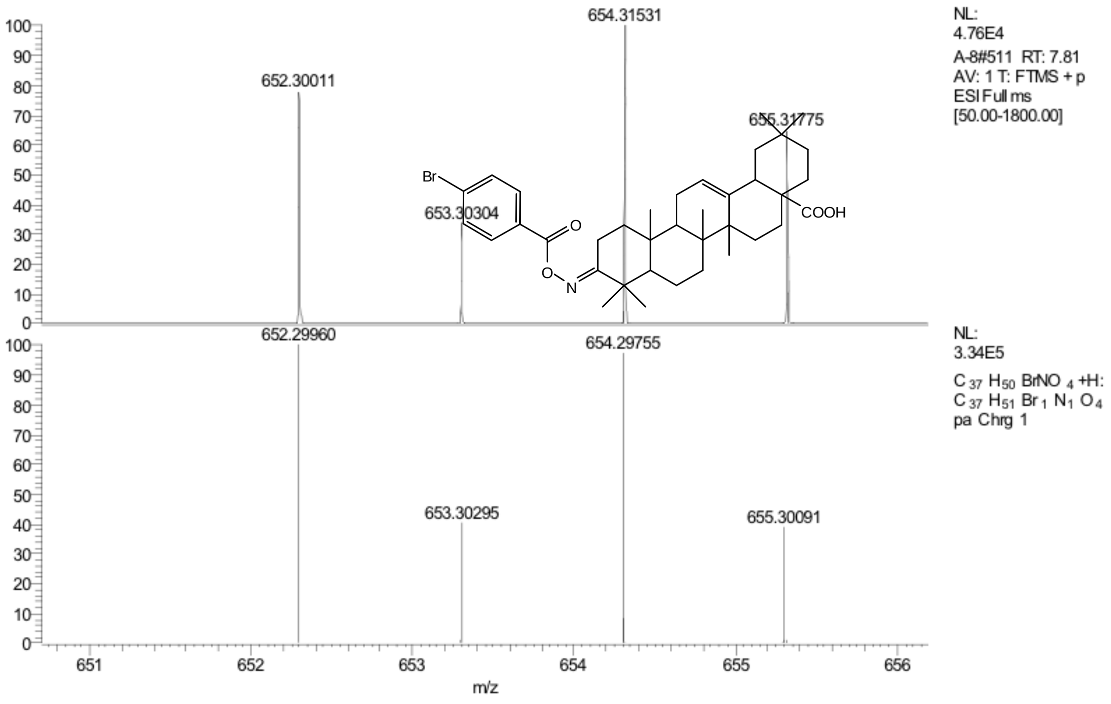

Figure S25. ^{1}H-NMR spectrum of compound A-09.

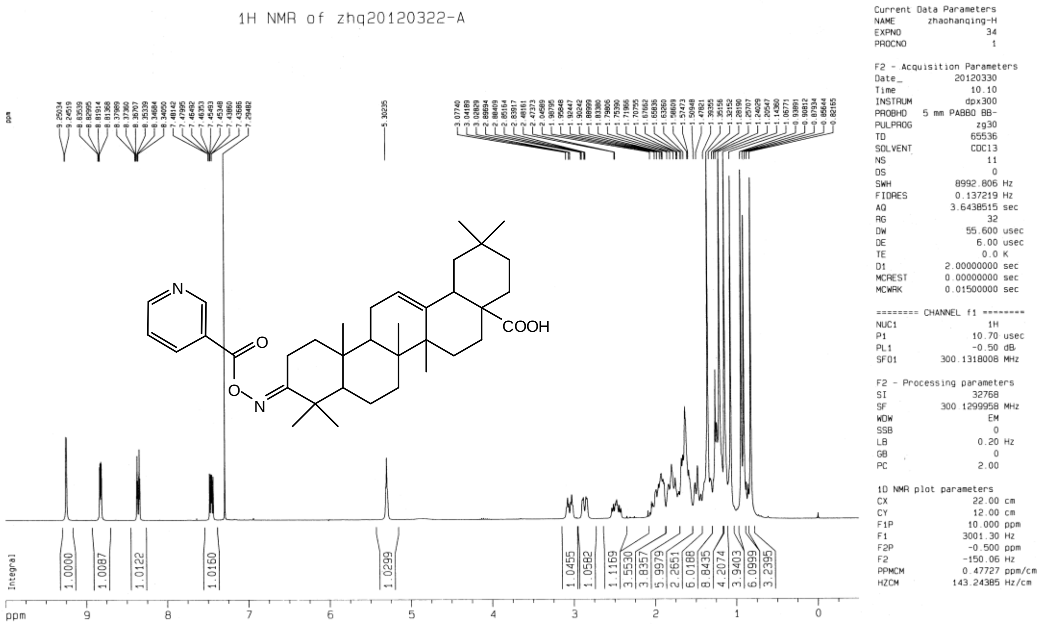

Figure S26. ^{13}C-NMR spectrum of compound A-09.

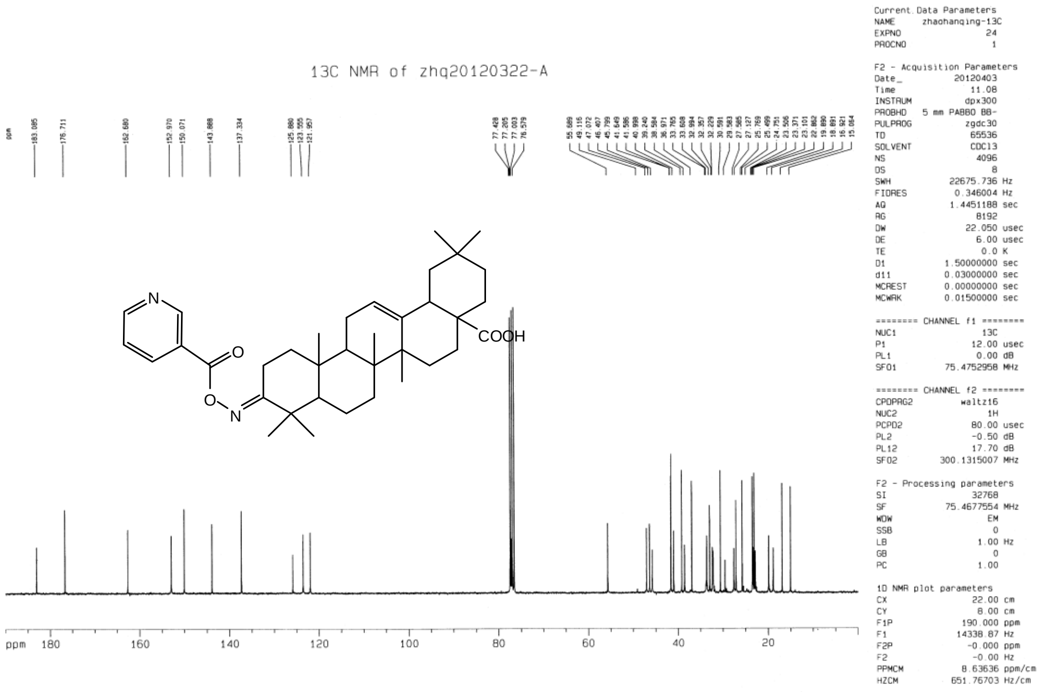

Figure S27. HRMS of compound A-09.

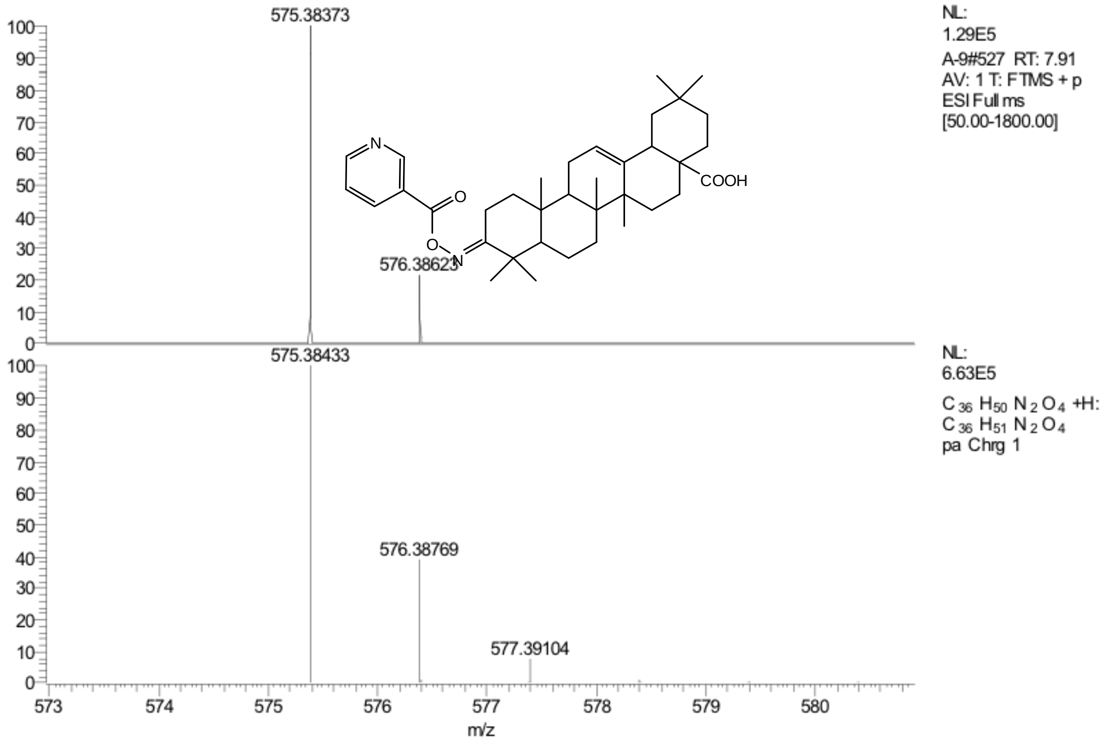

Figure S28. ^{1}H-NMR spectrum of compound A-10.

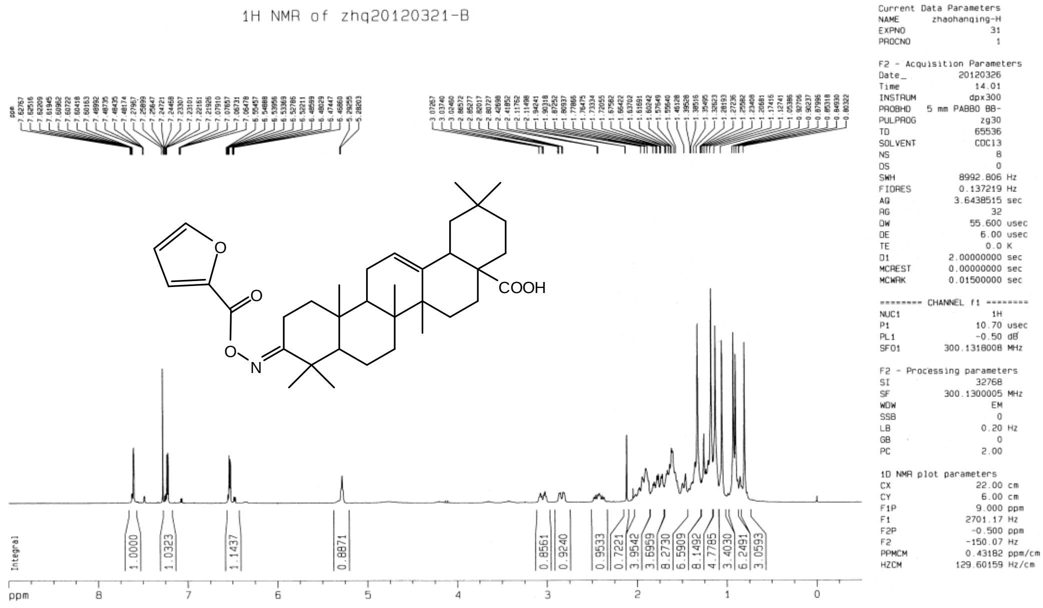

Figure S29. ^{13}C-NMR spectrum of compound A-10.

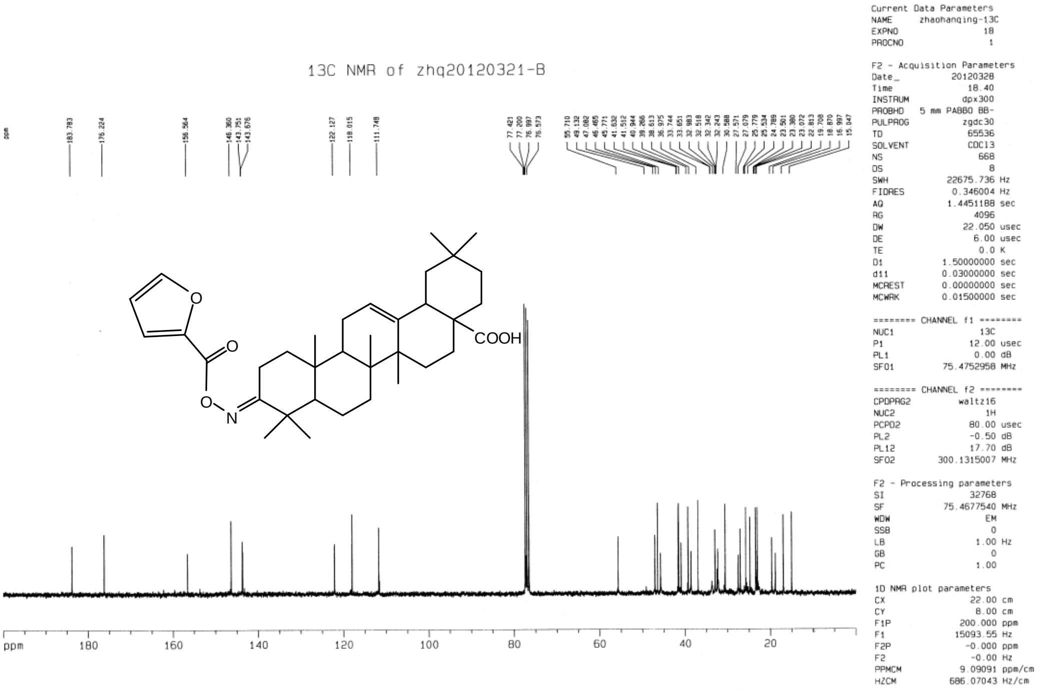

Figure S30. HRMS of compound A-10.

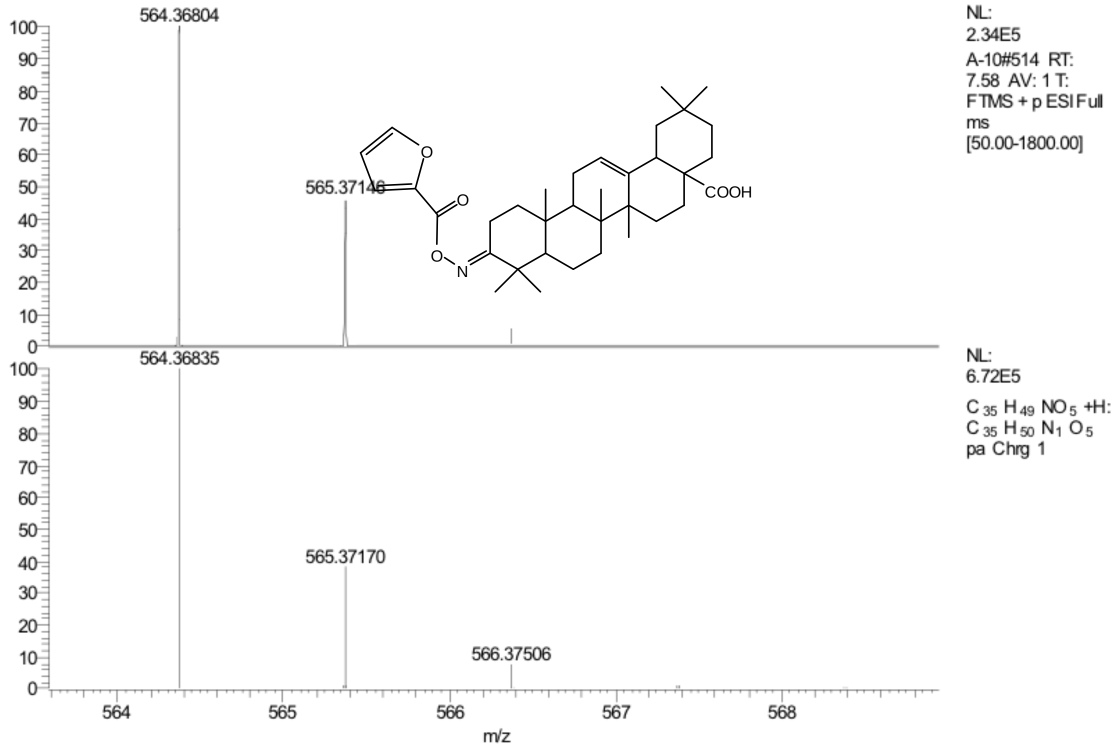

Figure S31. ^{1}H-NMR spectrum of compound B-01.

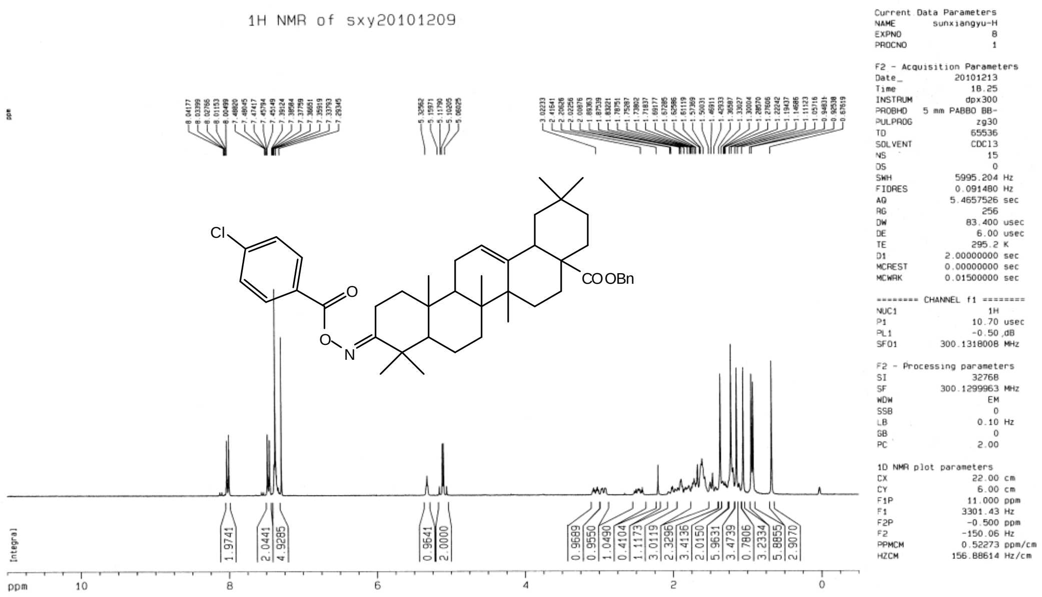

Figure S32. ^{13}C-NMR spectrum of compound B-01.

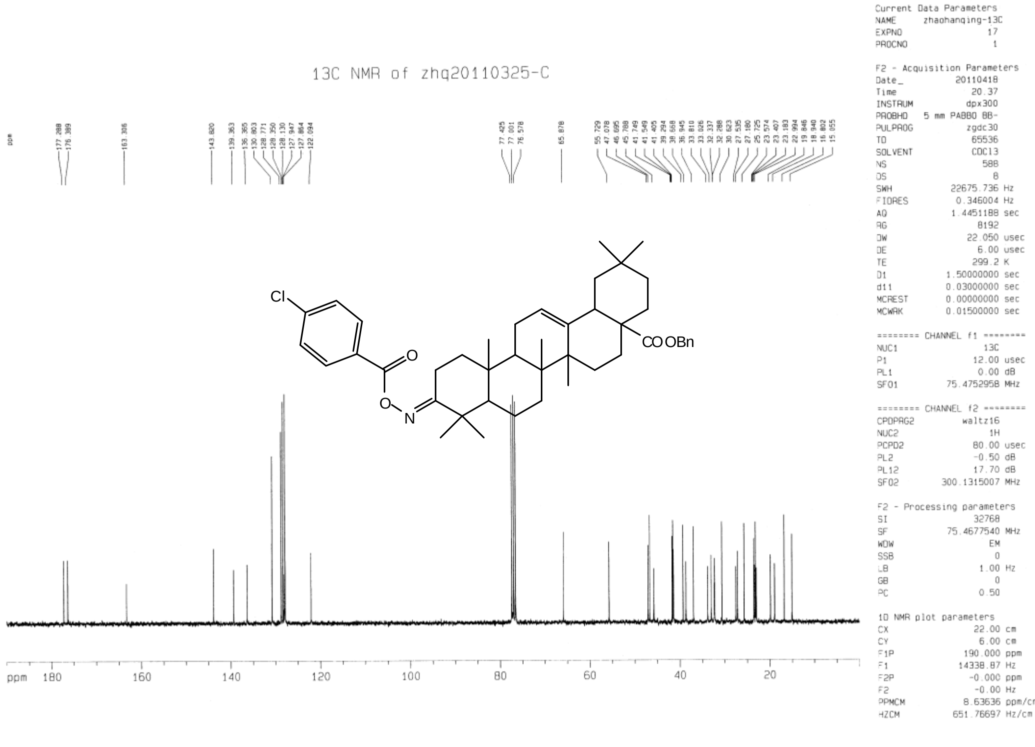

Figure S33. HRMS of compound B-01.

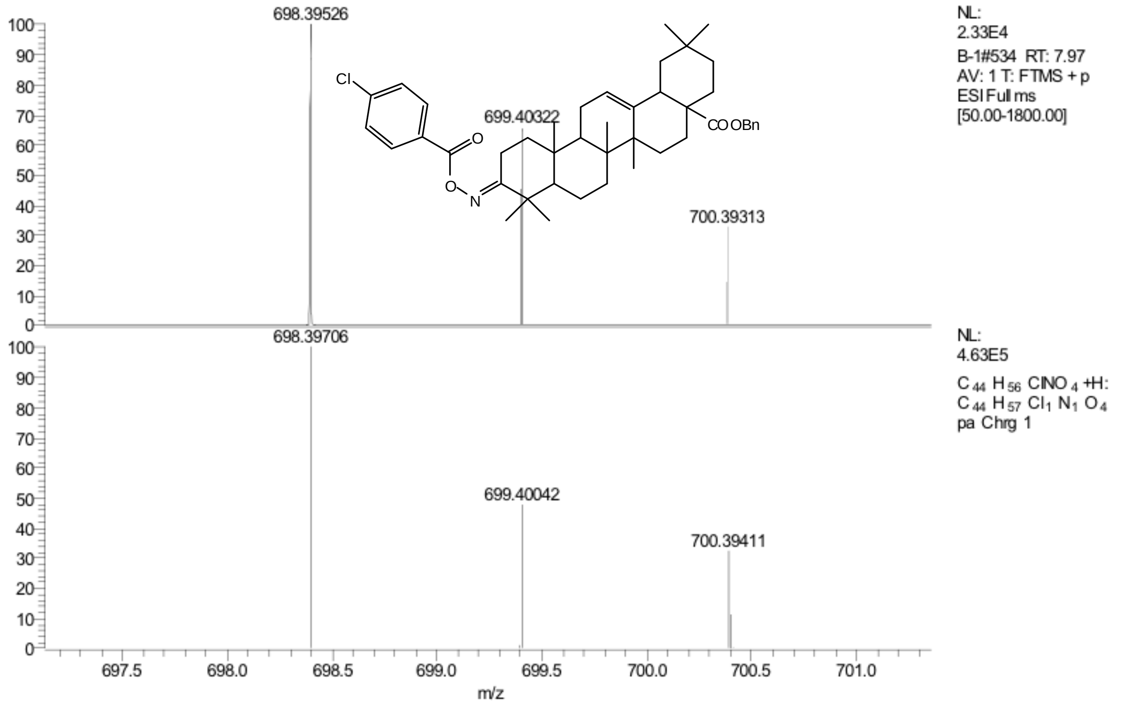

Figure S34. ^{1}H-NMR spectrum of compound B-02.

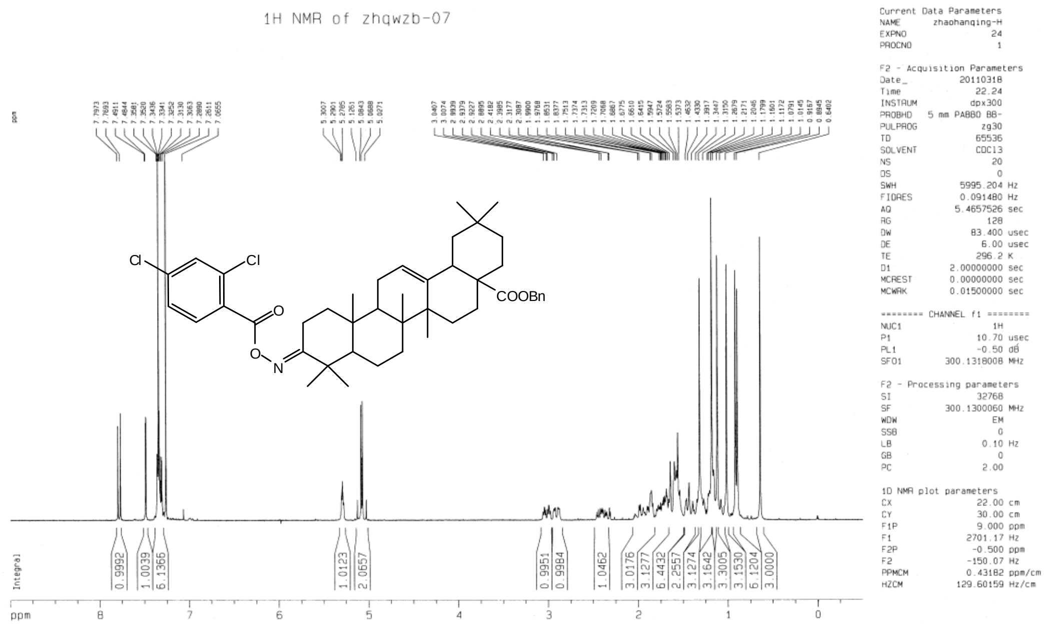

Figure S35. ^{13}C-NMR spectrum of compound B-02.

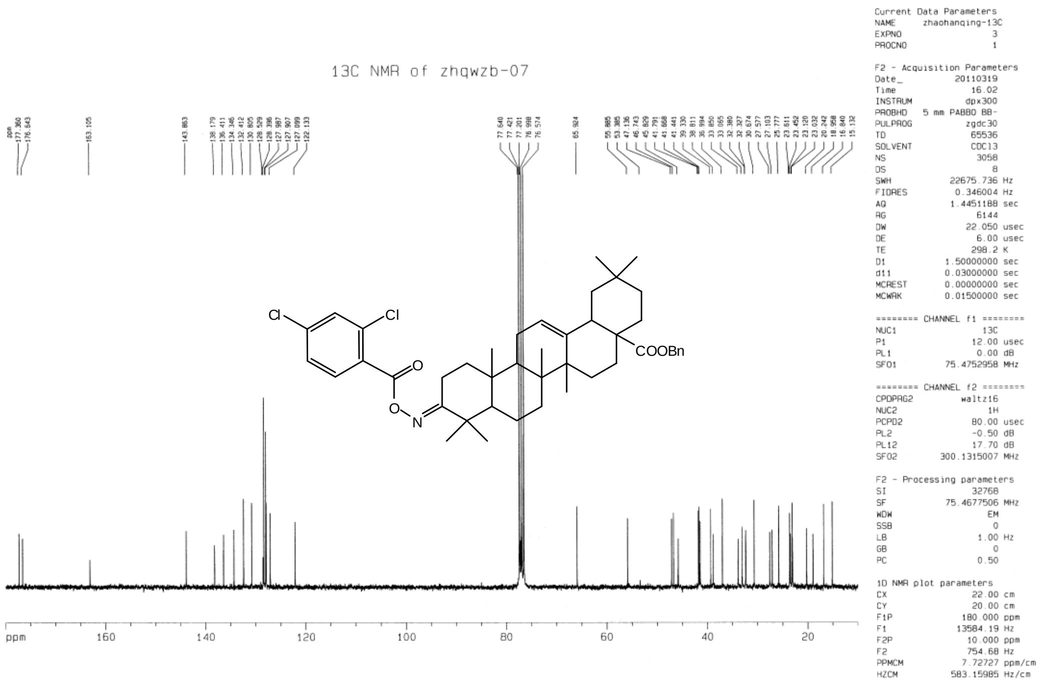

Figure S36. HRMS of compound B-02.

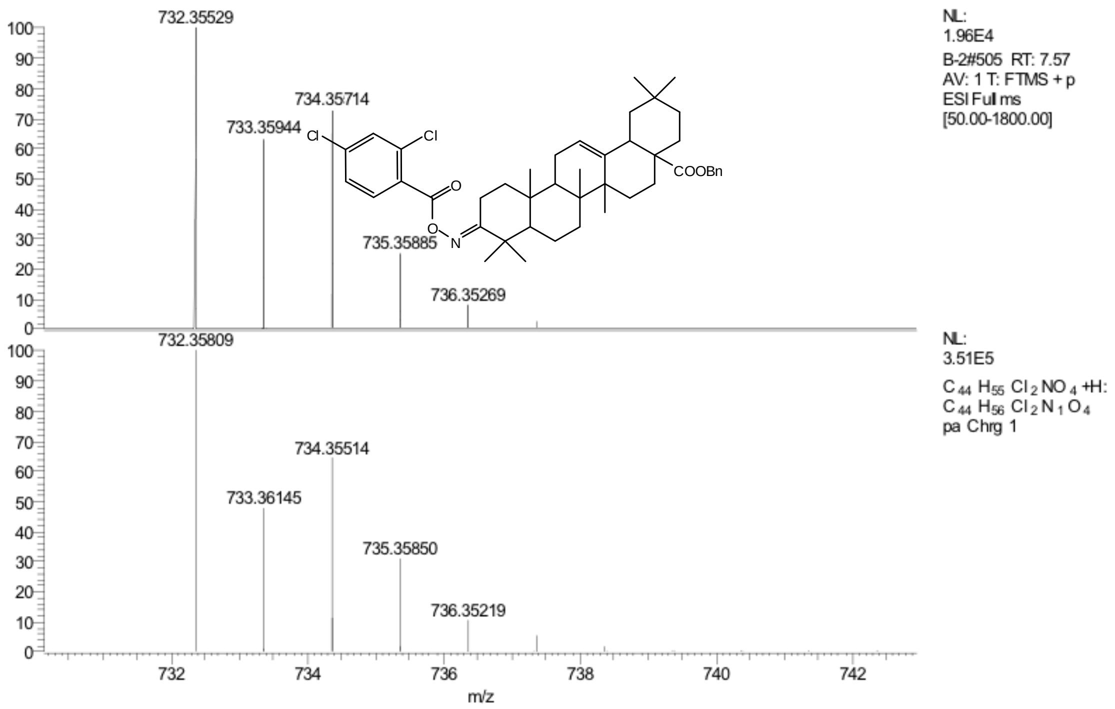

Figure S37. ^{1}H-NMR spectrum of compound B-03.

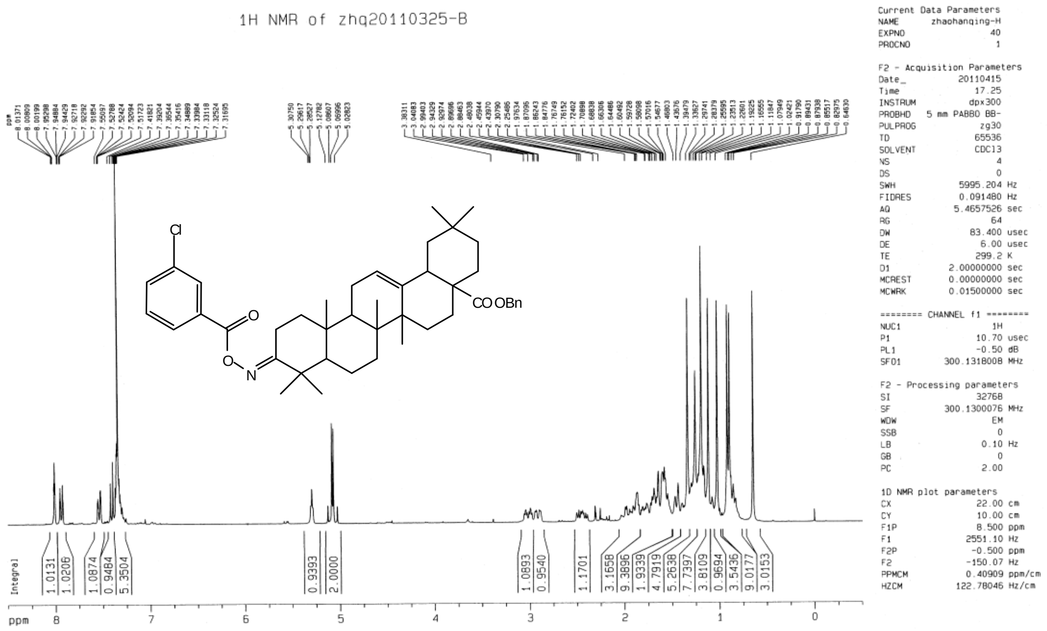

Figure S38. ^{13}C-NMR spectrum of compound B-03.

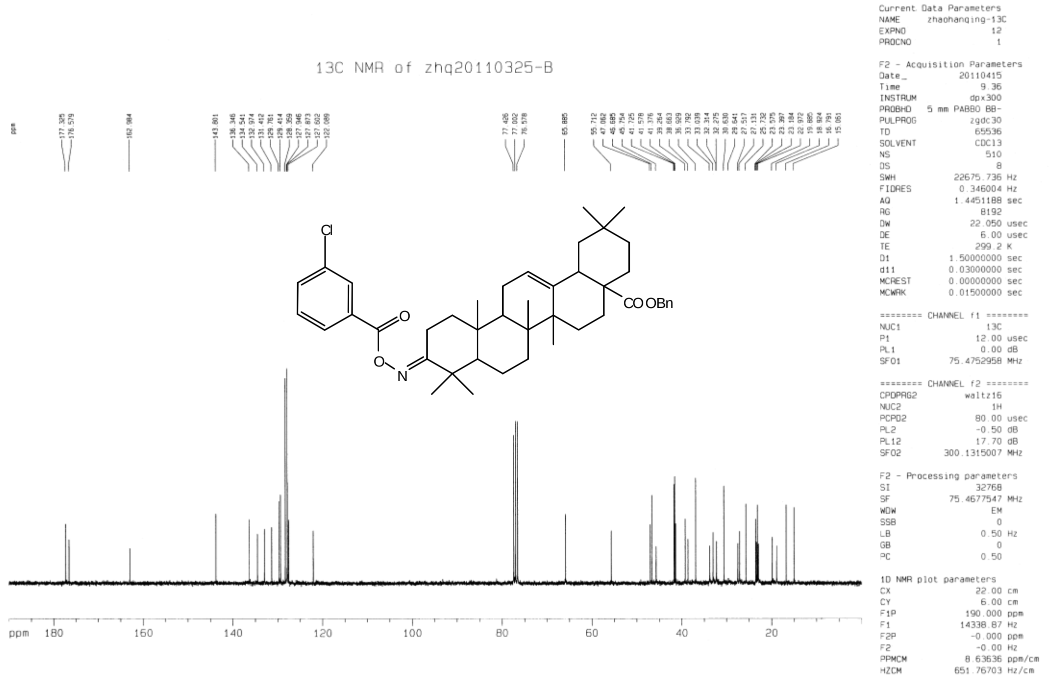

Figure S39. HRMS of compound B-03.

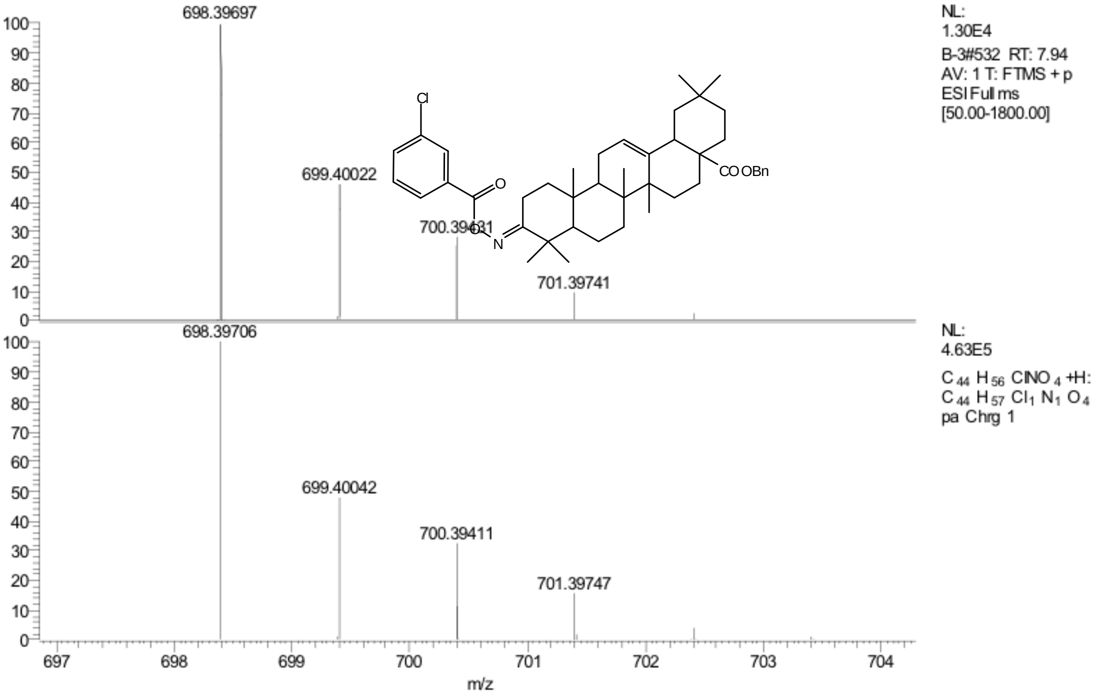

Figure S40. ^{1}H-NMR spectrum of compound B-04.

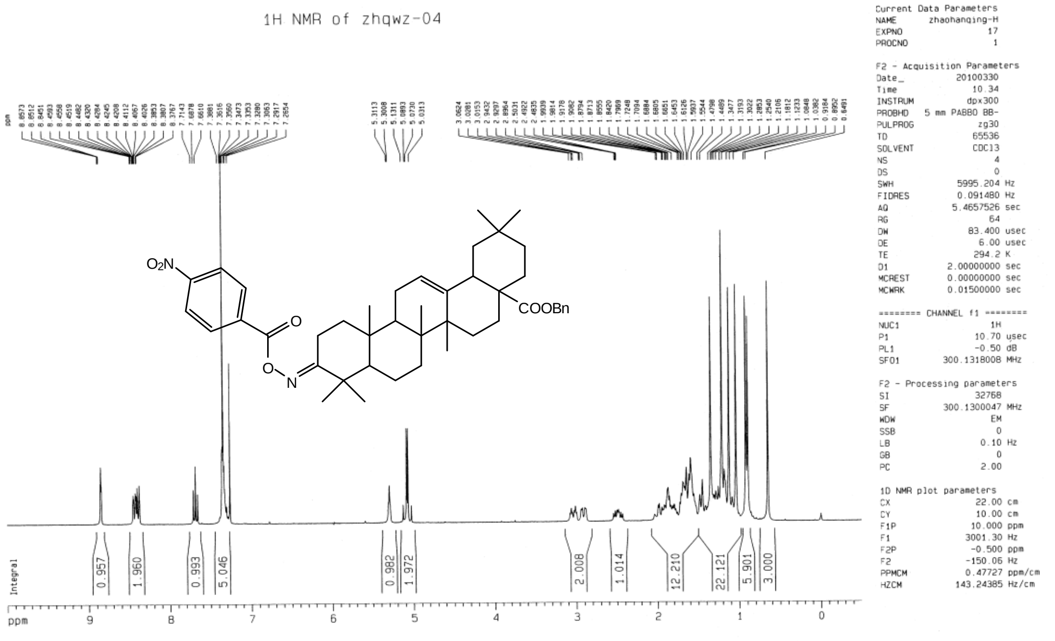

Figure S41. ^{13}C-NMR spectrum of compound B-04.

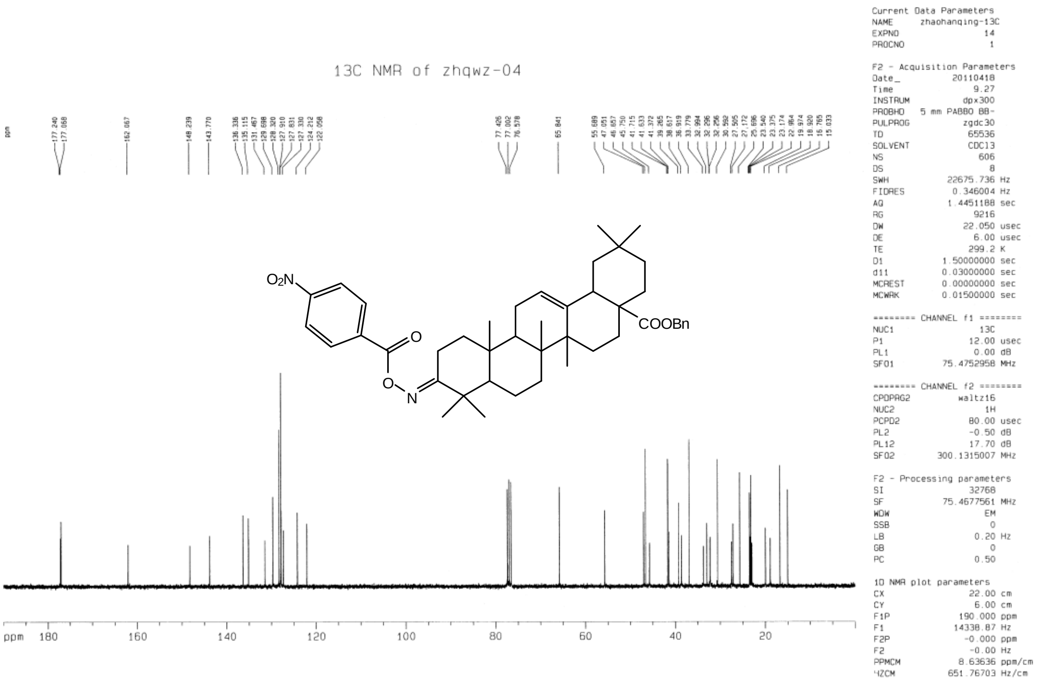

Figure S42. HRMS of compound B-04.

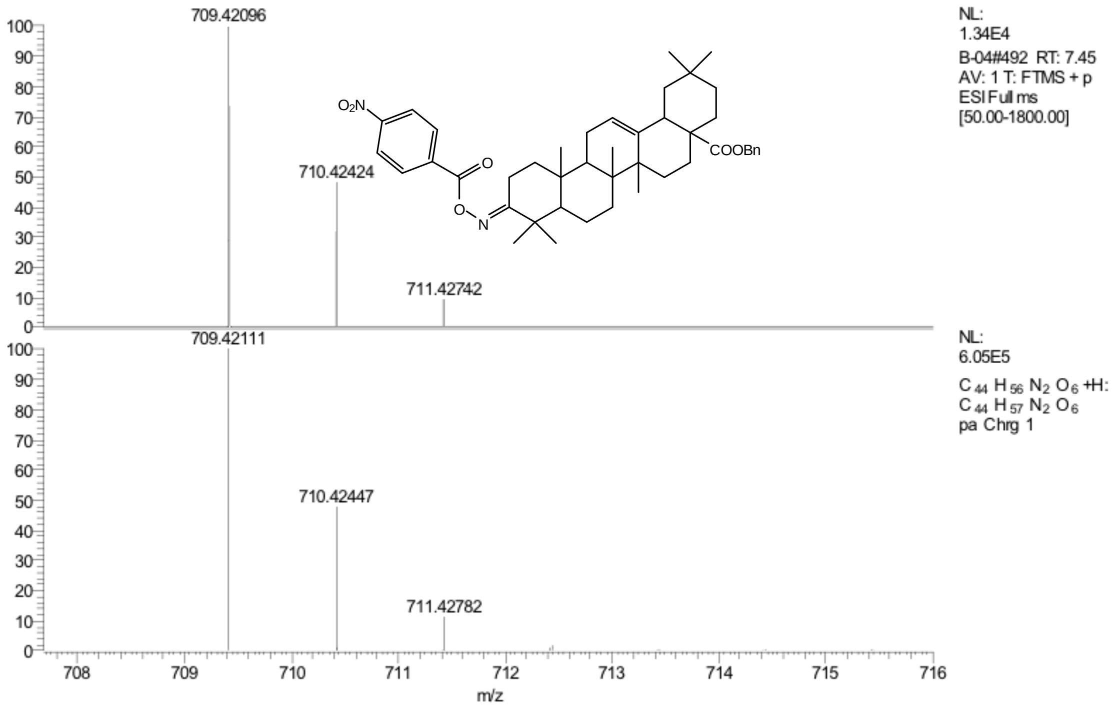

Figure S43. ^{1}H-NMR spectrum of compound B-05.

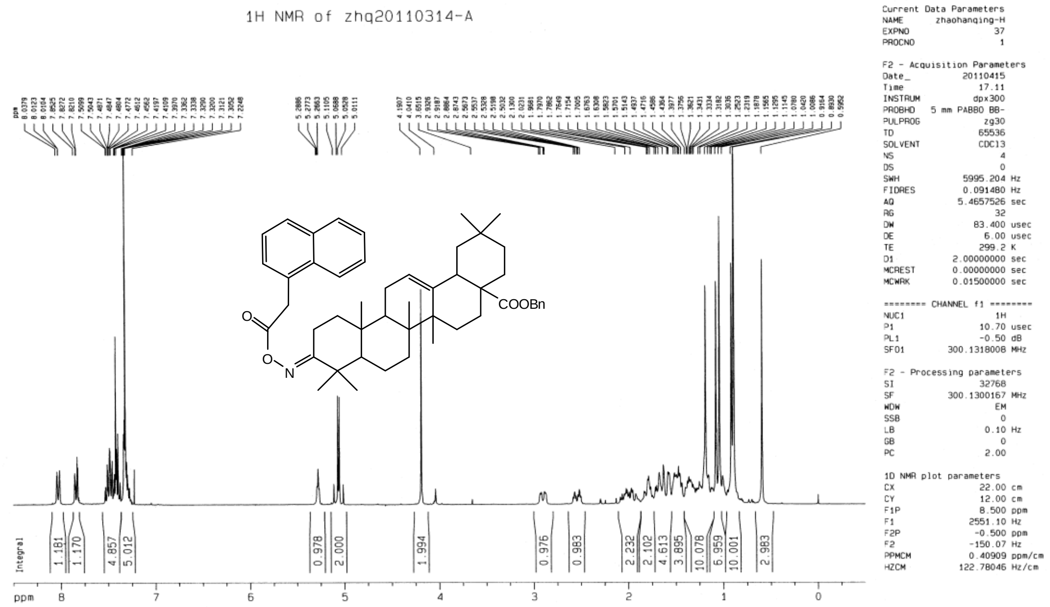

Figure S44. ^{13}C-NMR spectrum of compound B-05.

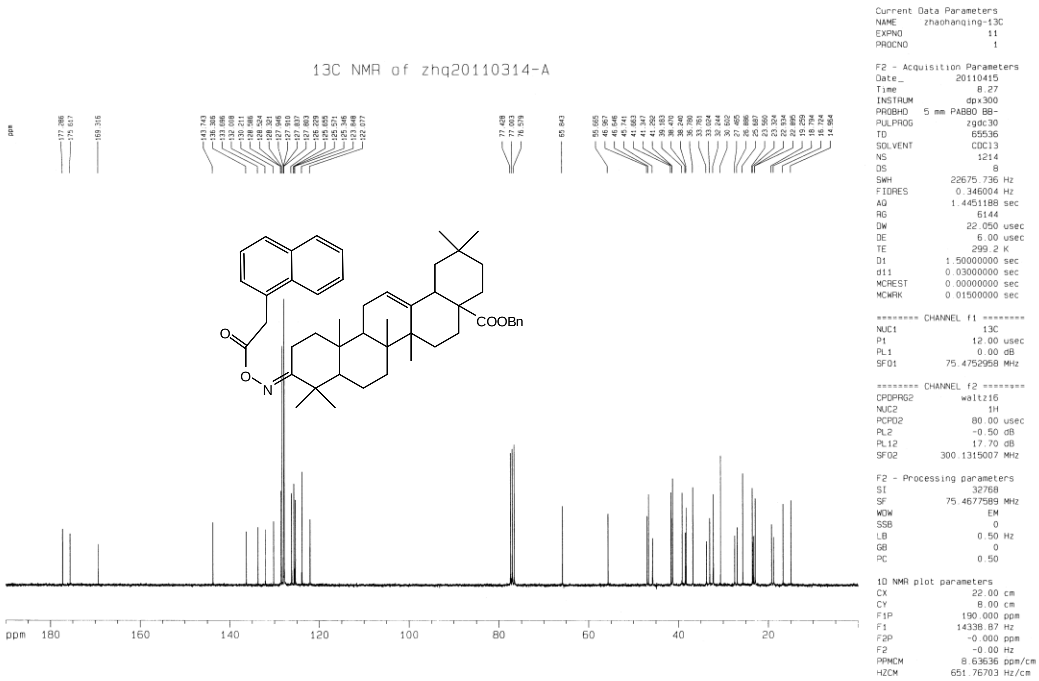

Figure S45. HRMS of compound B-05.

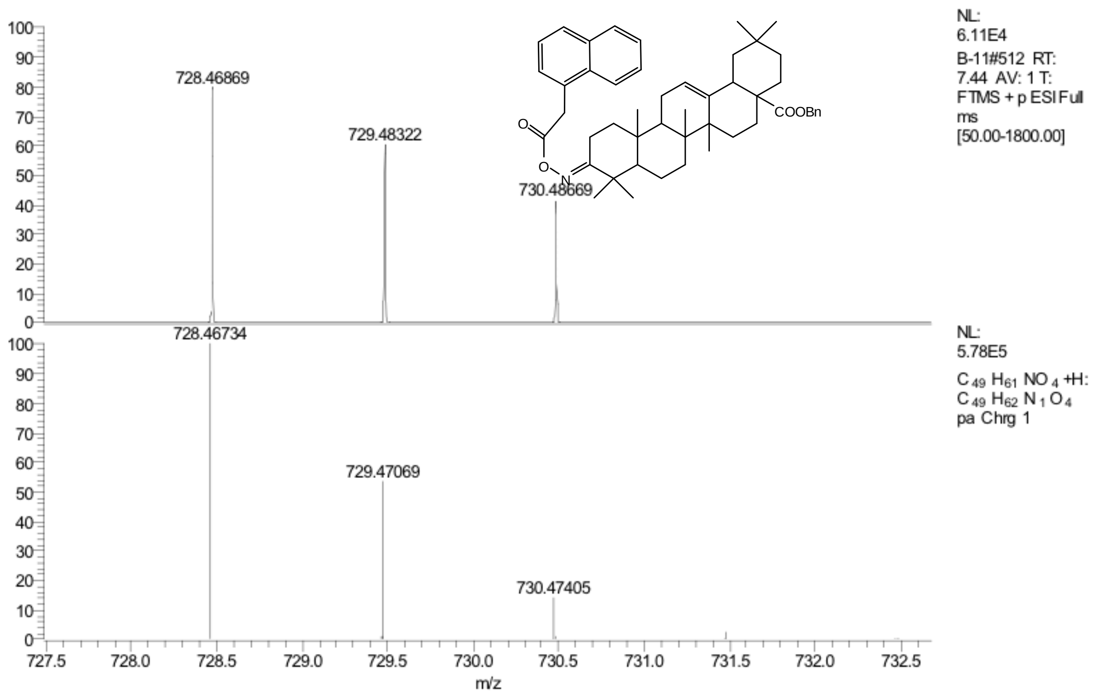

Figure S46. ^{1}H-NMR spectrum of compound B-06.

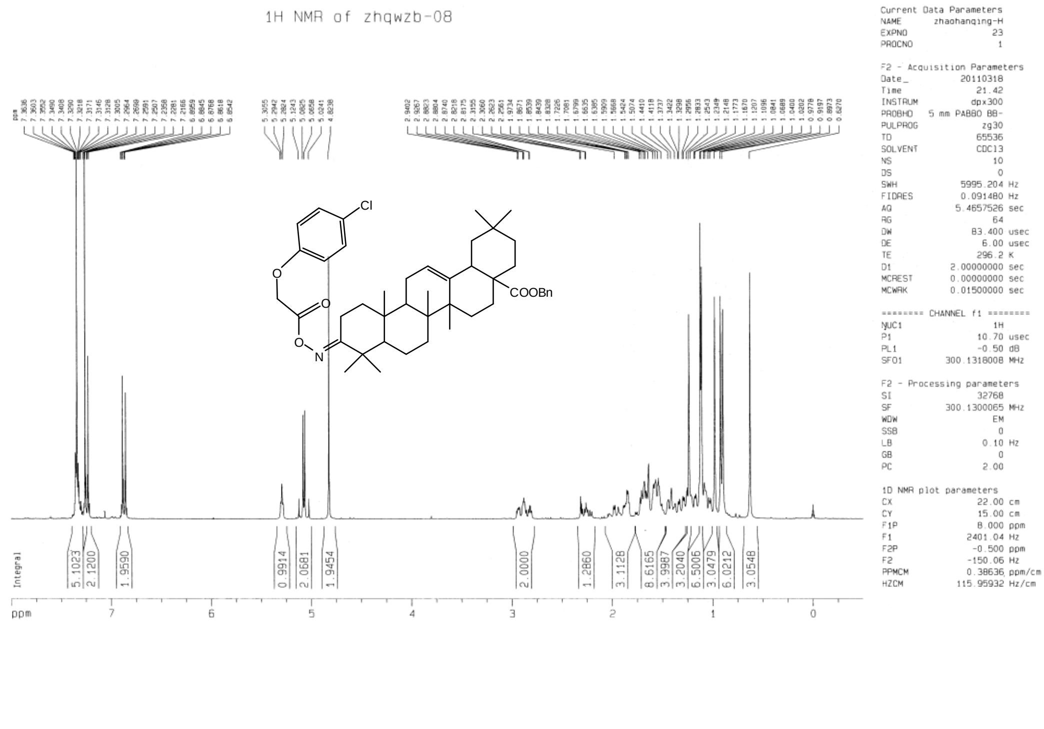

Figure S47. ^{13}C-NMR spectrum of compound B-06.

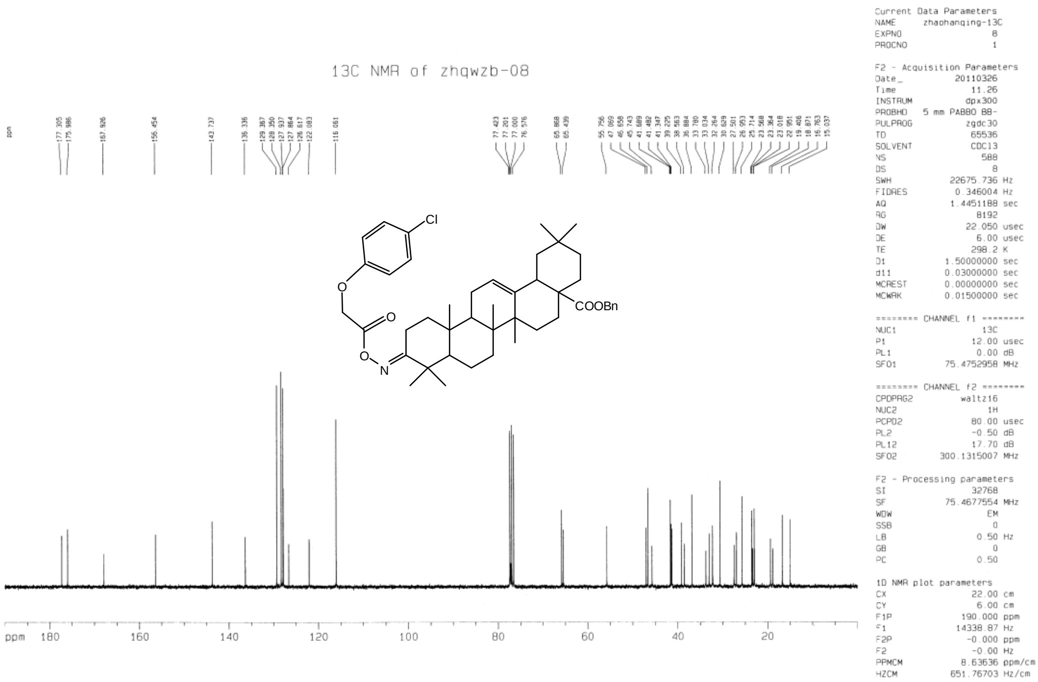

Figure S48. HRMS of compound B-06.

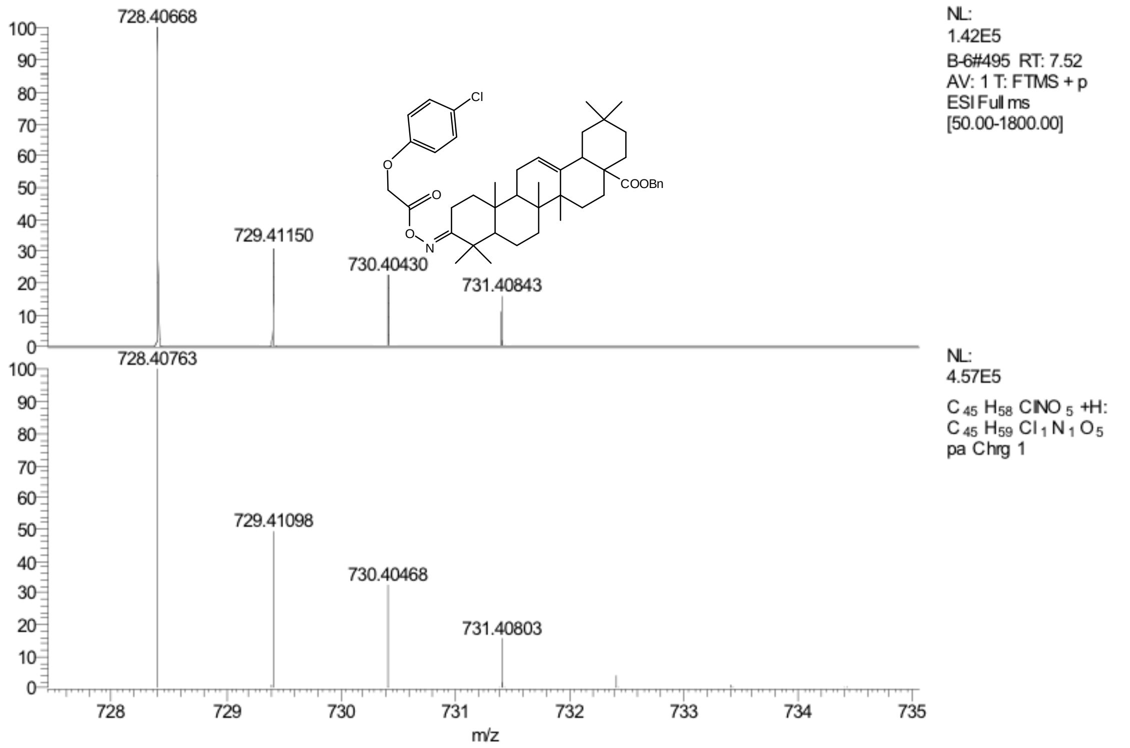

Figure S49. ^{1}H-NMR spectrum of compound B-07.

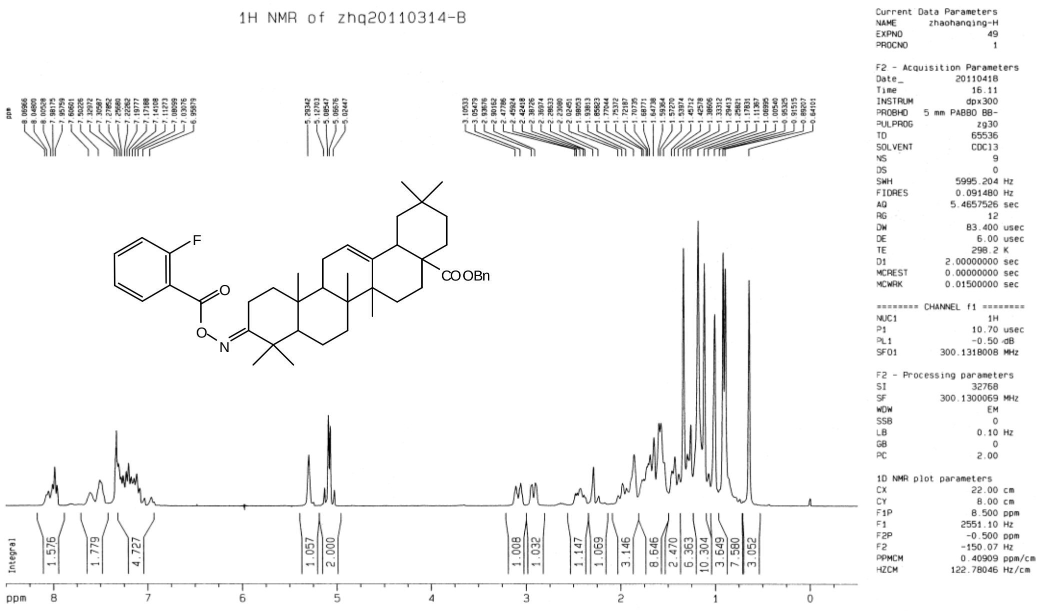

Figure S50. ^{13}C-NMR spectrum of compound B-07.

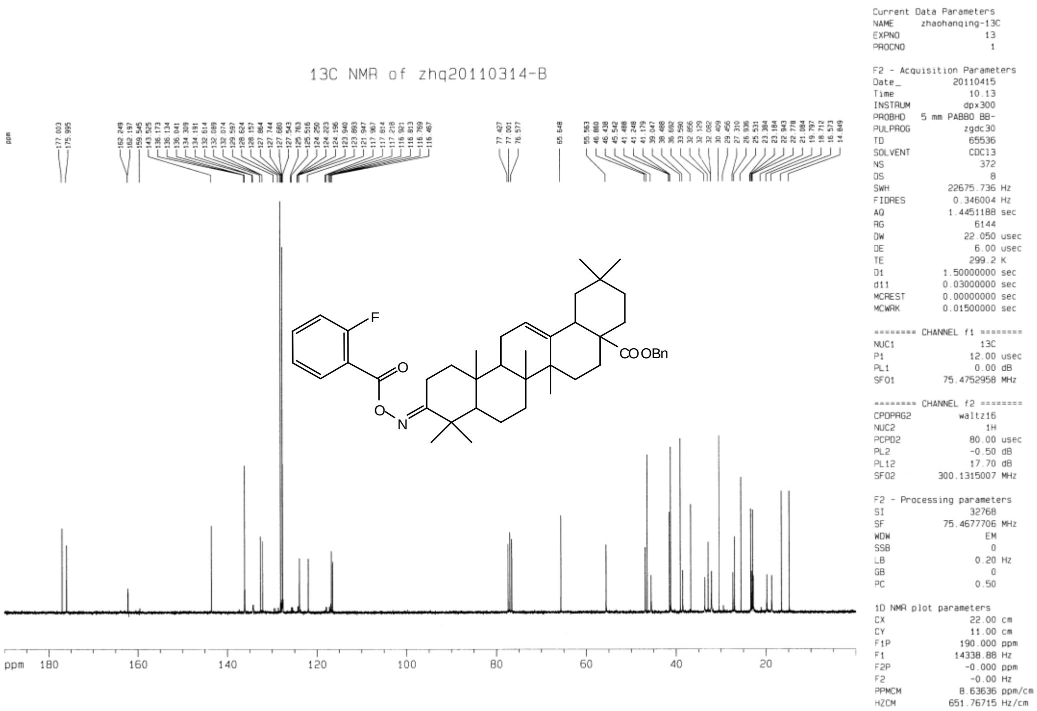

Figure S51. HRMS of compound B-07.

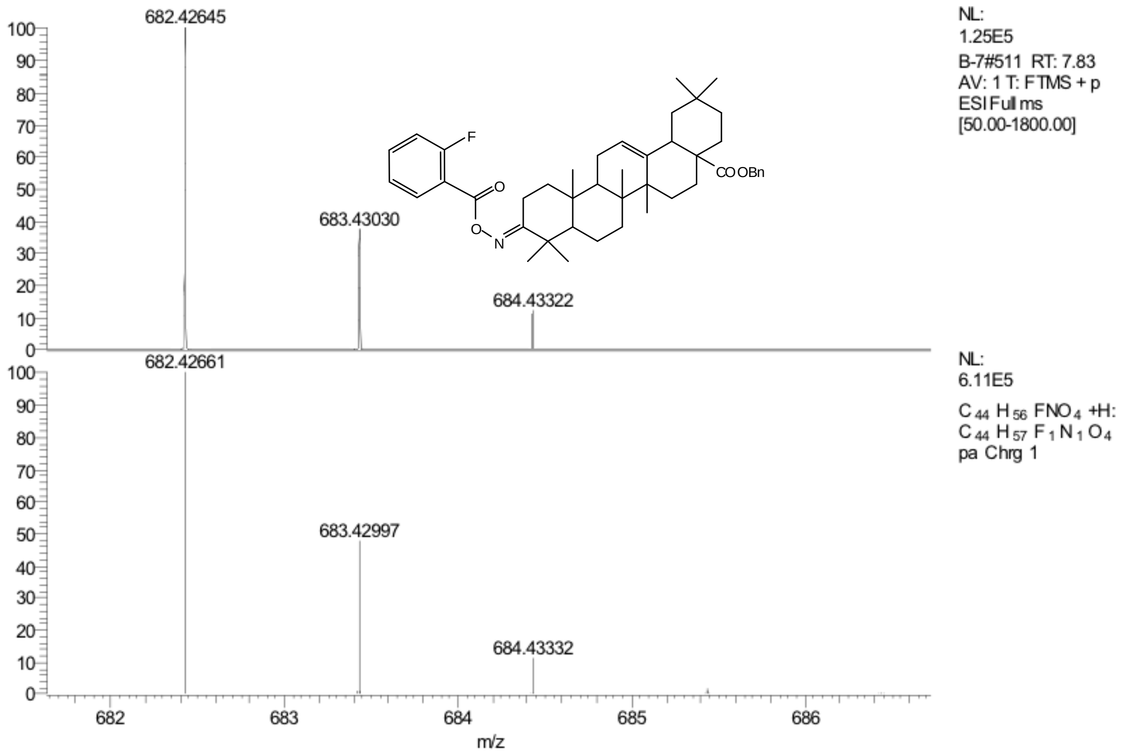

Figure S52. ^{1}H-NMR spectrum of compound B-08.

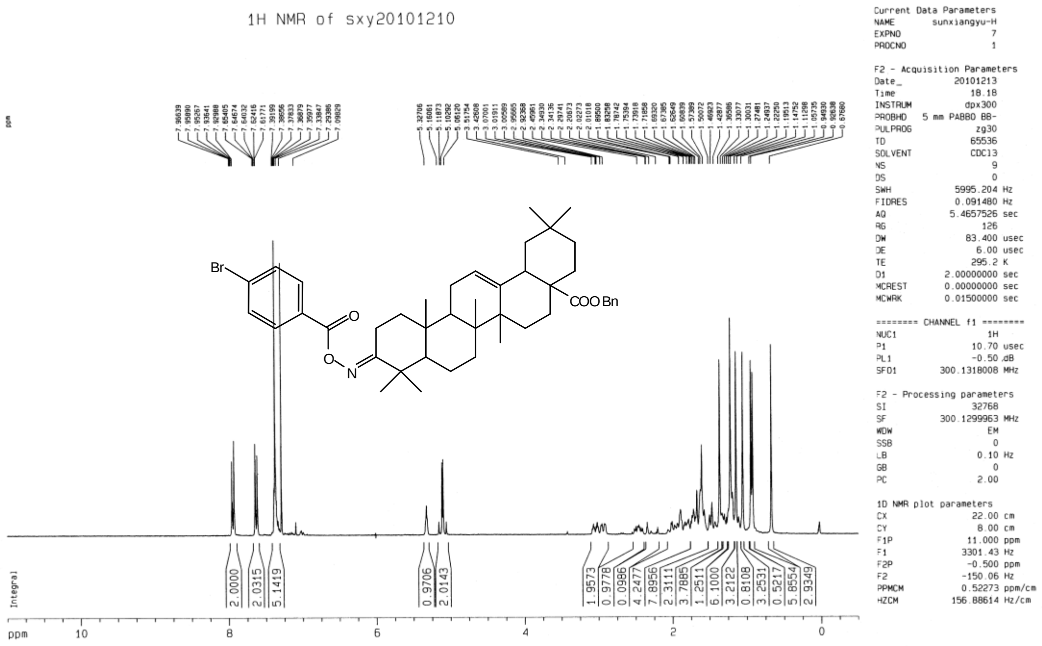

Figure S53. ^{13}C-NMR spectrum of compound B-08.

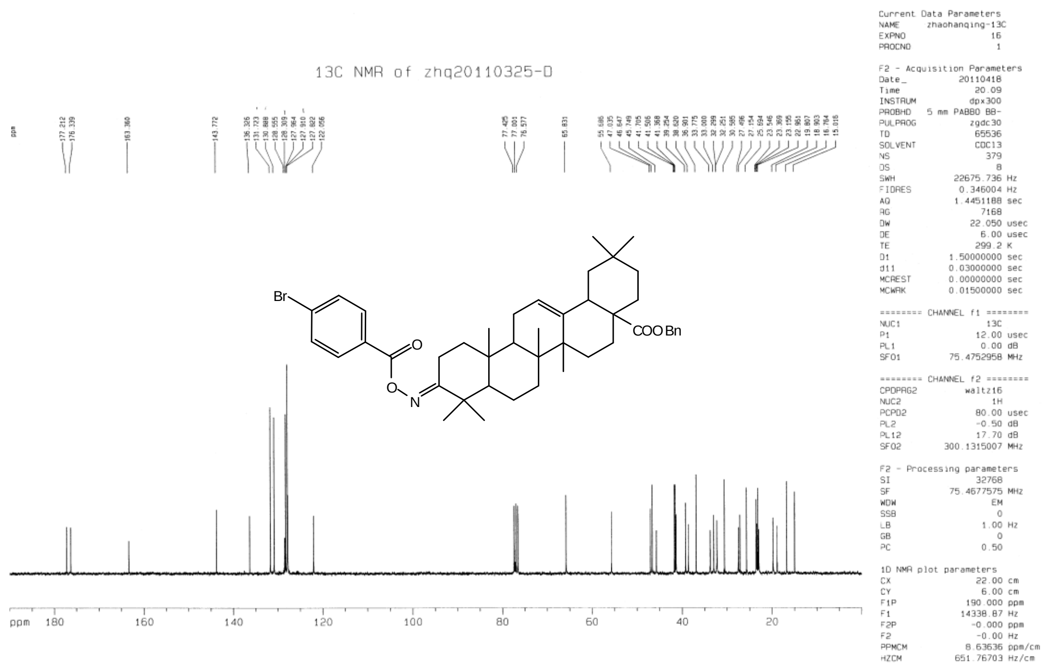

Figure S54. HRMS of compound B-08.

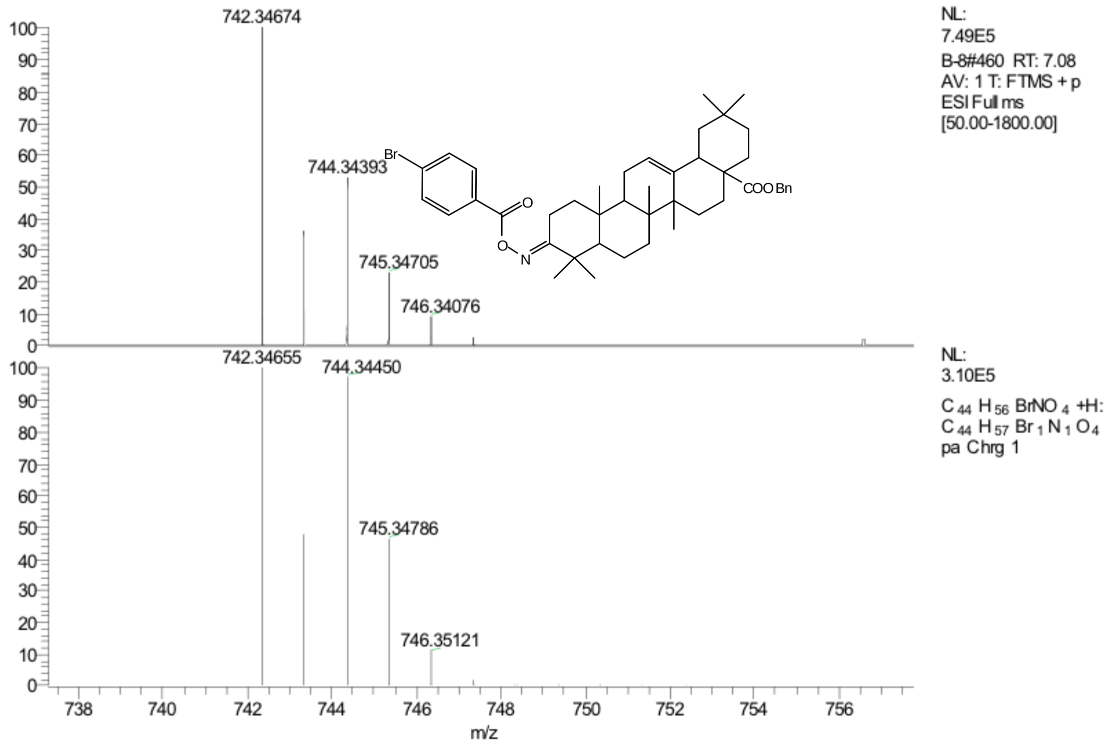

Figure S55. ^{1}H-NMR spectrum of compound B-09.

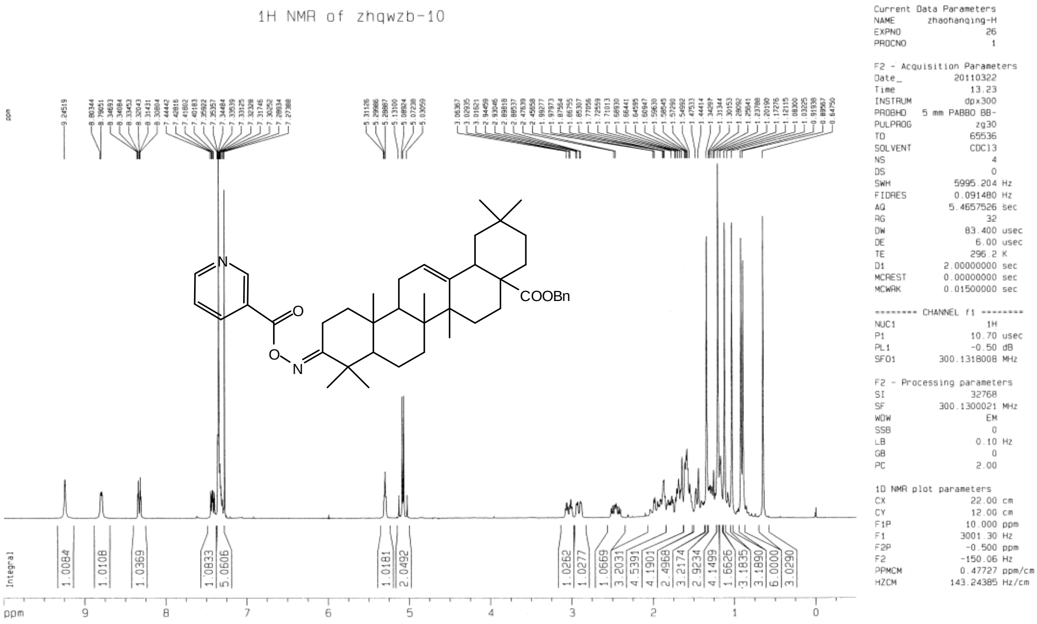

Figure S56. ^{13}C-NMR spectrum of compound B-09.

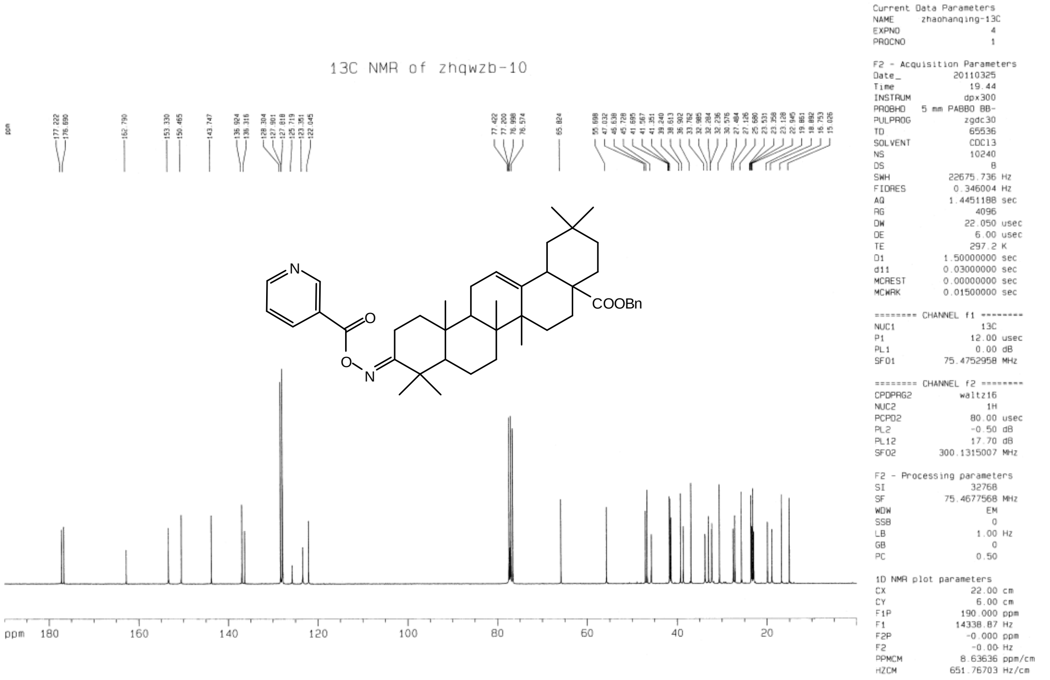

Figure S57. HRMS of compound B-09.

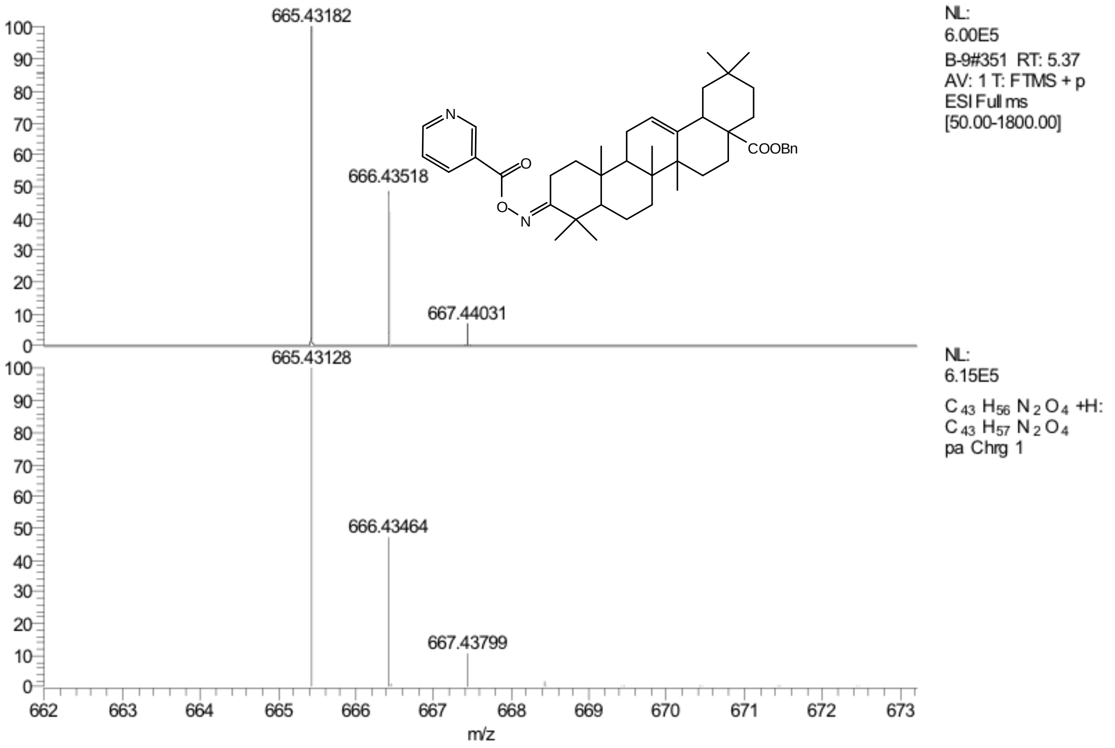

Figure S58. ^{1}H-NMR spectrum of compound B-10.

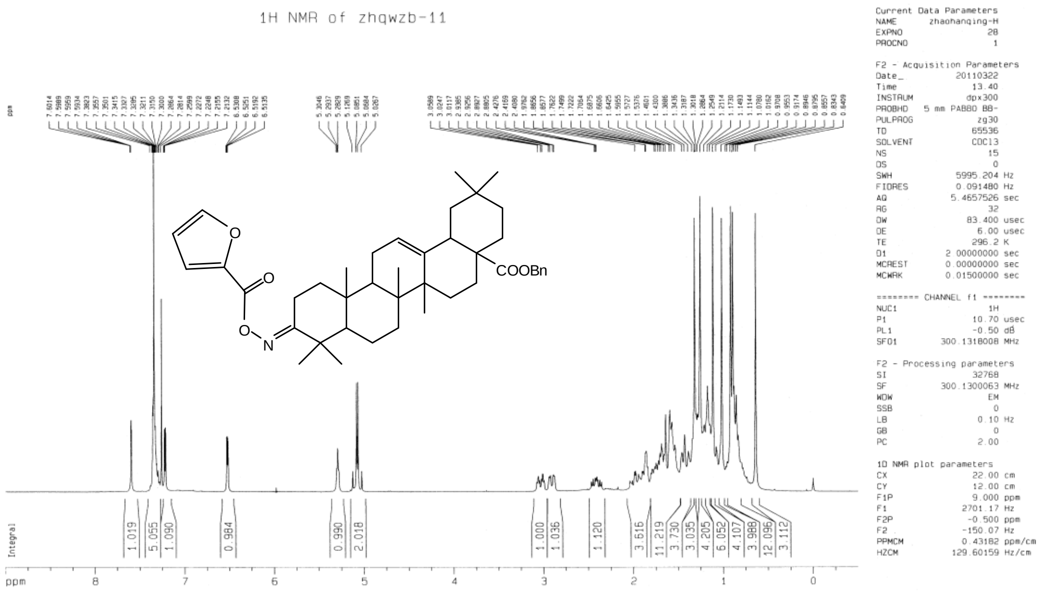

Figure S58. ^{13}C-NMR spectrum of compound B-10.

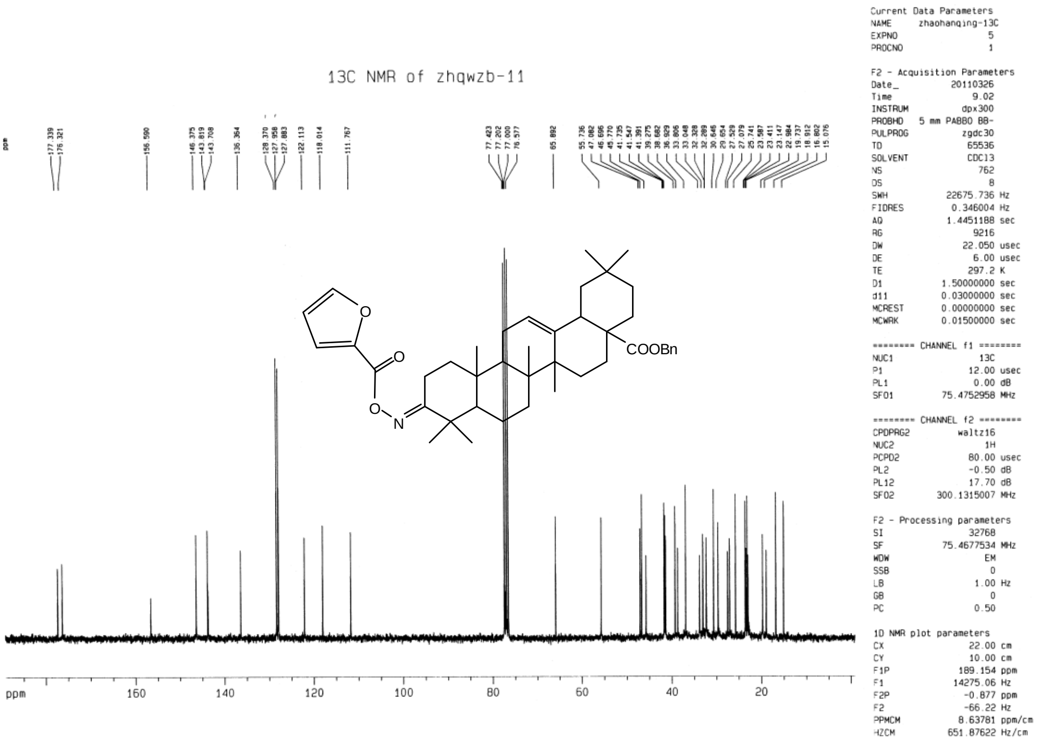

Figure S59. HRMS of compound B-10.

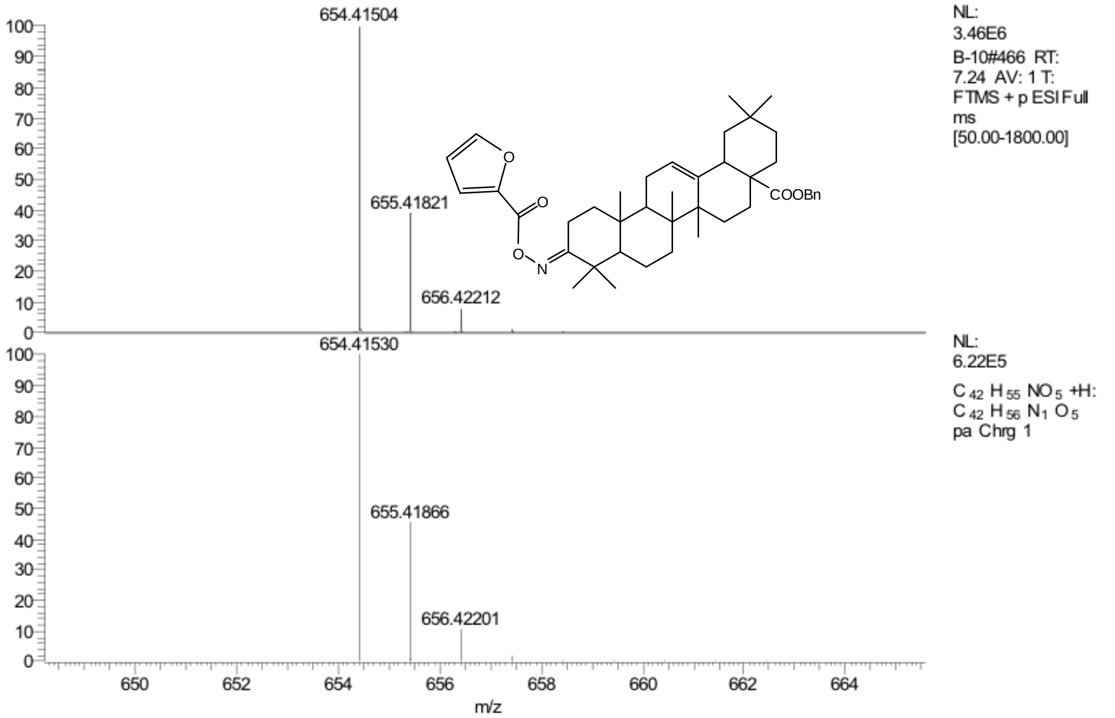

Figure S60. ^{1}H-NMR spectrum of compound B-11.

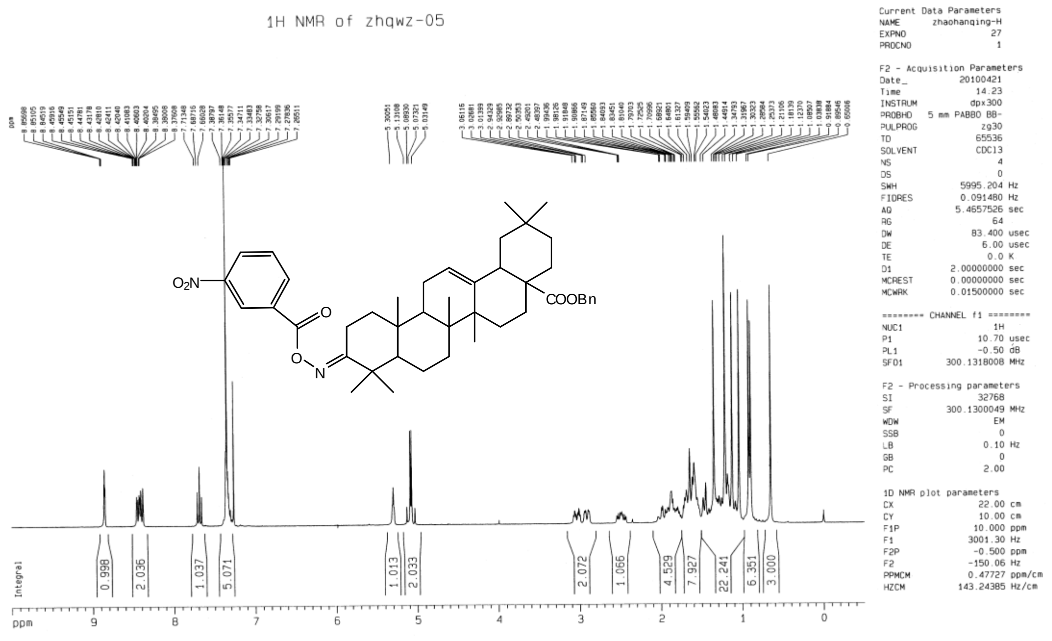

Figure S61. ^{13}C-NMR spectrum of compound B-11.

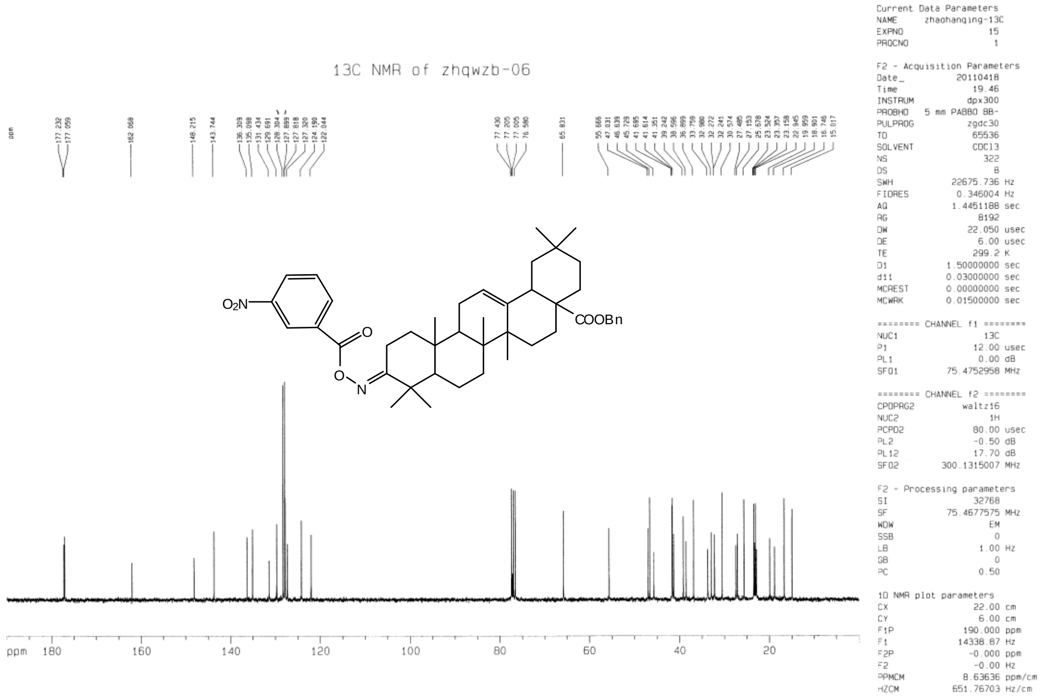

Figure S62. HRMS of compound B-11.

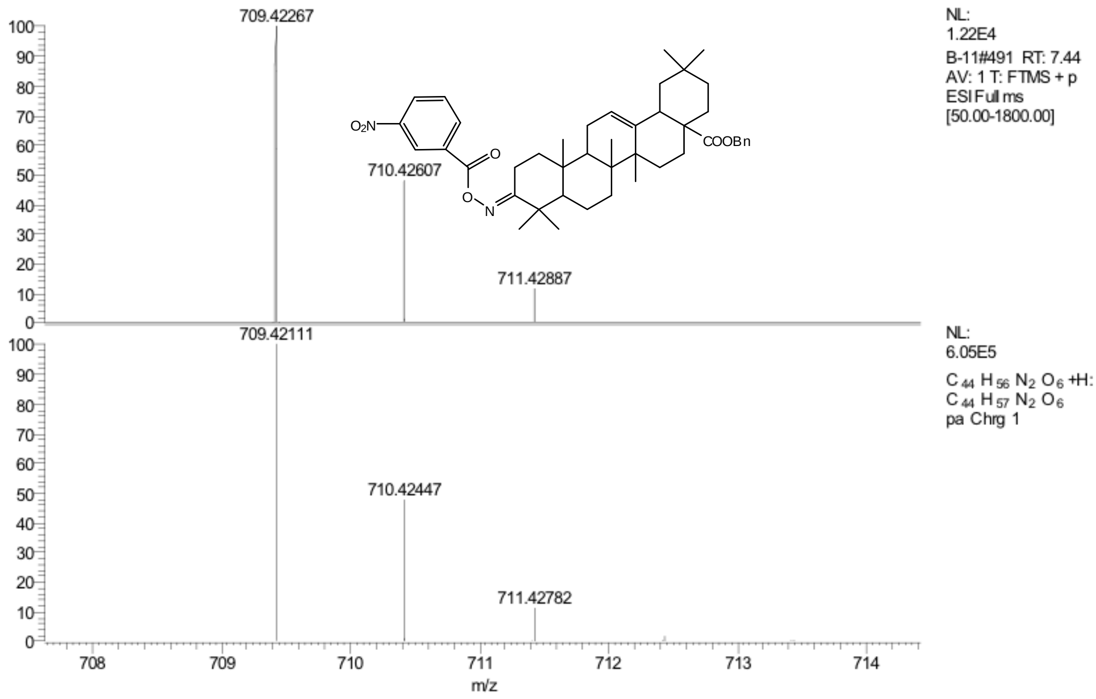

Figure S63. ^{1}H-NMR spectrum of compound B-12.

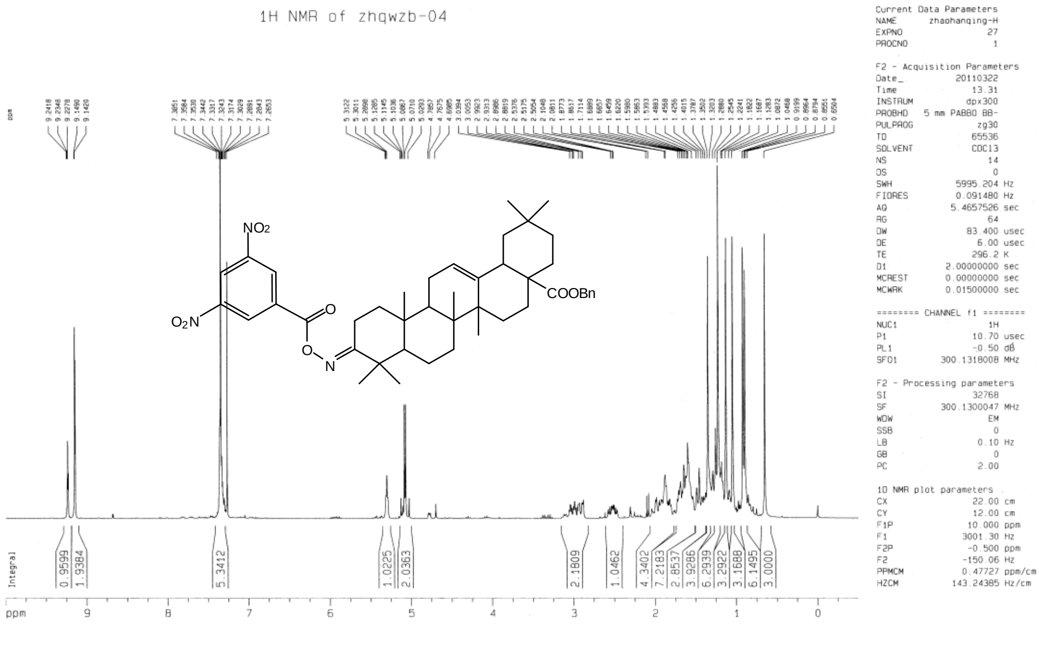

Figure S64. ^{13}C-NMR spectrum of compound B-12.

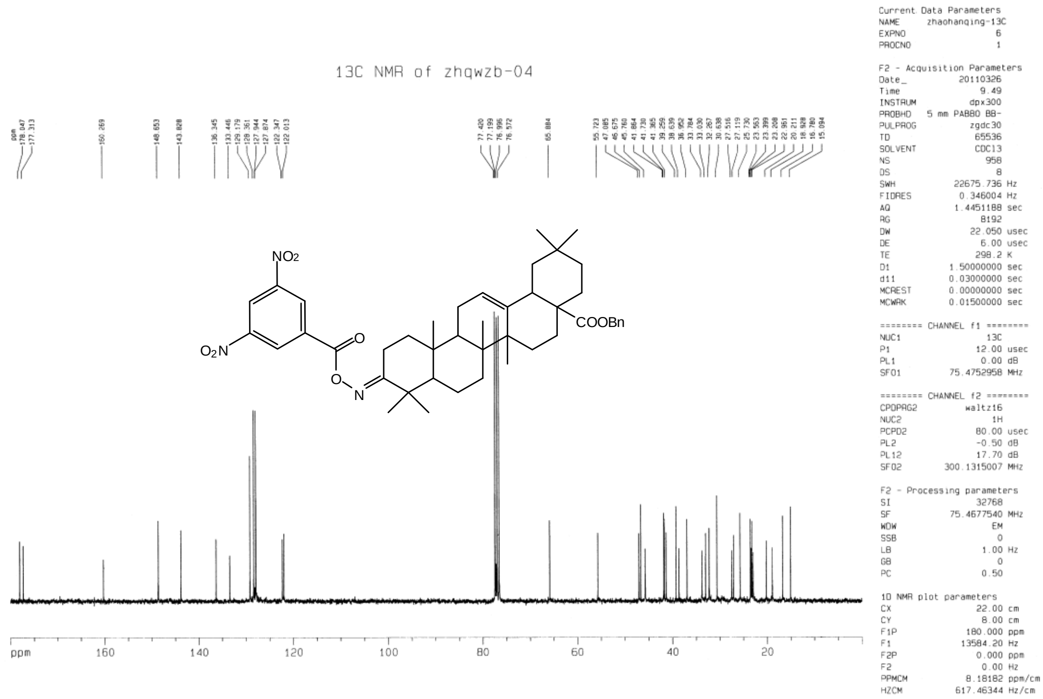

Figure S65. HRMS of compound B-12.

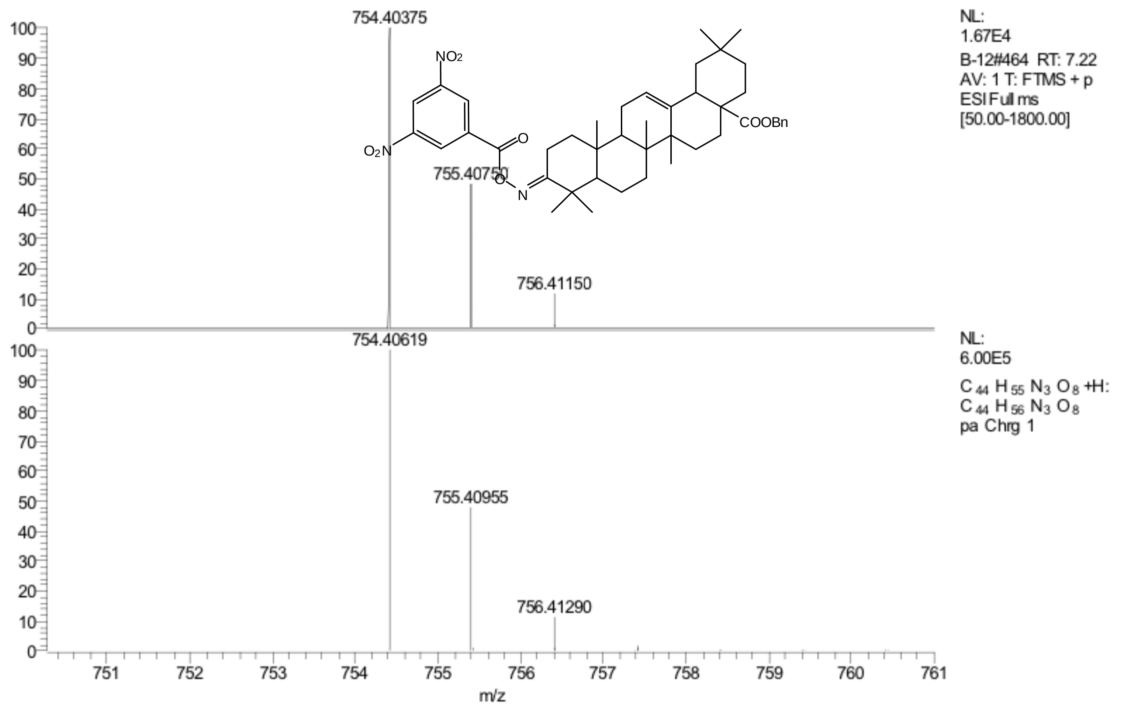

Figure S66. ^{1}H-NMR spectrum of compound B-13.

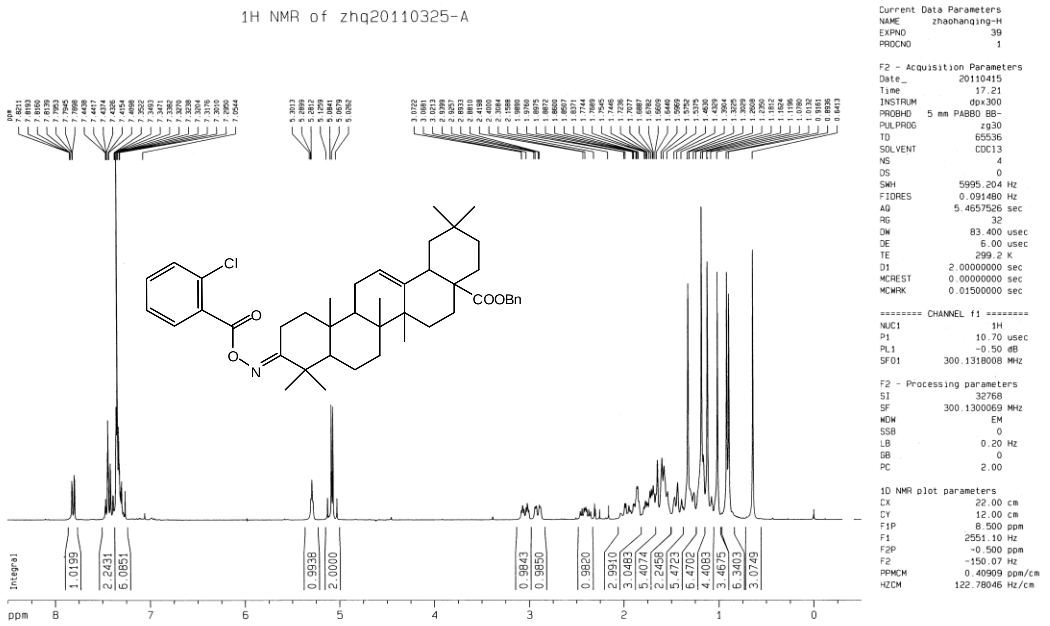

Figure S67. ^{13}C-NMR spectrum of compound B-13.

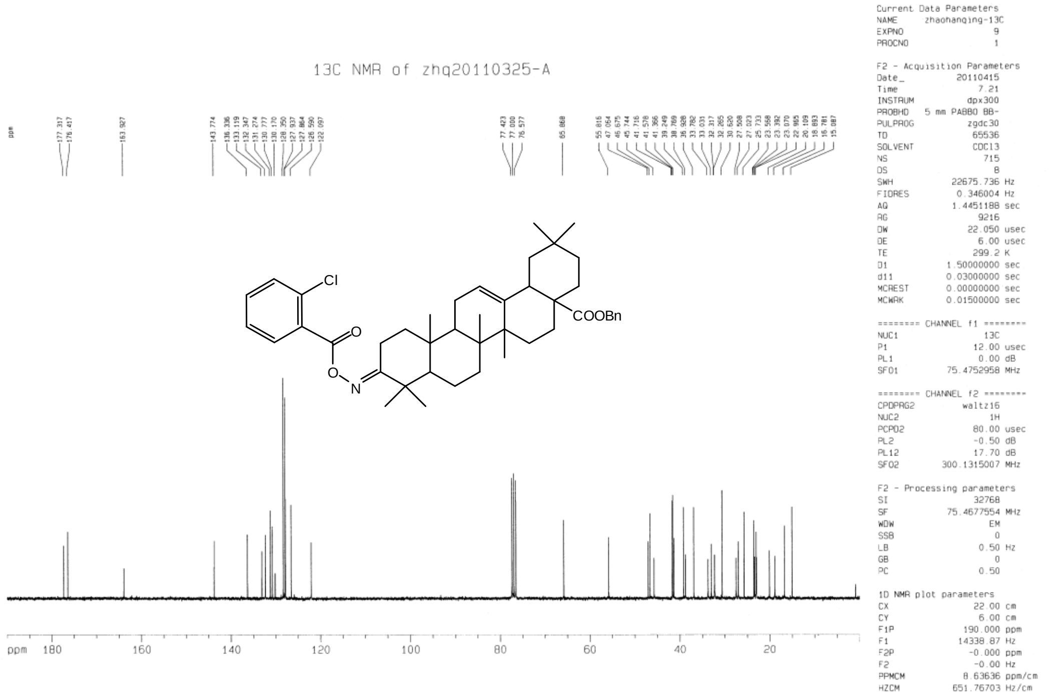

Figure S68. HRMS of compound B-13.

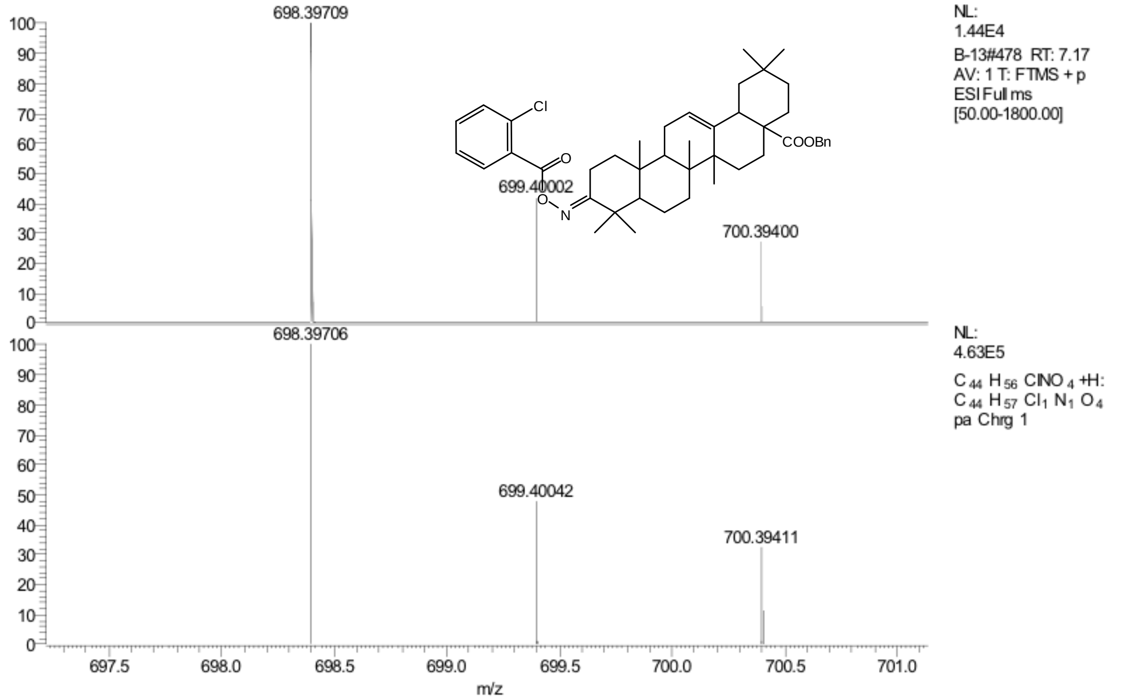

Figure S69. ^{1}H-NMR spectrum of compound B-14.

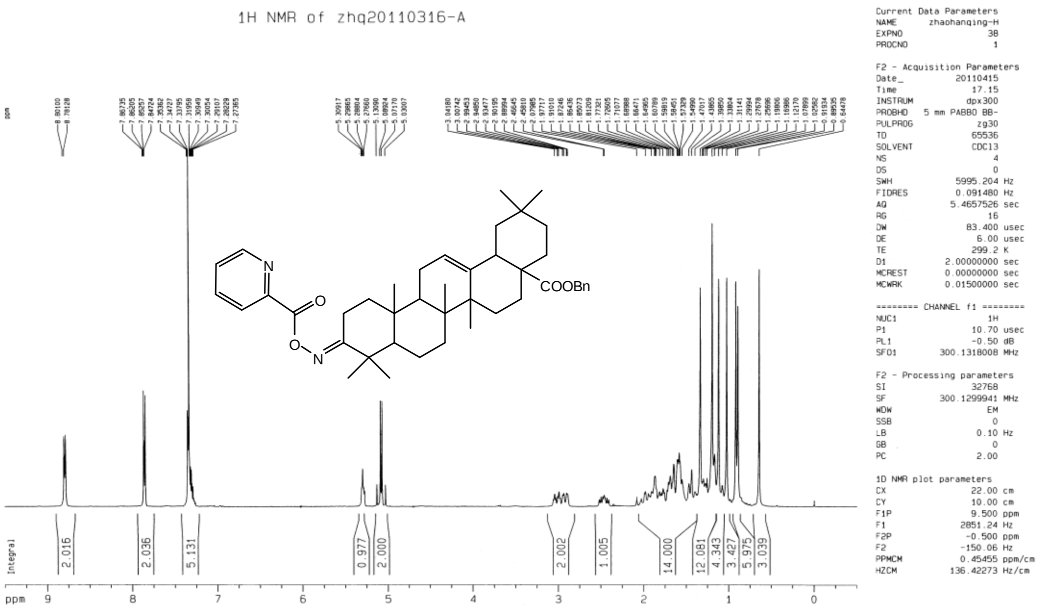

Figure S70. ^{13}C-NMR spectrum of compound B-14.

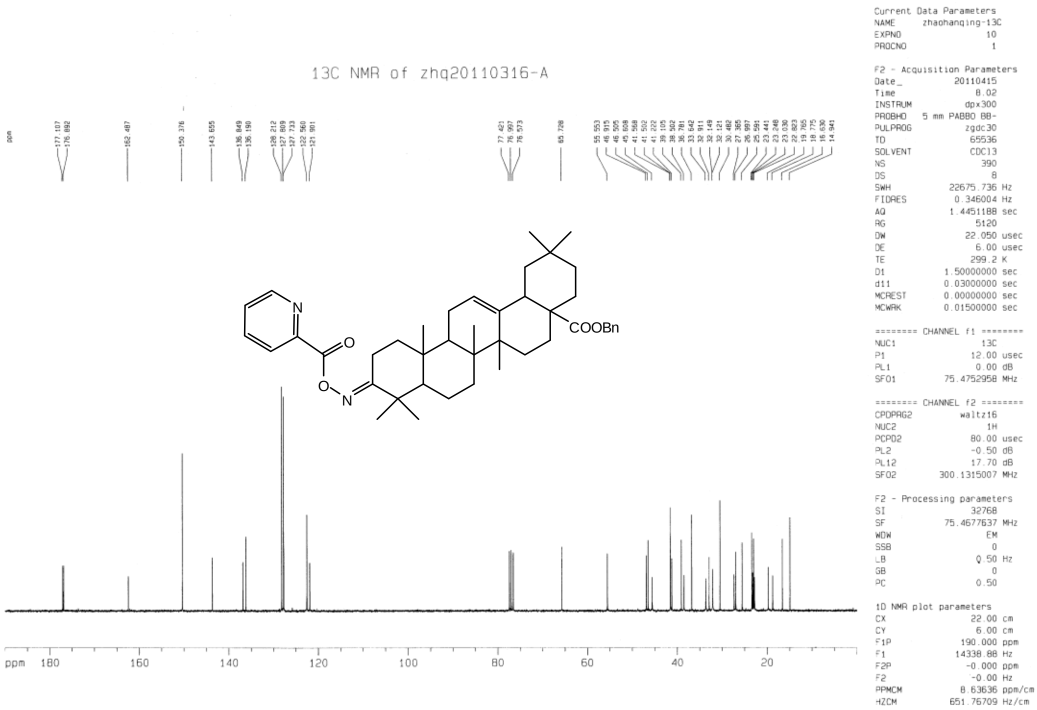

Figure S71. HRMS of compound B-14.

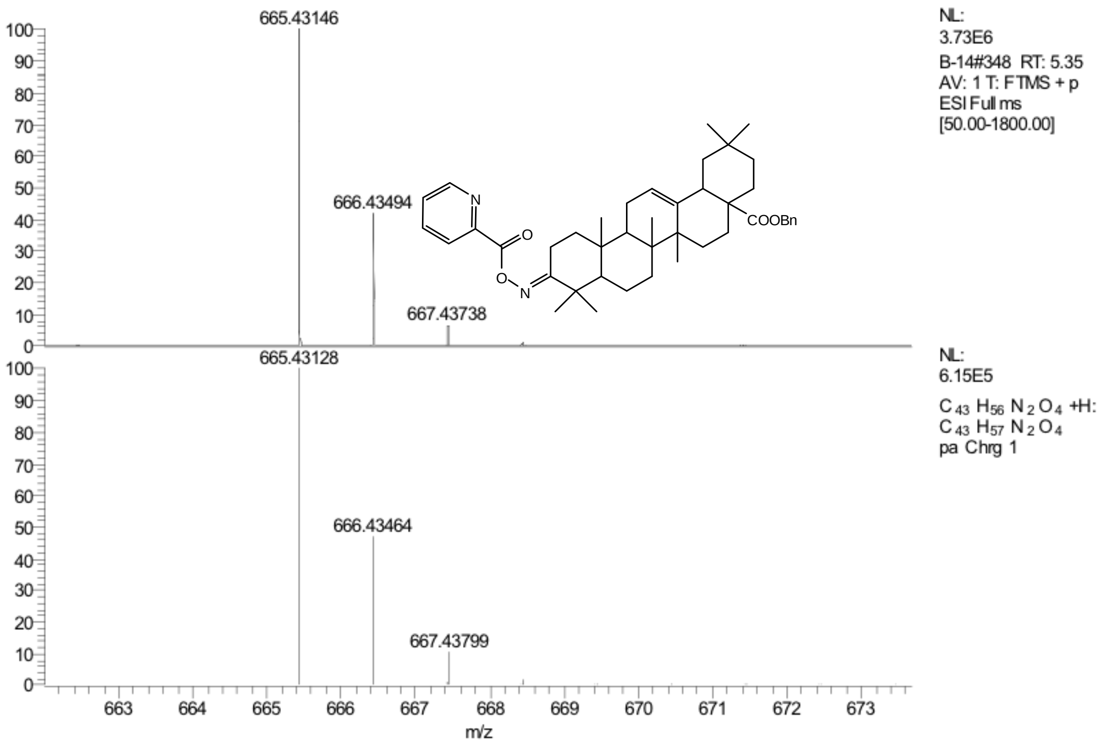

Figure S72. ^{1}H-NMR spectrum of compound B-15.

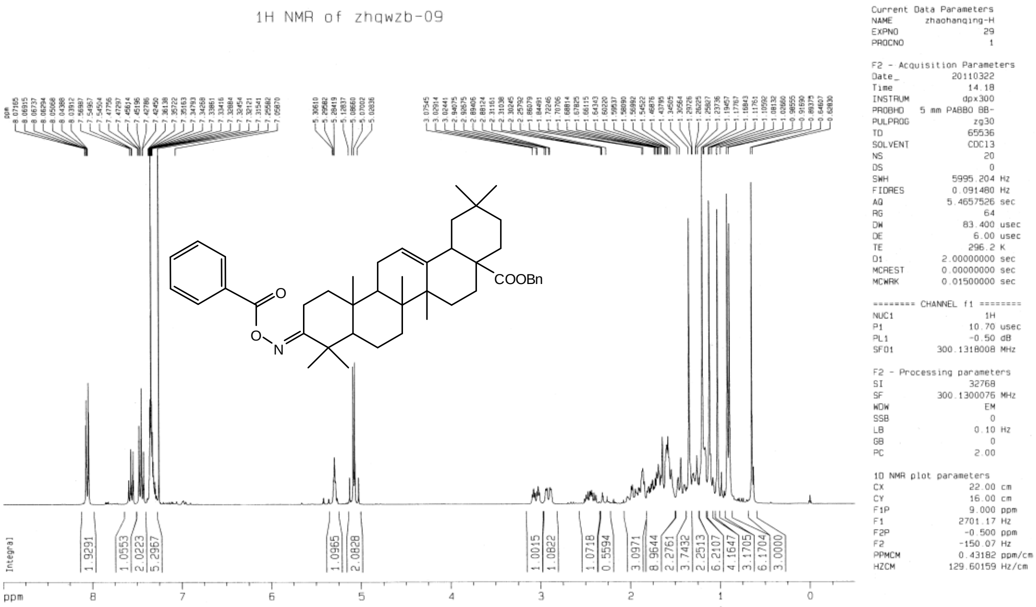

Figure S73. ^{13}C-NMR spectrum of compound B-15.

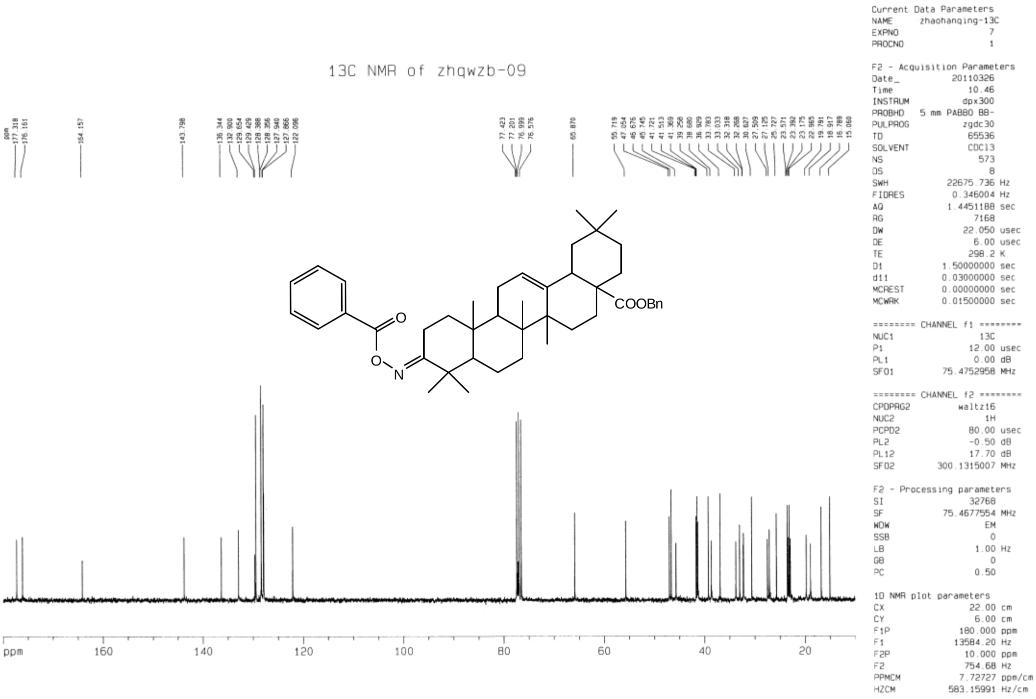

Figure S74. HRMS of compound B-15.

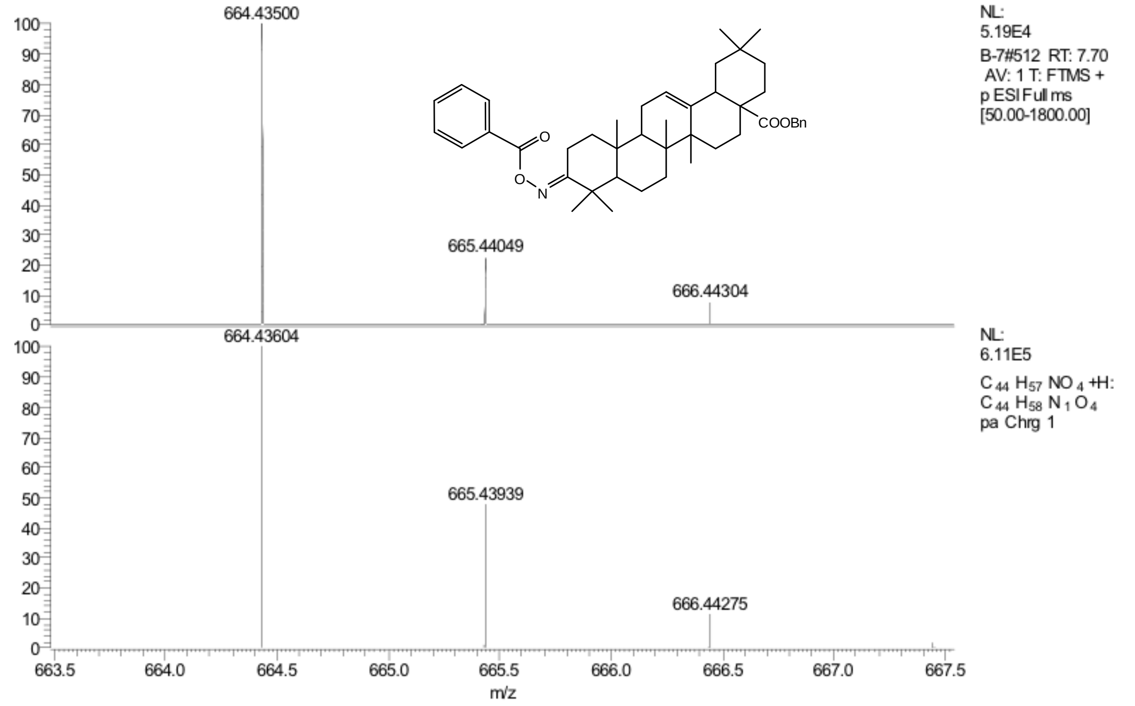

Supplement: Supplementary file 1 [file molecules-18-03615-s001.pdf]
